# Biocatalytic Enantioselective Oxidation of *Sec*‐Allylic Alcohols with Flavin‐Dependent Oxidases

**DOI:** 10.1002/adsc.201900921

**Published:** 2019-10-10

**Authors:** Somayyeh Gandomkar, Etta Jost, Doris Loidolt, Alexander Swoboda, Mathias Pickl, Wael Elaily, Bastian Daniel, Marco W. Fraaije, Peter Macheroux, Wolfgang Kroutil

**Affiliations:** ^1^ Institute of Chemistry, NAWI Graz, BioTechMed Graz University of Graz Heinrichstr. 28 8010 Graz Austria; ^2^ Institute of Biochemistry Graz University of Technology Petersgasse 12/II 8010 Graz Austria; ^3^ Chemistry of Natural & Microbial Products Department National Research Centre 33 El Buhouth St 12622 Cairo Egypt; ^4^ Molecular Enzymology Group University of Groningen Nijenborgh 4 9747AG Groningen The Netherlands; ^5^ Austrian Centre of Industrial Biotechnology, c/o Institute of Molecular Biosciences University of Graz Humboldtstraße 50 8010 Graz Austria

**Keywords:** Biocatalysis, Biotransformation, *sec*-Allylic alcohol, Asymmetric catalysis, Aerobic Oxidation

## Abstract

The oxidation of allylic alcohols is challenging to perform in a chemo‐ as well as stereo‐selective fashion at the expense of molecular oxygen using conventional chemical protocols. Here, we report the identification of a library of flavin‐dependent oxidases including variants of the berberine bridge enzyme (BBE) analogue from *Arabidopsis thaliana* (*At*BBE15) and the 5‐(hydroxymethyl)furfural oxidase (HMFO) and its variants (V465T, V465S, V465T/W466H and V367R/W466F) for the enantioselective oxidation of *sec*‐allylic alcohols. While *primary* and benzylic alcohols as well as certain sugars are well known to be transformed by flavin‐dependent oxidases, *sec*‐allylic alcohols have not been studied yet except in a single report. The model substrates investigated were oxidized enantioselectively in a kinetic resolution with an E‐value of up to >200. For instance HMFO V465S/T oxidized the (*S*)‐enantiomer of (*E*)‐oct‐3‐en‐2‐ol (**1 a**) and (*E*)‐4‐phenylbut‐3‐en‐2‐ol with E>200 giving the remaining (*R*)‐alcohol with *ee*>99% at 50% conversion. The enantioselectivity could be decreased if required by medium engineering by the addition of cosolvents (e. g. dimethyl sulfoxide).

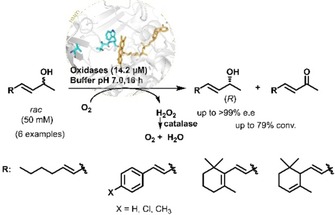

## Introduction

The oxidation of alcohols to the corresponding carbonyl compounds at the expense of molecular oxygen still belongs to the challenges in chemistry, as discussed in various recent reports and reviews using e. g. Ru‐catalysts,^1^ oxovanadium complexes,^2^ colloidal^3^ or metallic gold.^4^ Additionally to the challenge of activating molecular oxygen as oxidant, the chemoselectivity is still poorly addressed. Especially allylic alcohols are prone to various side reactions such as epoxidation, 1,3H‐shifts followed by tautomerization or polymerization.^5^ An alternative to the metal‐based oxidation, may be the biocatalytic oxidation of alcohols, including the use of alcohol dehydrogenases and oxidases.^6^ Since alcohol dehydrogenases require another enzyme for cofactor [NAD(P)^+^] recycling, oxidases using molecular oxygen as the direct oxidant would be preferred from a practical point of view.6c, 7

Oxidases have been reported for the oxidation of *prim‐*alcohols7a, 7c, 8 as well as for specific *sec*‐alcohols such as the hydroxy group of α‐hydroxy acids or sugars.7a *sec*‐Benzylic alcohols have been oxidized for instance by an aryl−alcohol oxidase,^9^ the eugenol oxidase,^10^ the L182V variant of the berberine bridge like enzyme from *Arabidopsis thaliana* (*At*BBE15),8b, 8c or the W466A/F variant of the 5‐(hydroxymethyl)furfural oxidase (HMFO) from *Methylovorus* sp.^11^ When it comes to allylic alcohols, most reports deal with *prim‐*allylic alcohols;8b *sec‐*allylic alcohols have only been reported using *At*BBE15 L182V.8b

Since oxidases provide a chiral active site, the biocatalytic oxidation of *sec*‐alcohols can be expected to display enantioselectivity, which may allow a kinetic resolution. In case it is desired that both enantiomers are oxidized, an enzyme with low enantioselectivity would be preferred.

Here, we investigate the possibility to exploit oxidases for the chemo‐ and stereo‐selective oxidation of racemic *sec*‐allylic alcohols at the expense of molecular oxygen as the only oxidant.

## Results and Discussion

### *At*BBE15

**Scheme 1:**
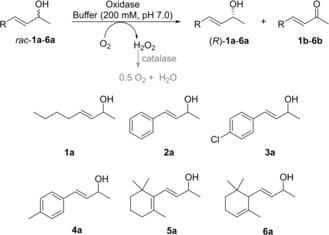
Oxidation of *rac*‐*sec‐*allylic alcohols by oxidases.

A selection of *rac*‐*sec*‐allylic alcohols bearing aromatic, aliphatic and cyclic moieties with and without an additional conjugated C=C double bond (**5 a**, **6 a**) were chosen as substrates (Scheme 1). The above mentioned *At*BBE15 L182V variant, which needs to be expressed in *Komagataella phaffii* (formerly classified as *Pichia pastoris*), was the starting point as catalyst for our investigation.8b, 12

**Figure 1:**
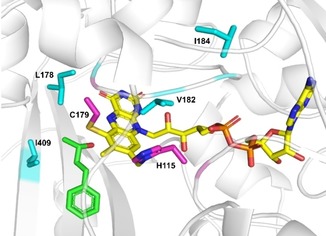
Docking of substrate **2 a** (in green) into the active site of *At*BBE15 (PDB 4UD8). The flavin cofactor is shown in yellow with its bicovalent linkage to His115 and Cys179 (shown in pink). Residues selected for site‐directed mutagenesis are highlighted in blue (L178, L182, I184 and I409). The figure was prepared using PyMol.

In *At*BBE15, the FAD cofactor is bi‐covalently bound to the enzyme backbone (Figure 1). The apolar residues L178 and I409 in the active site were chosen for replacement to the less bulky amino acid valine to see the influence of these positions on the activity and stereoselectivity. The exchange I184V was speculated to improve the oxidase activity of the enzyme.

Comparing the initial variant (L182V, the L182V exchange enables the use of molecular oxygen), with the I409V variant, I409V led, in general, to lower conversion for the substrates investigated (Table 1). The I184V exchange did not improve oxidase activity. The L182V as well as the other variants oxidized preferentially the (*S*)‐enantiomer leaving the (*R*)‐enantiomer. The calculated enantioselectivity varied depending on the variant as well as the substrate, e. g. the enantioselectivity E was >200 with *At*BBE15 L182V for **1 a** and **3 a**, but low for **2 a** and **4 a** (E=49 and 35, respectively), while the additional mutation I409V led to low E‐value only for **2 a** (E=26), but was high for **1 a**, **3 a** and **4 a** (E>200).

**Table 1:** Oxidation of allylic *rac*‐*sec*‐alcohols using *At*BBE15 L182V variants.^[a]^

Substr.	Variant of *At*BBE15 L182V	conv. [%]	*ee*_s_ [%]^[b]^	E
**1 a**	– ^[c]^	50^[d]^	>99 (*R*)	>200
**1 a**	I409V^[e]^	10	11 (*R*)	>200
**2 a**	– ^[c]^	55^[d]^	>99 (*R*)	49
**2 a**	L178V/I184V^[e]^	<1	n.d.^[f]^	n.d.^[f]^
**2 a**	I409V^[e]^	8	8 (*R*)	26
**3 a**	– ^[c]^	50	>99 (*R*)	>200
**3 a**	L178V/I184V^[e]^	14	16 (*R*)	135
**3 a**	I409V^[e]^	34	51 (*R*)	>200
**4 a**	– ^[c]^	57	>99 (*R*)	35
**4 a**	L178V/I184V^[e]^	17	20 (*R*)	102
**4 a**	I409V^[e]^	44	78 (*R*)	>200
**5 a**	I409V^[e]^	8	8 (*R*)	26

^[a]^ Condition: KPi‐buffer (200 mM, pH 7.0) containing the oxidases (1.67 μM in case of L178V/I184V variant and 16.7 μM in case of I409V variant and *At*BBE15 L182V, final concentration in 500 μL reaction volume in 4 mL glass vials), catalase from *Micrococcus lysodeikticus* (15 μL, 170000 U/mL), the substrate (50 mM). The reaction mixtures and blanks were shaken for 16 hours (170 rpm, 21 °C) and extracted with ethyl acetate (2×300 μL), dried with Na_2_SO_4_ and measured by GC‐FID. ^[b]^ *Ee_s_* values for **1 a** were measured by using GC on a chiral phase. *ee_s_* values for **2 a**–**5 a** were measured by using HPLC using a chiral column. ^[c]^ Contains the L182V exchange only. ^[d]^ This substrate has already been reported with *At*BBE15 L182V.8b ^[e]^ Performed in the presence of 2 bar oxygen pressure. ^[f]^ Not determined due to low conversion.

### HMFO

To create a library of oxidases for the oxidation of *sec*‐allylic alcohols, we extended our research to another flavin‐dependent oxidase previously described mainly for the oxidation of selected *prim‐*alcohols, the 5‐hydroxymethylfurfural oxidase (HMFO).^11^, ^13^

In contrast to *At*BBE15, the FAD in HMFO is not covalently bound and the enzyme can efficiently be produced in *E. coli*.13a In addition to its oxidation activity to produce the polymer building block, 2,5‐furandicarboxylic acid (FDCA) from 5‐(hydroxymethyl)furfural (HMF),11b HMFO is active toward a wide range of benzylic or allylic *prim*‐alcohols and aldehydes13b and its variants are able to transform *sec*‐benzylic alcohols in a stereoselective fashion.^9^, 11a, 14 Furthermore, the oxidation activity of HMFO on *prim‐* and *sec‐*thiols has been described recently,^15^ but no activity for *sec*‐allylic alcohols has been reported for this enzyme, yet.

**Figure 2:**
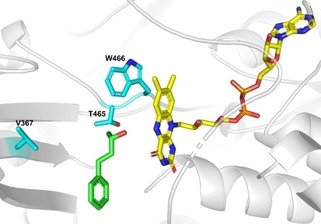
Docking of substrate **2 a** (in green) into HMFO V465T (PDB 6F97). The FAD is shown in yellow and residues selected for site‐directed mutagenesis are highlighted in blue (V367, T465, and W466). Docking was performed with Yasara. The figure was prepared using PyMol.15b

When substrates **1 a**–**5 a** were tested with the wild type enzyme HMFO, only moderate conversions were observed at the conditions employed (Table 2, entries 1, 6, 11, 16, 21). Nevertheless, it is worth noting, that exclusively oxidation of the allylic alcohol to the α,β‐unsaturated ketone was observed, thus side reactions like epoxidation did not occur. Assuming that the low conversion was due to a slow transformation caused by steric hindrance in the active site of the enzyme, variants V465T/S were investigated (Figure 2), which proved already to be useful for the oxidation of *sec*‐thiols by reducing steric hindrance.15b Furthermore, the double variant V465T/W466H was tested as well as a previously published variant V367R/W466F.11a

**Table 2:** Oxidation of *rac*‐*sec*‐allylic alcohols with variants of HMFO.^[a]^

Entry	Substr.	Variant	Conv. [%]	*ee*_s_ [%]^[b]^	E
1	**1 a**	wt	16	19 (*R*)	>200
2	**1 a**	V465T	50	>99 (*R*)	>200
3	**1 a**	V465S	50	>99 (*R*)	>200
4	**1 a**	V465T/W466H	25	33 (*R*)	>200
5	**1 a**	V367R/W466F	18	21 (*R*)	55
6	**2 a**	wt	29	25 (*R*)	5
7	**2 a**	V465T	50	>99 (*R*)	>200
8	**2 a**	V465S	50	>99 (*R*)	>200
9	**2 a**	V465T/W466H	50	99 (*R*)	>200
10	**2 a**	V367R/W466F	33	34 (*R*)	8
11	**3 a**	wt	10	10 (*R*)	21
12	**3 a**	V465T	48	94 (*R*)	>200
13	**3 a**	V465S	50	99 (*R*)	>200
14	**3 a**	V465T/W466H	50	96 (*R*)	>200
15	**3 a**	V367R/W466F	46	83 (*R*)	>200
16	**4 a**	wt	13	14 (*R*)	35
17	**4 a**	V465T	48	92 (*R*)	>200
18	**4 a**	V465S	48	96 (*R*)	>200
19	**4 a**	V465T/W466H	50	98 (*R*)	>200
20	**4 a**	V367R/W466F	32	45 (*R*)	70
21	**5 a**	wt	4	2	n.d.^[c]^
22	**5 a**	V465T	32	44 (*R*)	46
23	**5 a**	V465S	38	58 (*R*)	65
24	**5 a**	V465T/W466H	4	n.d.^[c]^	n.d.^[c]^
25	**5 a**	V367R/W466F	4	n.d.^[c]^	n.d.^[c]^

^[a]^ Condition: KPi‐buffer (200 mM, pH 7.0) containing the oxidases (14.2 μM final concentration in 500 μL reaction volume in 4 mL glass vials), catalase from *Micrococcus lysodeikticus* (15 μL, 170000 U/mL), substrate (50 mM). The reaction mixtures were shaken for 16 hours (170 rpm, 21 °C) and extracted with ethyl acetate (2×300 μL), dried with Na_2_SO_4_ and analyzed by GC‐FID. Conversions were measured based on area ratio of ketone to substrate. Reactions were conducted in duplicate. ^[b]^ *Ee_s_* values for **1 a** were measured by using GC equipped with chiral column. *Ee_s_* values for **2 a**–**5 a** were measured by using HPLC equipped with a chiral column. ^[c]^ Not determined due to low conversion.

The two variants V465T and V465S oxidized all five substrates **1 a**–**5 a** very efficiently, reaching in most cases 50% conversion (at 50 mM substrate, 16 h) and up to >99% *ee*. The latter indicated excellent enantioselectivity, thus only one enantiomer was preferentially transformed. All variants showed preference to oxidize the (*S*)‐enantiomer leaving the (*R*)‐enantiomer, which corresponds to the same stereopreference as observed with *At*BBE15 L182V.

### O_2_ Pressure Study

Since the oxidant is gaseous molecular oxygen, an increase in its concentration in the buffer may lead to improved conversion. Consequently, the reactions were tested in the presence of 2 and 4 bar of molecular oxygen and compared to the reaction performed in air at ambient conditions (Table 3, for substrates **1 a**–**2 a**; Table S9 for substrates **3 a**–**5 a**). Interestingly the conversion increased at higher pressure for the wild type for substrate **2 a**–**5 a**, while this was not the case for substrate **1 a**. In general, the variants could stand higher pressure, however, since the handling is more demanding in terms of equipment and also more time consuming, the ambient reaction conditions were preferred. Furthermore, and probably even more importantly, it turned out that the enantioselectivity expressed as the E‐value decreased at elevated pressure compared to the reaction performed in the absence of pressure. For instance, HMFO V465S displayed an E‐value of 29 for the oxidation of **4 a** at ambient pressure in the presence of 10% glycerol as cosolvent, while already at 1.5 bar of molecular oxygen, the enantioselectivity decreased to a value of 5 (Table 4).

**Table 3:** Oxidation of *rac*‐*sec*‐allylic alcohols **1 a**–**2 a** employing HMFO variants in the presence of air, 2 and 4 bar O_2_ pressure.^[a]^

Entry	Substr.	Variant	Conv. [%]		
			air	O_2_ (2 bar)	O_2_ (4 bar)
1	**1 a**	wt	16	13	10
2	**1 a**	V465T	50	31	26
3	**1 a**	V465 S	50	35	26
4	**1 a**	V465T/W466H	25	18	9
5	**1 a**	V367R/W466F	18	9	7
6	**2 a**	wt	29	33	35
7	**2 a**	V465T	50	48	45
8	**2 a**	V465 S	50	48	44
9	**2 a**	V465T/W466H	50	48	46
10	**2 a**	V367R/ W466F	33	34	46

^[a]^ Condition: KPi‐buffer (200 mM, pH 7.0) containing the oxidases (14.2 μM final concentration in 500 μL reaction volume in 4 mL glass vials), catalase from *Micrococcus lysodeikticus* (15 μL, 170000 U/mL), **1 a**–**2 a** (50 mM). The reaction mixtures were shaken for 16 hours (170 rpm, 21 °C) and extracted with ethyl acetate (2×300 μL), dried with Na_2_SO_4_ and analyzed by GC‐FID. Conversions were measured based on area ratio of ketone to substrate. Reactions were done in duplicate.

**Table 4:** Enantioselectivity of oxidation of substrate **4 a** using HMFO variants at ambient air pressure and at 1.5 bar O_2_.^[a]^

Entry	Variant	O_2_ [bar]	Conv. [%]	*ee_s_* [%]^[b]^	E
1	V465S	1.5	58	62	5
2	V465S	ambient	55	95	29
3	V465T	1.5	54	73	9
4	V465T	ambient	50	73	14

^[a]^ Condition: KPi‐buffer (200 mM, pH 7.0) containing the oxidases (2.1 μM final concentration in 1 mL reaction volume in 4 mL glass vials), catalase from *Micrococcus lysodeikticus* (30 μL, 170000 U/mL), the substrate (50 mM), 10% v/v glycerol as cosolvent. The reaction mixtures were shaken for 16 hours (170 rpm, 21 °C; additional 1.5 bar O_2_ for the mixtures with O_2_ pressure) and extracted with ethyl acetate (2×500 μL), dried with Na_2_SO_4_ and analyzed by GC‐FID and HPLC. ^[b]^ *Ee_s_* values were measured by using HPLC equipped with chiral column.

When testing the most suitable HMFO variants (V465S, V465T) as well as V367R/W466F to oxidize other non‐allylic *secondary* alcohols, it turned out that none of the variants was able to oxidize *sec*‐alcohols such as 2‐octanol or 1‐phenyl‐2‐propanol. The benzylic alcohol 1‐phenylethanol was oxidized only by V465T. Therefore, we hypothesized that the HMFO variants (V465S, V465T) are chemoselective, differentiating between allylic and non‐allylic *sec*‐alcohols. Furthermore, none of these variants was able to oxidize the *primary* alcohol 2‐phenyl‐1‐ethanol either, while 1‐octanol was oxidized by V465S and V465T but not V367R/W466F.

### Solvent Study

Furthermore, the oxidation of various substrates was tested in the presence of various organic solvents (Table 5). Interestingly, in general, the organic solvents tested were compatible with the enzyme, independent whether a water miscible or immiscible organic solvent was used. It is worth noting that the *prim*‐alcohols ethanol and methanol could be used as cosolvents. Glycerol possessing two *prim*‐ and a *sec*‐alcohol functionality could also be used, whereby it turned out that the reactions in glycerol are faster than in DMSO (SI, Table S4). Thus, glycerol did not inhibit the oxidation but led to lower enantioselectivity for substrates **3 a**–**5 a** which enabled to reach higher conversions (e. g. 72%, entry 25, Table 5). While substrate **1 a** was converted with an E‐value >200 in the presence of all cosolvents, the other substrates were transformed with a significant decrease in enantioselectivity E in the presence of DMSO. Thus, additionally to the (*S*)‐enantiomer also the (*R*)‐enantiomer was oxidized. The E‐value for substrate **5 a** with DMSO and glycerol was found to be 5 and 4, respectively. Consequently, 68% of conversion was obtained in the case of substrate **5 a** in the presence of glycerol as cosolvent. Similarly, substrate **3 a** was transformed in glycerol with low E‐value, leading to higher conversion (72%) compared to other solvents. Compound **6 a** was in general not well accepted leading only to low conversion (3%, Table S8).

**Table 5:** Oxidation of *rac*‐allylic alcohols with HMFO V465S in the presence of 5% v/v organic solvents.^[a]^

Entry	Substr.	Cosolvents	Conv. [%]	*ee*_s_ [%]^[b]^	E
1	**1 a**	DMSO	51	>99 (*R*)	>200
2	**1 a**	isooctane	50	>99 (*R*)	>200
3	**1 a**	glycerol	51	>99 (*R*)	>200
4	**1 a**	*n*‐heptane	50	>99 (*R*)	>200
5	**1 a**	MeOH	51	>99 (*R*)	>200
6	**1 a**	EtOH	51	>99 (*R*)	>200
7	**1 a**	*i*PrOH	51	>99 (*R*)	>200
8	**1 a**	2‐butanone	52	>99 (*R*)	>200
9	**1 a**	acetone	51	>99 (*R*)	>200
10	**1 a**	DMF	50	>99 (*R*)	>200
11	**1 a**	dioxane	50	>99 (*R*)	>200
12	**2 a**	DMSO	65	98 (*R*)	14
13	**2 a**	isooctane	49	93 (*R*)	>200
14	**2 a**	glycerol	51	99 (*R*)	>200
15	**2 a**	*n*‐heptane	50	95 (*R*)	146
16	**2 a**	MeOH	49	96 (*R*)	>200
17	**2 a**	EtOH	50	92 (*R*)	79
18	**2 a**	*i*PrOH	50	95 (*R*)	146
19	**2 a**	2‐butanone	49	85 (*R*)	44
20	**2 a**	acetone	48	83 (*R*)	49
21	**2 a**	DMF	55	98 (*R*)	41
22	**2 a**	dioxane	50	86 (*R*)	37
23	**3 a**	DMSO	49	74 (*R*)	17
24	**3 a**	isooctane	36	54 (*R*)	84
25	**3 a**	glycerol	72	99 (*R*)	11
26	**3 a**	*n*‐heptane	42	62 (*R*)	24
27	**3 a**	MeOH	38	51 (*R*)	18
28	**3 a**	EtOH	36	54 (*R*)	84
29	**3 a**	*i*PrOH	50	70 (*R*)	12
30	**3 a**	2‐butanone	42	70 (*R*)	124
31	**3 a**	acetone	42	70 (*R*)	124
32	**3 a**	DMF	37	56 (*R*)	74
33	**3 a**	dioxane	40	44 (*R*)	7
34	**4 a**	DMSO	67	>99 (*R*)	14
35	**4 a**	isooctane	63	>99 (*R*)	18
36	**4 a**	glycerol	55	>99 (*R*)	49
37	**4 a**	*n*‐heptane	79	>99 (*R*)	7
38	**4 a**	MeOH	53	>99 (*R*)	80
39	**4 a**	EtOH	50	>99 (*R*)	>200
40	**4 a**	*i*PrOH	51	>99 (*R*)	>200
41	**4 a**	2‐butanone	50	>99 (*R*)	>200
42	**4 a**	acetone	53	>99 (*R*)	80
43	**4 a**	DMF	52	>99 (*R*)	116
44	**4 a**	dioxane	58	>99 (*R*)	31
45	**5 a**	DMSO	17	13 (*R*)	5
46	**5 a**	isooctane	20	20 (*R*)	11
47	**5 a**	glycerol	68	76 (*R*)	4
48	**5 a**	*n*‐heptane	20	19 (*R*)	9
49	**5 a**	MeOH	18	17 (*R*)	9
50	**5 a**	EtOH	11	10 (*R*)	10
51	**5 a**	*i*PrOH	19	17 (*R*)	7
52	**5 a**	2‐butanone	17	16 (*R*)	10
53	**5 a**	acetone	27	30 (*R*)	13
54	**5 a**	DMF	22	22 (*R*)	10
55	**5 a**	dioxane	19	19 (*R*)	11

^[a]^ Condition: KPi‐buffer (200 mM, pH 7.0) containing the oxidases (14.2 μM final concentration in 1 mL reaction volume in 4 mL glass vials), catalase from *Micrococcus lysodeikticus* (30 μL, 170000 U/mL), the substrate (50 mM), 5% v/v various co‐solvents. The reaction mixtures were shaken for 16 hours (170 rpm, 21 °C) and extracted with ethyl acetate (2×500 μL), dried with Na_2_SO_4_ and analyzed by GC‐MS. Conversions were measured based on area ratio of ketone to substrate. ^[b]^ *Ee_s_* values for **1 a** were measured by GC on a chiral phase. *ee_s_* values for **2 a**–**5 a** were measured by HPLC on a chiral phase.

For obtaining semi‐preparative amounts of the products **1 b**–**5 b**, experiments were performed with 12.5 mmol at 50 mM substrate concentration employing the variant V465S (Table 6). After purification, the isolated yields were determined and NMR analysis proved the structure and the purity of the isolated ketones (**1 b**–**5 b**).

**Table 6:** Semi‐preparative scale oxidation using HMFO V465S.^[a]^

Entry	Substr.	Conv. [%]	Isolated yields b [%]	*ee*_s_ [%]	E
1	**1 a**	50	70^[b]^	>99	>200
2	**2 a**	53	33	92	32
3	**3 a**	35	54	35	7
4	**4 a**	52	64	92	40
5	**5 a**	31	31	35	11

^[a]^ Condition: KPi‐buffer (200 mM, pH 7.0) containing the oxidase (14.2 μM final concentration in 25 mL reaction volume), catalase from *Micrococcus lysodeikticus* (750 μL, 170000 U/mL) and the substrate (50 mM). The reaction mixtures were shaken for 48 h (170 rpm, 21 °C) and extracted with ethyl acetate (2×50 mL), dried with Na_2_SO_4_ and analyzed by GC‐MS. Conversions were measured based on area ratio of ketone to substrate. The percentage of isolated yield refers to the conversion achieved. ^[b]^ The remaining substrate was isolated in quantitative yield with respect to the observed conversion.

## Conclusion

The biocatalytic oxidation of *sec*‐allylic alcohols to the corresponding allylic ketones represents a valuable alternative for chemical methods, which often require harsh conditions and suffer from poor chemo‐ and enantio‐selectivity. In the current study, the O_2_‐dependent oxidation of *sec*‐allylic alcohols was performed using flavin‐dependent alcohol oxidases namely 5‐(hydroxymethyl)furfural oxidase and *At*BBE15 and variants thereof.

The created library of oxidases allows the chemoselective oxidation of allylic alcohols to the corresponding α,β‐unsaturated ketones without any detectable side reaction such as epoxidation, polymerization or hydride shifts. From the oxidases tested possessing a covalently bound FAD, *At*BBE15 L182V turned out to be the most suitable. From the HMFO variants tested, the two variants V465S and V465T led to the highest conversions (up to 50%) and excellent enantioselectivity (E>200) for the oxidation of **1 a**–**4 a**. All oxidases investigated preferentially oxidized the (*R*)‐allylic alcohol leaving the (*S*)‐enantiomer. Especially the HMFO V465S/T variant showed high enantioselectivity (E>200) for most substrates (except **5 a**). The enantioselectivity could be tuned by applying either pressure or by the addition of cosolvents. For instance, the addition of DMSO as cosolvent led to a decrease in enantioselectivity, which was associated with significantly higher conversions (up to 79%) for selected substrates. Thus, the oxidases may be employed for non‐enantioselective oxidation as well as for enantioselective oxidation of allylic alcohols.

## Experimental Section

### Synthesis of Allylic Alcohols from their Corresponding Ketones

Substrates (*E*)‐oct‐3‐en‐2‐ol (**1 a**), (*E*)‐4‐phenylbut‐3‐en‐2‐ol (**2 a**), (*E*)‐4‐(4‐chlorophenyl)but‐3‐en‐2‐ol (**3 a**) and (*E*)‐4‐(4‐methylphenyl)but‐3‐en‐2‐ol (**4 a**) were synthesized from their corresponding ketones. To a solution of various ketones in methanol (30 mL), sodium borohydrate was slowly added on ice (see the details in Table S1). The reaction mixture was stirred for 2 hours and formation of the product was monitored by TLC. When the reaction was completed, quenching was done by using saturated aqueous NH_4_Cl (15 mL). Then the resultant mixture was concentrated under reduced pressure and the residue was extracted with ethyl acetate (3×20 mL). The combined organic fractions were washed with brine, dried with Na_2_SO_4_ and concentrated under reduced pressure. Purification of the residue was done by flash chromatography (8:2, *c*‐hexane:EtOAc).

### Preparation of the Biocatalysts

#### HMFO wt (pEG 387), HMFO V465S (pEG 392), HMFO V465T (pEG 393), HMFO W466H (pEG 390) and HMFO V465T/W466H (pEG 395)

For the different variants of HMFO, the same expression and purification method was used as it follows:

***Expression***: For HMFO expression, an overnight culture of *E. coli* BL21(DE3) cells bearing the previously prepared SUMO‐HMFO encoding plasmid (ChampionTM pET SUMO) in 200 mL of Terrific Broth containing 50 μg/mL kanamycin and grown at 37 °C until it reached an OD_600_ of 0.8–1.0. Cells were induced with isopropyl‐*β*‐D‐thiogalactopyranoside (IPTG, 1.0 mM) and grown overnight at 20 °C. Cells were harvested by centrifugation at 3730 g for 15 min (Hettich® Rotina 420R centrifuge, 4 °C) and resuspended in Tris−HCl (35 mL, 100 mM, pH 8.0) supplemented with glycerol (10% v/v), NaCl (150 mM), and FAD (10 μM). The cell extract was obtained by sonication with a Branson Digital Sonifier 250 (30% amplitude, 2 min, 1 sec pulse, 4 sec pause). The lysate was cleared by centrifugation (20000×g for 15 min).

***Purification***: His‐Tagged HMFO was purified by immobilized Ni‐affinity chromatography (5 mL HisTrap FF column, GE Healthcare) following standard protocols with a 5 to 500 mM gradient of imidazole (binding buffer: Tris−HCl, 50 mM, pH 8.0 containing 150 mM NaCl and 5 mM imidazole; elution buffer: Tris−HCl, 50 mM, pH 8.0 containing 150 mM NaCl and 500 mM imidazole). Fractions containing HMFO were pooled concentrated by ultrafiltration (20 mL, 50 kDa cut‐off, Vivaspin) and desalted (SephadexTM G‐25M, GE Healthcare). After desalting the fractions were shock frosted in liquid nitrogen and stored at −20 °C. For activity tests the lyophilized enzyme preparation were dissolved in potassium phosphate buffer (100 mM, pH 7.0) without cleaving off the SUMO‐tag. For running the biotransformations, the lyophilized pure enzymes were rehydrated in the reaction buffer just before using them, then the concentration of each variants was measured by Bradford assay.

***Site‐Directed Mutagenesis***: Site‐directed mutagenesis of the wild‐type HMFO gene was performed using two‐step whole‐plasmid PCR. For the creation of V465T/W466H, the HMFO‐ W466H plasmid was used as template. The primers were ordered at IDT (Leuven, Belgium). After three cycles of linear PCR, the mixture containing the forward primer and the mixture with the reverse primer were combined for additional 15 cycles. Template DNA was cleaved with DpnI (New England Bio‐Labs, Ipswich, MA, USA). The plasmid was purified with a PCR purification kit (Qiagen, Hilden, Germany) and transformed into *E. coli* TOP10 cells. The introduction of the mutations was confirmed by sequencing.

#### *At*BBE15 Variants

***Creation of variants***: *At*BBE15 L182V originates from previous work and was used as template.^12^ Using primer pair fw GATTCACCGTTAACGGTTTGGAACCCTTAC and rw GTAAGGGTTCCAAACCGTTAACGGTGAATC the *At*BBE‐like 15 variant L182V/L409V was created using the Quick Change Protocol. For the *At*BBE‐like 15 L178V/I182V/I184V variant a synthetic gene was ordered from Geneart (Regensburg, Germany) composed of sequences encoding the α‐factor and the enzyme. The gene was clone to the pPICZα vector for expression.

***Expression***: The expressions of *At*BBE15 L182V was performed as described before.^12^ *At*BBE‐like15 L182V/I409V and *At*BBE‐like15 L178V/L182V/I184V were expressed in shake flask using minimal media. The compositions of every used media are listed in Table S2 and the components are listed in Table S3. 50 mL of sterile BMD medium was added to 300 mL Erlenmeyer flasks and inoculated with the respective expression strains. The cells were grown for 72 hours (300 rpm, 28 °C). After 72 hours expression was induced using 5 mL of BMM10 medium. Subsequently every 12 hours, 50 μL of absolute methanol was added for 72 hours.

***Purification***: The cells were removed using an Eppendorf centrifuge 5810 R (Eppendorf AG, Hamburg, Germany) at 2800 g at 4 °C for 15 minutes. A 5 mL Ni−NTA fast flow column (GE Healthcare, Chicago, Illinois, USA) was equilibrated with 50 mM potassium phosphate buffer containing 10 mM imidazole pH 8. The supernatant was loaded to the column using a peristaltic pump at a flow rate of 15 mL/min. The column was washed with 20 mL 50 mM potassium phosphate buffer containing 20 mM imidazole pH 8, subsequently with 20 mL 50 mM potassium phosphate buffer containing 40 mM imidazole pH 8. The enzymes were eluted using 50 mM potassium phosphate buffer containing 150 mM imidazole pH 8 and dialysed over night against 50 mM potassium phosphate buffer pH 8. Finally, the enzyme was concentrated using Amicon® Ultra Centrifugal Filters (Merck KGaA, Darmstadt Germany) and shock frozen in liquid nitrogen.

### General Procedure for Oxidation of Sec‐Allylic Alcohols by Using Oxidases

The oxidation reactions were run in 4 mL glass vials under the conditions described below:

For HMFO the oxidase (2.1 to 14.2 μM final concentration in 1 mL reaction volume), catalase from *Micrococcus lysodeikticus* (30 μL, 170000 U/mL), the substrate (50 mM final concentration) and 5% to 50% v/v of various cosolvents (in case of cosolvent study) were added to the buffer (KPi, 200 mM, pH 7.0). The biotransformation vials were incubated for 16 hours at 21 °C (170 rpm, vertical shaking). The extraction was done with ethyl acetate (2×500 μL). Combined organic phases were dried with Na_2_SO_4_. Samples were prepared from the dried organic phase without further treatment and measured on GC‐MS and GC‐FID. When no cosolvent was used, the reaction volume was reduced to 500 μL and 15 μL of catalase (170000 U/mL) was used. In this case, the extraction was done with ethyl acetate (2×300 μL). The rest of the procedure was the same. In case of applying oxygen pressure, the glass vials were left unscrewed and fixed in a rack in the oxygen chamber and the oxygen pressure was applied. Conversions were measured based on area ratio of ketone to substrate in each biotransformation mixture by using GC without addition of any extra compound as internal standard.

### Determination of Optical Purity

The enantiomeric excess of remaining alcohols was analyzed by HPLC (in case of substrates **2 a**–**5 a**, see Tables S10–S13) and GC (in case of **1 a**, see Tables S14–S15) on a chiral phase. Absolute configurations were assigned by comparison of elution order of enantiomers on chiral HPLC and chiral GC with published data.^16^ For determination of the absolute configuration of **1 a**, by comparison of the elution order on chiral GC with literature data, the alcohol moiety had to be acetylated.^17^ For that purpose, derivatization was performed by adding 4‐(*N*,*N*‐dimethylamino)pyridine (5 mg) dissolved in acetic anhydride (100 μL). After washing with water, drying with Na_2_SO_4_ and measuring on chiral GC, the stereopreference of the enzyme toward **1 a** substrate was confirmed.

### Docking Experiments

Each enzyme was prepared in PyMol, removing all water molecules present in the enzyme structure. The substrate (*R*)‐**2 a** was separately prepared in Yasara.

For docking, the adapted structure file was loaded to Yasara. The N5‐atom of FAD was chosen as the center of the simulation cell with a 10 Å diameter defined around the selected atom. AMBER03 was chosen as the force field. The substrate was added to the prepared file and the energy minimization experiments were run. Afterwards the docking experiments were performed using docking parameters including Autodock VINA, 25 docking runs, Cluster RMSD 5.00 Å. The outcome was analyzed in PyMol.

## Supporting information

As a service to our authors and readers, this journal provides supporting information supplied by the authors. Such materials are peer reviewed and may be re‐organized for online delivery, but are not copy‐edited or typeset. Technical support issues arising from supporting information (other than missing files) should be addressed to the authors.

Supplementary
