# Supplementary material for: Biocatalytic Enantioselective Oxidation of Sec‐Allylic Alcohols with Flavin‐Dependent Oxidases
Source: Adv Synth Catal. 2019 Oct 10;361(22):5264–71. doi: 10.1002/adsc.201900921 (PMC6919931; doi:10.1002/adsc.201900921)
Supplement: Supplementary file 1 — Supplementary [file ADSC-361-5264-s001.pdf]

Advanced Synthesis & Catalysis

Supporting Information

© Copyright Wiley-VCH Verlag GmbH & Co. KGaA, 69451 Weinheim, 2019

Biocatalytic Enantioselective Oxidation of *Sec*-Allylic Alcohols with Flavin-Dependent Oxidases

Somayyeh Gandomkar, Etta Jost, Doris Loidolt, Alexander Swoboda, Mathias Pickl, Wael Elaily, Bastian Daniel, Marco W. Fraaije, Peter Macheroux, and Wolfgang Kroutil*
© 2019 The Authors. Published by Wiley-VCH Verlag GmbH & Co. KGaA.
This is an open access article under the terms of the Creative Commons Attribution License, which permits use, distribution and reproduction in any medium, provided the original work is properly cited.

Biocatalytic Enantioselective Oxidation of *sec*-Allylic Alcohols with Flavin-Dependent Oxidases

Somayyeh Gandomkar,[a] Etta Jost,[a] Doris Loidolt,[a] Alexander Swoboda,[a] Mathias Pickl,[a] Wael Elaily,[b,c] Bastian Daniel,[b,§] Marco W Fraaije,[d] Peter Macheroux,[b] Wolfgang Kroutil [a] *

[a] Institute of Chemistry, NAWI Graz, BioTechMed Graz, University of Graz, Heinrichstr. 28, 8010 Graz, Austria
Corresponding author: Wolfgang Kroutil, Tel: +43-316-380-5350; Fax: +43-316-380-9840; E-mail: wolfgang.kroutil@uni-graz.at

[b] Institute of Biochemistry, Graz University of Technology, Petersgasse 12/II, 8010 Graz, Austria

[c] Chemistry of Natural & Microbial Products Department, National Research Centre, 33 El Buhouth St, 12622 Cairo, Egypt

[d] Molecular Enzymology Group, University of Groningen, Nijenborgh 4, 9747AG, Groningen, The Netherlands

§ Current address: Austrian Centre of Industrial Biotechnology, c/o Institute of Molecular Biosciences, University of Graz, Humboldtstraße 50, 8010 Graz, Austria

Supplementary Information

Table of Contents

1 Material and methods 3

1.1. Enzymes and chemicals 3

1.2. Synthesis 3

1.2.1. Synthesis of allylic alcohols from their corresponding ketones 3

1.2.2. NMR of the allylic alcohols synthesized 3

1.3. Biocatalytic reactions 5

1.3.1. Preparation of the biocatalysts 5

2 Results 15

2.1. Biotransformations 15

2.1.1. Cosolvent study with substrate **4a** and HMFO variants 15

2.1.2. Investigating the oxygen pressure effect on the oxidation of various substrates by using two different variants of HMFO 19

2.1.3. NMR of purified ketones from upscaling reaction with HMFO V465S 21

3 Analytics 22

4 Supplementary 25

4.1. NMRs 25

4.2. GC-MS chromatograms 33

4.3. HPLC chromatograms 40

4.4. GC chromatograms 54

5 References 57

1. Material and methods

1.1. Enzymes and chemicals

Reagents and organic solvents were obtained from commercial suppliers in reagent grade quality and used without further purification, unless otherwise stated. The 5-hydroxymethylfurfural oxidase variants (HMFO V465S, V465T, W466H and W466H/V465T) as well as wild type were employed as a purified enzyme solution, which was prepared as previously reported.[1] HMFO V367R/W466F was purchased form GECCO (Groningen, Netherlands). Variants of the berberine bridge enzyme analogue from *Arabidopsis thaliana* (*At*BBE-like15 L182V/L178V/I184V and L182V/I409V) were employed as a purified enzyme solution. Catalase from *Micrococcus lysodeikticus* (170000 U/mL) was purchased from *Sigma-Aldrich*.

1.2. Synthesis

1.2.1. Synthesis of allylic alcohols from their corresponding ketones

For procedure see main paper. The yields after the purification are reported in Table S1.

Table S1. Details of the reduction reactions

	**Starting allylic ketones**	**NaBH$_4$**	**Yield**
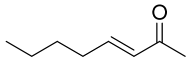 (*E*)-oct-3-en-2-one (**1b**)	MW: 126.20<br>2 g (16 mmol)	0.61 g (16 mmol)	2.10 g (quant.)
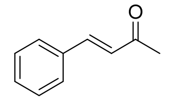 (*E*)-4-phenylbut-3-en-2-one (**2b**)	MW: 146.07<br>2 g (14 mmol)	0.53 g (13 mmol)	2.16 g (quant.)
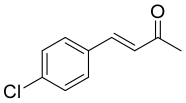 (*E*)-4-(4-chlorophenyl)but-3-en-2-one (**3b**)	MW: 180.03<br>2 g (11 mmol)	0.42 g (11 mmol)	2.13 g (quant.)
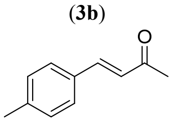 (*E*)-4-(4-methylphenyl)but-3-en-2-one (**4b**)	MW: 160.22<br>2 g (13 mmol)	0.47 g (13 mmol)	1.74 g (87%)

1.2.2. NMR of the allylic alcohols syanthesized

a) (*E*)-oct-3-en-2-ol (1a)

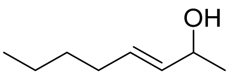

2.10 g (quant. yield, colorless oil), ^{1}H NMR (300 MHz, CDCl$_3$): δ 5.57 (1H, dt, J = 15.1, 6.4 Hz, CH$_2$C**H**=), 5.45 (1H, dd, J = 15.4, 6.3, HOCHC**H**=), 4.19 (1H, p, J = 6.3, OC**H**), 2.46 (1H, br s, OH), 1.97 (2H, q, J = 6.6 Hz, =CHC**H$_2$**), 1.31-1.28 (4H, m, CH$_3$C**H$_2$**C**H$_2$**), 1.19 (3H, d, J = 6.4 Hz,

OCHC**H**$_3$), 0.85 (3H, t, *J* = 7.1 Hz, C**H**$_3$CH$_2$).; ^{13}C NMR (75 MHz, CDCl$_3$) δ 134.2, 130.7, 68.7, 31.7, 31.3, 23.3, 22.1, 13.8.[2] For NMR of the starting material see literature.[3]

b) (*E*)-4-phenylbut-3-en-2-ol (2a)

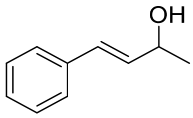

2.16 g (quant. yield, pale yellow solid, mp 32-34 ºC), ^{1}H NMR (300 MHz, CDCl$_3$): δ 77.52 – 7.21 (5H, m, C$_6$**H**$_5$), 6.60 (1H, dd, *J* = 16.0, 0.8 Hz, C$_6$H$_5$**H**C=), 6.30 (1H, dd, *J* = 15.9, 6.3 Hz, =C**H**CHOH), 4.51 (1H, pd, *J* = 6.4, 1.1 Hz, C**H**(OH)), 2.51 (1H, br s, O**H**), 1.41 (3H, d, *J* = 6.4 Hz, C**H**$_3$).; ^{13}C NMR (75 MHz, CDCl$_3$): δ 136.8, 133.7, 129.3, 128.6, 127.6, 126.5, 68.8, 23.5.[4] For NMR of starting material see literature.[5]

c) (*E*)-4-(4-chlorophenyl)but-3-en-2-ol (3a)

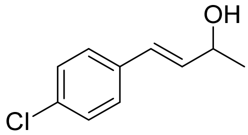

2.13 g (quant. yield, colorless solid, mp 57-60 ºC), ^{1}H NMR (300 MHz, CDCl$_3$): 7.40 – 7.23 (4H, m, Ar), 6.55 (1H, dd, *J* =15.9, 1.0 Hz, Ar**H**C=), 6.26 (1H, dd, *J* =15.9, 6.2 Hz, =C**H**CHOH), 4.61 – 4.40 (1H, m, C**H**(OH)), 1.62 (1H, s, OH), 1.39 (1H, d, *J* =6.4 Hz, C**H**$_3$).; ^{13}C NMR (75 MHz, CDCl$_3$) δ 135.2, 134.2, 133.2, 128.7, 128.1, 127.7, 68.8, 23.4.[6] For NMR of starting matrerial see literature.[7]

d) (*E*)-4-(4-methylphenyl)but-3-en-2-ol (4a)

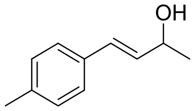

1.74 g (87% yield, pale yellow solid, mp 39-42 ºC), ^{1}H NMR (300 MHz, CDCl$_3$): δ 7.38 (2H, d, *J* = 8.1 Hz, Ar), 7.22 (2H, d, *J* = 8.0 Hz, Ar), 6.63 (1H, d, *J* =15.9 Hz, Ar**H**C=), 6.34 (1H, dd, *J* =15.9, 6.3 Hz, =C**H**CHOH), 4.58 (1H, p, *J* = 6.3 Hz, C**H**(OH)), 3.90 (1H, s, OH), 2.46 (3H, s, C**H**$_3$), 1.50 (3H, d, *J* =6.4 Hz, HOCHC**H**$_3$).; ^{13}C NMR (75 MHz, CDCl$_3$) δ 137.3, 134.3, 133.0, 129.4, 129.1, 126.6, 68.8, 23.6, 21.3.[6]

NMRs of commercial **5a**[8] and **5b**[9] can be found in literature.

1.3. Biocatalytic reactions

1.3.1. Preparation of the biocatalysts

*At*BBE-like15 L182V/I409V and *At*BBE-like15 L178V/L182V/I184V

Protein expression and purification: The expressions of AtBBE15 L182V was performed as described before.[10] *At*BBE-like15 L182V/I409V and *At*BBE-like15 L178V/L182V/I184V were expressed in shake flask using minimal media.[11] The compositions of every used media are listed in Table S2 and the components are listed in Table S3.

Table S2. Compositions of used media for expression of *At*BBE-like15 variants

BMD Media per L	BMM10 Media per 100 mL
200 mL 2M PPB (K_2HPO_4 and KH_2PO_4)	20 mL 2M PPB (K_2HPO_4 and KH_2PO_4)
100 mL 10x YNB	10 mL 10x YNB
50 mL 10x D-Glucose	5 mL MeOH abs.
2 mL 500x Biotin	0.2 mL 500x Biotin
650mL H_2O sterile	65mL H_2O

Table S3. Components of used media for expression of *At*BBE-like15 variants

**PPB buffer**	239.5 g/L KH_2PO_4, 41.8 g/L K_2HPO_4, pH 6	Autoclaved
**10x YNB**	134 g/L Yeast nitrogen base (Bacto)	Autoclaved
**10x D-Glucose**	200 g/L D-Glucose	Autoclaved
**500x Biotin**	0.2 mg/ml Biotin	Sterile filtered

i) AtBBE15 synthetic gene

Restriction sites NdeI, NotI

DNA sequence:

CTCACTATAGGGCGAATTGAGTGAAGGCCGTCAAGGCCTAGGCGCGCCACATATGGCGTT
TGCGATATCAAAGCGAAACGCAACCTTGTTTCTTGTAACGCTACTACTAATCTCCGTTCCA
TTGTCTTCCTCCACGCTACAACAAGATTTCGTGAAGTGCCTTGTCGACAACTCCGACGTCT
CCTTCCCCATAACGGCGTCGTTTTTCTCACCGGACCAAAACGCTACTTTGTTTAAAGAGGA
GCTTGAATCAACGGCACAGAATCTCCGTTACTTGACGCCGTCAAACCCGAAGCCCGTGTT
CATATTCGAGCCTTTGTACGAGACGCATGTCCAAGCAGCGGTCGTGTGTGCCAAGAAGCT
TCAGCTTCACCTCCGACTACGTAGCGGTGGTCACGACTACGAAGGGCTCTCGTTTGTTGCC
GAGGACGAAACGCCGTTTGTGATCGTTGATTTGTCGAAGCTTAGACAGGTTGACGTTGAT
TTGGACAGTAACAGTGCGTGGGCTCATGCTGGTGCTACCATCGGAGAGGTTTATTACAGG
ATCCAAGAGAAAAGCCAAACCCATGGTTTTCCGGCAGGTTTATGCTCAAGCCTTGGCATC
GGTGGCCACTTAGTCGGTGGAGCGTACGGTTCCATGATGAGGAAGTTCGGTCTCGGCGCT
GACAATGTCCTCGACGCCAGAATCGTCGACGCCAACGGCCAAATCCTCGATCGAGCGGC
AATGGGAGAAGACGTCTTCTGGGCGATTCGAGGCGGCGGCGGTGGTAGCTTCGGCGTGA
TATTGGCCTGGAAGATTAAGCTCGTCCCCGTTCCGGCGACCGTTACGGTATTCACAGTCA
CGAAGACGCTTGAGCAAGACGGAACTAAAGTTTTATACAAATGGGAACAAATCGCTGAT
AAGCTTGACGATGATCTCTTCATTCGAGTCATTATTTCACCGGCCAGTAAAACCACCAAA

CCGGGAAATCGAACCATCTCGATGTCGTACCAAGCTCAGTTTCTCGGAGACTCCAATCGA
CTCTTGCAGGTGATGCAGAAGAGTTTCCCTGAGTTAGGACTGACGAAGAAGGACTGCACA
GAAATGAGCTGGATCAAATCAGTGATGTACATTGCAGGTTTTCCAAACAGTGCAGCACCG
GAAGCTTTACTAGCCGGAAAATCATTGTTCAAGAATCACTTCAAAGCCAAGTCAGACTTT
GTGAAAGAGCCAATTCCAGTAGAAGGTTTAGAAGGATTATGGGAAAGGTTTCTAGAAGA
AGATTCACCGTTAACGATATGGAACCCTTACGGAGGAATGATGTCGAGGATCTCCGAGTC
AGAGATACCTTTCCCTCATAGGAACGGGACATTGTTCAAGATTCAGTGGCTAAGCACGTG
GCAAGATGGGAAAGTGAGCGAGGAAAGGCACATGAAGTGGATTAGGGAGATGTATAGTT
ACATGGAGCAGTATGTGTCGAAGAACCCGAGACAGGCCTATGTGAATTACAGGGATCTT
GATTTGGGGACTAATGAAGGAGAGACTGATGCTAGAGAGTGGGGTGCTAAGTATTACAA
AGGGAATTTCGAGAGGTTGGTGAAGATTAAGGGTGAGTTTGATCCTGATAATTTCTTCAG
GCATGAACAGAGTGTTCCCACAAAGATTGGTCGGCCGGAAAACCTGTATTTTCAGGGCCA
TCACCATCACCATCACCATCACTAAGCGGCCGCATTAATTAACTGGCCTCATGGGCCTTC
ACTTCACTGCCCGCTTTCCAGT

***At*BBE15 (excluding vector coded regions (alpha-factor))**

Amino acid sequence:

T L Q Q D F V K C L V D N S D V S F P I T A S F F S P D Q N A T L F K E E L E S T A Q N L R
Y L T P S N P K P V F I F E P L Y E T H V Q A A V V C A K K L Q L H L R L R S G G H D Y E G
L S F V A E D E T P F V I V D L S K L R Q V D V D L D S N S A W A H A G A T I G E V Y Y R I
Q E K S Q T H G F P A G L C S S L G I G G H L V G G A Y G S M M R K F G L G A D N V L D A
R I V D A N G Q I L D R A A M G E D V F W A I R G G G G G S F G V I L A W K I K L V P V P
A T V T V F T V T K T L E Q D G T K V L Y K W E Q I A D K L D D D L F I R V I I S P A S K T
T K P G N R T I S M S Y Q A Q F L G D S N R L L Q V M Q K S F P E L G L T K K D C T E M S
W I K S V M Y I A G F P N S A A P E A L L A G K S L F K N H F K A K S D F V K E P I P V E G
L E G L W E R F L E E D S P L T I W N P Y G G M M S R I S E S E I P F P H R N G T L F K I Q W
L S T W Q D G K V S E E R H M K W I R E M Y S Y M E Q Y V S K N P R Q A Y V N Y R D L D
L G T N E G E T D A R E W G A K Y Y K G N F E R L V K I K G E F D P D N F F R H E Q S V P
T K

***ii*) *At*BBE15 L182V**

DNA sequence:

CTCACTATAGGGCGAATTGAGTGAAGGCCGTCAAGGCCTAGGCGCGCCACATATGGCGTT
TGCGATATCAAAGCGAAACGCAACCTTGTTTCTTGTAACGCTACTACTAATCTCCGTTCCA
TTGTCTTCCTCCACGCTACAACAAGATTTCGTGAAGTGCCTTGTCGACAACTCCGACGTCT
CCTTCCCCATAACGGCGTCGTTTTTCTCACCGGACCAAAACGCTACTTTGTTTAAAGAGGA
GCTTGAATCAACGGCACAGAATCTCCGTTACTTGACGCCGTCAAACCCGAAGCCCGTGTT
CATATTCGAGCCTTTGTACGAGACGCATGTCCAAGCAGCGGTCGTGTGTGCCAAGAAGCT

TCAGCTTCACCTCCGACTACGTAGCGGTGGTCACGACTACGAAGGGCTCTCGTTTGTTGCC
GAGGACGAAACGCCGTTTGTGATCGTTGATTTGTCGAAGCTTAGACAGGTTGACGTTGAT
TTGGACAGTAACAGTGCGTGGGCTCATGCTGGTGCTACCATCGGAGAGGTTTATTACAGG
ATCCAAGAGAAAAGCCAAACCCATGGTTTTCCGGCAGGTTTATGCTCAAGCGTTGGCATC
GGTGGCCACTTAGTCGGTGGAGCGTACGGTTCCATGATGAGGAAGTTCGGTCTCGGCGCT
GACAATGTCCTCGACGCCAGAATCGTCGACGCCAACGGCCAAATCCTCGATCGAGCGGC
AATGGGAGAAGACGTCTTCTGGGCGATTCGAGGCGGCGGCGGTGGTAGCTTCGGCGTGA
TATTGGCCTGGAAGATTAAGCTCGTCCCCGTTCCGGCGACCGTTACGGTATTCACAGTCA
CGAAGACGCTTGAGCAAGACGGAACTAAAGTTTTATACAAATGGGAACAAATCGCTGAT
AAGCTTGACGATGATCTCTTCATTCGAGTCATTATTTCACCGGCCAGTAAAACCACCAAA
CCGGGAAATCGAACCATCTCGATGTCGTACCAAGCTCAGTTTCTCGGAGACTCCAATCGA
CTCTTGCAGGTGATGCAGAAGAGTTTCCCTGAGTTAGGACTGACGAAGAAGGACTGCACA
GAAATGAGCTGGATCAAATCAGTGATGTACATTGCAGGTTTTCCAAACAGTGCAGCACCG
GAAGCTTTACTAGCCGGAAAATCATTGTTCAAGAATCACTTCAAAGCCAAGTCAGACTTT
GTGAAAGAGCCAATTCCAGTAGAAGGTTTAGAAGGATTATGGGAAAGGTTTCTAGAAGA
AGATTCACCGTTAACGATATGGAACCCTTACGGAGGAATGATGTCGAGGATCTCCGAGTC
AGAGATACCTTTCCCTCATAGGAACGGGACATTGTTCAAGATTCAGTGGCTAAGCACGTG
GCAAGATGGGAAAGTGAGCGAGGAAAGGCACATGAAGTGGATTAGGGAGATGTATAGTT
ACATGGAGCAGTATGTGTCGAAGAACCCGAGACAGGCCTATGTGAATTACAGGGATCTT
GATTTGGGGACTAATGAAGGAGAGACTGATGCTAGAGAGTGGGGTGCTAAGTATTACAA
AGGGAATTTCGAGAGGTTGGTGAAGATTAAGGGTGAGTTTGATCCTGATAATTTCTTCAG
GCATGAACAGAGTGTTCCCACAAAGATTGGTCGGCCGGAAAACCTGTATTTTCAGGGCCA
TCACCATCACCATCACCATCACTAAGCGGCCGCATTAATTAACTGGCCTCATGGGCCTTC
ACTTCACTGCCCGCTTTCCAGT

***At*BBE15 L182V (excluding vector coded regions (alpha-factor, hexa-His-tag))**

Amino acid sequence:

T L Q Q D F V K C L V D N S D V S F P I T A S F F S P D Q N A T L F K E E L E S T A Q N L R
Y L T P S N P K P V F I F E P L Y E T H V Q A A V V C A K K L Q L H L R L R S G G H D Y E G
L S F V A E D E T P F V I V D L S K L R Q V D V D L D S N S A W A H A G A T I G E V Y Y R I
Q E K S Q T H G F P A G L C S S V G I G G H L V G G A Y G S M M R K F G L G A D N V L D A
R I V D A N G Q I L D R A A M G E D V F W A I R G G G G G S F G V I L A W K I K L V P V P
A T V T V F T V T K T L E Q D G T K V L Y K W E Q I A D K L D D D L F I R V I I S P A S K T
T K P G N R T I S M S Y Q A Q F L G D S N R L L Q V M Q K S F P E L G L T K K D C T E M S
W I K S V M Y I A G F P N S A A P E A L L A G K S L F K N H F K A K S D F V K E P I P V E G
L E G L W E R F L E E D S P L T I W N P Y G G M M S R I S E S E I P F P H R N G T L F K I Q W
L S T W Q D G K V S E E R H M K W I R E M Y S Y M E Q Y V S K N P R Q A Y V N Y R D L D

L G T N E G E T D A R E W G A K Y Y K G N F E R L V K I K G E F D P D N F F R H E Q S V P T K I G R P E N L Y F Q G H H H H H H H H

***iii*) *At*BBE15 L182V/I409V**

DNA sequence:

CTCACTATAGGGCGAATTGAGTGAAGGCCGTCAAGGCCTAGGCGCGCCACATATGGCGTT
TGCGATATCAAAGCGAAACGCAACCTTGTTTCTTGTAACGCTACTACTAATCTCCGTTCCA
TTGTCTTCCTCCACGCTACAACAAGATTTCGTGAAGTGCCTTGTCGACAACTCCGACGTCT
CCTTCCCCATAACGGCGTCGTTTTTCTCACCGGACCAAAACGCTACTTTGTTTAAAGAGGA
GCTTGAATCAACGGCACAGAATCTCCGTTACTTGACGCCGTCAAACCCGAAGCCCGTGTT
CATATTCGAGCCTTTGTACGAGACGCATGTCCAAGCAGCGGTCGTGTGTGCCAAGAAGCT
TCAGCTTCACCTCCGACTACGTAGCGGTGGTCACGACTACGAAGGGCTCTCGTTTGTTGCC
GAGGACGAAACGCCGTTTGTGATCGTTGATTTGTCGAAGCTTAGACAGGTTGACGTTGAT
TTGGACAGTAACAGTGCGTGGGCTCATGCTGGTGCTACCATCGGAGAGGTTTATTACAGG
ATCCAAGAGAAAAGCCAAACCCATGGTTTTCCGGCAGGTTTATGCTCAAGCGTTGGCATC
GGTGGCCACTTAGTCGGTGGAGCGTACGGTTCCATGATGAGGAAGTTCGGTCTCGGCGCT
GACAATGTCCTCGACGCCAGAATCGTCGACGCCAACGGCCAAATCCTCGATCGAGCGGC
AATGGGAGAAGACGTCTTCTGGGCGATTCGAGGCGGCGGCGGTGGTAGCTTCGGCGTGA
TATTGGCCTGGAAGATTAAGCTCGTCCCCGTTCCGGCGACCGTTACGGTATTCACAGTCA
CGAAGACGCTTGAGCAAGACGGAACTAAAGTTTTATACAAATGGGAACAAATCGCTGAT
AAGCTTGACGATGATCTCTTCATTCGAGTCATTATTTCACCGGCCAGTAAAACCACCAAA
CCGGGAAATCGAACCATCTCGATGTCGTACCAAGCTCAGTTTCTCGGAGACTCCAATCGA
CTCTTGCAGGTGATGCAGAAGAGTTTCCCTGAGTTAGGACTGACGAAGAAGGACTGCACA
GAAATGAGCTGGATCAAATCAGTGATGTACATTGCAGGTTTTCCAAACAGTGCAGCACCG
GAAGCTTTACTAGCCGGAAAATCATTGTTCAAGAATCACTTCAAAGCCAAGTCAGACTTT
GTGAAAGAGCCAATTCCAGTAGAAGGTTTAGAAGGATTATGGGAAAGGTTTCTAGAAGA
AGATTCACCGTTAACGATTTGGAACCCTTACGGAGGAATGATGTCGAGGATCTCCGAGTC
AGAGATACCTTTCCCTCATAGGAACGGGACATTGTTCAAGATTCAGTGGCTAAGCACGTG
GCAAGATGGGAAAGTGAGCGAGGAAAGGCACATGAAGTGGATTAGGGAGATGTATAGTT
ACATGGAGCAGTATGTGTCGAAGAACCCGAGACAGGCCTATGTGAATTACAGGGATCTT
GATTTGGGGACTAATGAAGGAGAGACTGATGCTAGAGAGTGGGGTGCTAAGTATTACAA
AGGGAATTTCGAGAGGTTGGTGAAGATTAAGGGTGAGTTTGATCCTGATAATTTCTTCAG
GCATGAACAGAGTGTTCCCACAAAGATTGGTCGGCCGGAAAACCTGTATTTTCAGGGCCA
TCACCATCACCATCACCATCACTAAGCGGCCGCATTAATTAACTGGCCTCATGGGCCTTC
ACTTCACTGCCCGCTTTCCAGT

***At*BBE15 L182V/I409V (excluding vector coded regions (alpha-factor, hexa-His-tag))**

Amino acid sequence:

T L Q Q D F V K C L V D N S D V S F P I T A S F F S P D Q N A T L F K E E L E S T A Q N L R
Y L T P S N P K P V F I F E P L Y E T H V Q A A V V C A K K L Q L H L R L R S G G H D Y E G
L S F V A E D E T P F V I V D L S K L R Q V D V D L D S N S A W A H A G A T I G E V Y Y R I
Q E K S Q T H G F P A G L C S S V G I G G H L V G G A Y G S M M R K F G L G A D N V L D A
R I V D A N G Q I L D R A A M G E D V F W A I R G G G G G S F G V I L A W K I K L V P V P
A T V T V F T V T K T L E Q D G T K V L Y K W E Q I A D K L D D D L F I R V I I S P A S K T
T K P G N R T I S M S Y Q A Q F L G D S N R L L Q V M Q K S F P E L G L T K K D C T E M S
W I K S V M Y I A G F P N S A A P E A L L A G K S L F K N H F K A K S D F V K E P I P V E G
L E G L W E R F L E E D S P L T V W N P Y G G M M S R I S E S E I P F P H R N G T L F K I Q
W L S T W Q D G K V S E E R H M K W I R E M Y S Y M E Q Y V S K N P R Q A Y V N Y R D L
D L G T N E G E T D A R E W G A K Y Y K G N F E R L V K I K G E F D P D N F F R H E Q S V
P T K

***iv) At*BBE15 L182V/L184V/L178V synthetic gene**

Restriction sites Xho1, NotI

DNA sequence:

ATGCTCGAGAAGAGAGAAGCTGAGGCTACCTTGCAACAGGACTTCGTTAAGTGTTTGGTC
GACAACTCCGACGTGTCTTTCCCAATCACTGCCTCATTCTTCTCACCAGACCAGAACGCCA
CTCTGTTCAAAGAAGAATTGGAGTCCACCGCTCAGAACCTGAGATACTTGACTCCATCTA
ACCCAAAGCCAGTGTTCATCTTCGAACCACTGTACGAGACTCACGTTCAGGCTGCTGTTG
TTTGTGCTAAGAAGTTGCAGTTGCACCTGAGATTGAGATCTGGTGGTCACGACTACGAAG
GTTTGTCTTTTGTTGCTGAGGACGAGACTCCATTCGTCATCGTTGACTTGTCCAAGTTGAG
ACAGGTTGACGTCGACTTGGACTCTAACTCTGCTTGGGCTCATGCTGGTGCTACTATTGGT
GAGGTCTACTACAGAATCCAAGAGAAGTCCCAGACTCACGGTTTTCCAGCTGGTGTTTGT
TCTTCCGTTGGTGTTGGTGGTCATTTGGTTGGTGGTGCTTACGGTTCCATGATGAGAAAGT
TTGGTTTGGGTGCCGACAACGTTTTGGACGCTAGAATCGTTGATGCCAACGGTCAGATTTT
GGACAGAGCTGCTATGGGTGAAGATGTGTTCTGGGCTATTAGAGGTGGTGGTGGCGGTTC
TTTCGGTGTTATTTTGGCTTGGAAGATCAAGCTGGTTCCAGTTCCAGCTACCGTTACCGTT
TTCACTGTTACCAAGACTTTGGAGCAGGACGGTACTAAGGTTCTGTACAAGTGGGAGCAG
ATCGCTGATAAGTTGGACGACGACTTGTTCATCAGAGTCATCATTTCCCCAGCTTCCAAG
ACTACCAAGCCAGGTAACAGAACTATCTCCATGTCCTACCAGGCTCAGTTCTTGGGTGAT
TCCAACAGATTGCTGCAGGTCATGCAGAAGTCCTTTCCAGAGTTGGGTCTGACCAAGAAA
GACTGTACTGAGATGTCCTGGATCAAGTCCGTCATGTACATTGCTGGTTTCCCAAACTCTG
CTGCTCCAGAAGCTTTGTTGGCTGGTAAGTCCTTGTTCAAGAACCACTTCAAGGCCAAGT
CCGACTTCGTCAAAGAGCCAATTCCAGTCGAAGGTCTGGAAGGATTGTGGGAGAGATTCT

TGGAAGAGGACTCCCCATTGACTATCTGGAACCCATACGGTGGTATGATGTCCAGAATTT
CCGAGTCCGAGATTCCATTTCCACACAGAAACGGAACCCTGTTCAAGATCCAATGGTTGT
CCACTTGGCAAGACGGTAAGGTGTCTGAAGAAAGACACATGAAGTGGATCCGTGAGATG
TACTCCTACATGGAACAATACGTGTCTAAGAACCCAAGACAGGCCTACGTCAACTACAGA
GATTTGGACTTGGGAACTAACGAGGGTGAGACTGATGCTAGAGAATGGGGTGCTAAGTA
CTACAAGGGTAACTTCGAGAGACTGGTCAAGATCAAGGGTGAATTCGACCCAGACAACT
TCTTTAGACACGAGCAGTCCGTTCCTACCAAGATTGGTGCGGCCGC

***At*BBE15 L182V/L184V/L178V (excluding vector coded regions (alpha-factor, hexa-His-tag))**

Amino acid sequence:
T L Q Q D F V K C L V D N S D V S F P I T A S F F S P D Q N A T L F K E E L E S T A Q N L R
Y L T P S N P K P V F I F E P L Y E T H V Q A A V V C A K K L Q L H L R L R S G G H D Y E G
L S F V A E D E T P F V I V D L S K L R Q V D V D L D S N S A W A H A G A T I G E V Y Y R I
Q E K S Q T H G F P A G V C S S V G V G G H L V G G A Y G S M M R K F G L G A D N V L D
A R I V D A N G Q I L D R A A M G E D V F W A I R G G G G G S F G V I L A W K I K L V P V
P A T V T V F T V T K T L E Q D G T K V L Y K W E Q I A D K L D D D L F I R V I I S P A S K
T T K P G N R T I S M S Y Q A Q F L G D S N R L L Q V M Q K S F P E L G L T K K D C T E M
S W I K S V M Y I A G F P N S A A P E A L L A G K S L F K N H F K A K S D F V K E P I P V E
G L E G L W E R F L E E D S P L T I W N P Y G G M M S R I S E S E I P F P H R N G T L F K I Q
W L S T W Q D G K V S E E R H M K W I R E M Y S Y M E Q Y V S K N P R Q A Y V N Y R D L
D L G T N E G E T D A R E W G A K Y Y K G N F E R L V K I K G E F D P D N F F R H E Q S V
P T K

HMFO wt (pEG 387), HMFO V465S (pEG 392), HMFO V465T (pEG 393), HMFO W466H (pEG 390) and HMFO V465T/W466H (pEG 395)

For the different variants of HMFO, the same expression and purification method was used as mentioned in the main manuscript.
His-Tagged HMFO was purified by immobilized Ni-affinity chromatography (5 mL HisTrap FF column, GE Healthcare) applying a 5 to 500 mM gradient of imidazole. Collected fractions were analyzed by SDS-PAGE (see Figure S1). Fractions containing HMFO were pooled, concentrated by ultrafiltration and desalted.

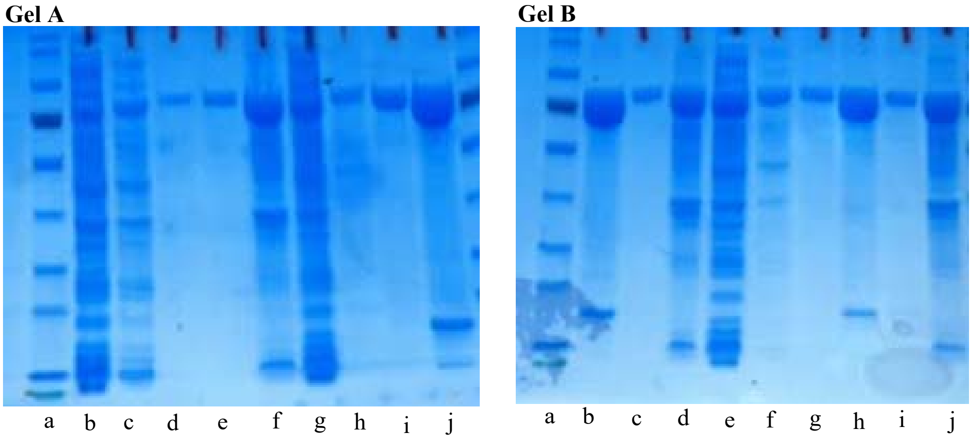

Figure S1. **Gel A**: a) page ruler, b) HMFO V465T flow through, c) HMFO V465T washing fraction, d) HMFO V465T, elution fraction 1, e) HMFO V465T elution fraction 2, f) HMFO V465T cell pellets, g) HMFO V465S flow through, h) HMFO V465S washing fraction, i) HMFO V465S, elution fraction 1, j) HMFO V465S, elution fraction 2.
Gel B: a) page ruler, b) HMFO V465S, elution fraction 2, c) HMFO V465S pure, d) HMFO V465S cell pellets, e) HMFO V465T/ W466H flow through, f) HMFO V465T/ W466H washing fraction, g) HMFO V465T/ W466H elution fraction 1, h) HMFO V465T/ W466H elution fraction 2, i) HMFO V465T/ W466H pure, j) HMFO V465T/ W466H cell pellets.

i) HMFO V465S (pEG 392)

Amino acid sequence:

MTDTIFDYVIVGGGTAGSVLANRLSARPENRVLLIEAGIDTPENNIPPEIHDGLRPWLPRLSGD
KFFWPNLTIHRAAEHPGITREPQFYEQGRLLGGGSSVNMVVSNRGLPRDYDEWQALGADGW
DWQGVLPYFIKTERDADYGDDPLHGNAGPIPIGRVDSRHWSDFTVAATQALEAAGLPNIHDQ
NARFDDGYFPPAFTLKGEERFSAARGYLDASVRVRPNLSLWTESRVLKLLTTGNAITGVSVLR
GRETLQVQAREVILTAGALQSPAILLRTGIGPAADLHALGIPVLADRPGVGRNLWEHSSIGVV
APLTEQARADASTGKAGSRHQLGIRASSGVDPATPSDLFLHIGADPVSGLASAVFWVNKPSST
GWLKLKDADPFSYPDVDFNLLSDPRDLGRLKAGLRLITHYFAAPSLAKYGLALALSRFAAPQ
PGGPLLNDLLQDEAALERYLRTNVGGSWHASGTARIGRADDSQAVVDKAGRVYGVTGLRV
ADASIMPTVPTANTNLPTLMLAEKIADAILTQA

DNA sequence:

ATGACTGATACGATTTTTGACTACGTGATTGTTGGCGGTGGCACGGCGGGTAGCGTTCTG
GCCAACCGTCTGTCCGCCCGTCCGGAGAATCGCGTGTTGCTGATTGAGGCCGGTATTGAT
ACCCCGGAAAACAATATTCCGCCGGAGATCCACGATGGCCTGCGCCCGTGGCTGCCGCGT
CTGAGCGGTGATAAGTTCTTTTGGCCGAATCTGACCATCCACCGTGCCGCGGAACACCCG
GGTATCACGCGCGAGCCGCAGTTCTATGAACAAGGCCGTCTGCTGGGCGGTGGTAGCAGC
GTGAACATGGTCGTTTCTAACCGTGGTCTGCCTCGCGACTATGACGAATGGCAGGCACTG
GGCGCAGATGGTTGGGATTGGCAGGGTGTTCTGCCGTACTTCATCAAGACCGAGCGTGAC
GCGGACTACGGTGACGACCCGTTGCATGGCAATGCGGGTCCGATTCCGATCGGTCGCGTC
GATTCGCGTCACTGGAGCGACTTCACGGTGGCGGCAACCCAAGCTCTGGAAGCGGCTGGC
CTGCCGAACATTCACGACCAAAACGCACGTTTTGATGACGGTTACTTCCCACCGGCATTT

ACGTTGAAAGGTGAAGAGCGCTTCAGCGCCGCACGCGGTTATCTGGATGCGAGCGTCCGT
GTGCGTCCGAACCTGAGCCTGTGGACTGAGAGCCGTGTCCTGAAGCTGCTGACCACTGGC
AATGCAATCACCGGTGTGAGCGTGCTGCGTGGTCGCGAAACCCTGCAAGTTCAAGCGCGC
GAGGTCATCCTGACCGCCGGTGCGTTGCAAAGCCCAGCGATTCTGTTGCGCACCGGCATC
GGCCCTGCGGCGGATCTGCACGCACTGGGTATTCCTGTTCTGGCAGACCGTCCGGGTGTT
GGTCGCAATCTGTGGGAGCACAGCTCTATCGGTGTGGTTGCCCCGCTGACCGAGCAGGCA
CGTGCAGACGCCAGCACGGGTAAAGCCGGCTCTCGCCATCAACTGGGTATCCGTGCGTCG
TCCGGCGTAGATCCGGCGACGCCTAGCGACCTGTTTCTGCATATCGGTGCTGATCCAGTC
AGCGGTCTGGCAAGCGCTGTGTTCTGGGTGAACAAGCCAAGCTCCACCGGCTGGCTGAAG
CTGAAGGACGCGGACCCGTTTAGCTACCCGGACGTAGACTTCAATCTGCTGAGCGATCCG
CGCGACTTGGGTCGTCTGAAAGCGGGCCTGCGTCTGATCACCCATTACTTCGCAGCGCCG
TCCCTGGCGAAATATGGTTTGGCGCTGGCATTGAGCCGTTTTGCGGCACCGCAGCCGGGT
GGTCCGCTGCTGAACGACCTGTTGCAGGACGAAGCCGCCCTGGAACGCTATTTGCGTACG
AACGTCGGCGGTAGCTGGCATGCGAGCGGCACGGCGCGTATCGGCCGTGCGGATGATTC
CCAGGCTGTTGTCGATAAAGCGGGTCGTGTGTACGGCGTCACCGGCCTGCGTGTTGCGGA
CGCAAGCATTATGCCGACCGTTCCGACCGCCAATACCAATCTGCCGACGCTGATGCTGGC
TGAGAAAATTGCGGATGCGATTCTGACCCAGGCTCTGCAGGAGAATTTATATTTTCAAGG
TGCCGGCTGGAGCCACCCGCAGTTTGAAAAATAA

***ii)* HMFO V465T (pEG 393)**

Amino acid sequence:

MTDTIFDYVIVGGGTAGSVLANRLSARPENRVLLIEAGIDTPENNIPPEIHDGLRPWLPRLSGD
KFFWPNLTIHRAAEHPGITREPQFYEQGRLLGGGSSVNMVVSNRGLPRDYDEWQALGADGW
DWQGVLPYFIKTERDADYGDDPLHGNAGPIPIGRVDSRHWSDFTVAATQALEAAGLPNIHDQ
NARFDDGYFPPAFTLKGEERFSAARGYLDASVRVRPNLSLWTESRVLKLLTTGNAITGVSVLR
GRETLQVQAREVILTAGALQSPAILLRTGIGPAADLHALGIPVLADRPGVGRNLWEHSSIGVV
APLTEQARADASTGKAGSRHQLGIRASSGVDPATPSDLFLHIGADPVSGLASAVFWVNKPSST
GWLKLKDADPFSYPDVDFNLLSDPRDLGRLKAGLRLITHYFAAPSLAKYGLALALSRFAAPQ
PGGPLLNDLLQDEAALERYLRTNVGGTWHASGTARIGRADDSQAVVDKAGRVYGVTGLRV
ADASIMPTVPTANTNLPTLMLAEKIADAILTQA

DNA sequence:

ATGACTGATACGATTTTTGACTACGTGATTGTTGGCGGTGGCACGGCGGGTAGCGTTCTG
GCCAACCGTCTGTCCGCCCGTCCGGAGAATCGCGTGTTGCTGATTGAGGCCGGTATTGAT
ACCCCGGAAAACAATATTCCGCCGGAGATCCACGATGGCCTGCGCCCGTGGCTGCCGCGT
CTGAGCGGTGATAAGTTCTTTTGGCCGAATCTGACCATCCACCGTGCCGCGGAACACCCG
GGTATCACGCGCGAGCCGCAGTTCTATGAACAAGGCCGTCTGCTGGGCGGTGGTAGCAGC
GTGAACATGGTCGTTTCTAACCGTGGTCTGCCTCGCGACTATGACGAATGGCAGGCACTG

GGCGCAGATGGTTGGGATTGGCAGGGTGTTCTGCCGTACTTCATCAAGACCGAGCGTGAC
GCGGACTACGGTGACGACCCGTTGCATGGCAATGCGGGTCCGATTCCGATCGGTCGCGTC
GATTCGCGTCACTGGAGCGACTTCACGGTGGCGGCAACCCAAGCTCTGGAAGCGGCTGGC
CTGCCGAACATTCACGACCAAAACGCACGTTTTGATGACGGTTACTTCCCACCGGCATTT
ACGTTGAAAGGTGAAGAGCGCTTCAGCGCCGCACGCGGTTATCTGGATGCGAGCGTCCGT
GTGCGTCCGAACCTGAGCCTGTGGACTGAGAGCCGTGTCCTGAAGCTGCTGACCACTGGC
AATGCAATCACCGGTGTGAGCGTGCTGCGTGGTCGCGAAACCCTGCAAGTTCAAGCGCGC
GAGGTCATCCTGACCGCCGGTGCGTTGCAAAGCCCAGCGATTCTGTTGCGCACCGGCATC
GGCCCTGCGGCGGATCTGCACGCACTGGGTATTCCTGTTCTGGCAGACCGTCCGGGTGTT
GGTCGCAATCTGTGGGAGCACAGCTCTATCGGTGTGGTTGCCCCGCTGACCGAGCAGGCA
CGTGCAGACGCCAGCACGGGTAAAGCCGGCTCTCGCCATCAACTGGGTATCCGTGCGTCG
TCCGGCGTAGATCCGGCGACGCCTAGCGACCTGTTTCTGCATATCGGTGCTGATCCAGTC
AGCGGTCTGGCAAGCGCTGTGTTCTGGGTGAACAAGCCAAGCTCCACCGGCTGGCTGAAG
CTGAAGGACGCGGACCCGTTTAGCTACCCGGACGTAGACTTCAATCTGCTGAGCGATCCG
CGCGACTTGGGTCGTCTGAAAGCGGGCCTGCGTCTGATCACCCATTACTTCGCAGCGCCG
TCCCTGGCGAAATATGGTTTGGCGCTGGCATTGAGCCGTTTTGCGGCACCGCAGCCGGGT
GGTCCGCTGCTGAACGACCTGTTGCAGGACGAAGCCGCCCTGGAACGCTATTTGCGTACG
AACGTCGGCGGTACCTGGCATGCGAGCGGCACGGCGCGTATCGGCCGTGCGGATGATTCC
CAGGCTGTTGTCGATAAAGCGGGTCGTGTGTACGGCGTCACCGGCCTGCGTGTTGCGGAC
GCAAGCATTATGCCGACCGTTCCGACCGCCAATACCAATCTGCCGACGCTGATGCTGGCT
GAGAAAATTGCGGATGCGATTCTGACCCAGGCTCTGCAGGAGAATTTATATTTTCAAGGT
GCCGGCTGGAGCCACCCGCAGTTTGAAAAATAA

iii) **HMFO W466H (pEG 390)**

Amino acid sequence:
MTDTIFDYVIVGGGTAGSVLANRLSARPENRVLLIEAGIDTPENNIPPEIHDGLRPWLPRLSGD
KFFWPNLTIHRAAEHPGITREPQFYEQGRLLGGGSSVNMVVSNRGLPRDYDEWQALGADGW
DWQGVLPYFIKTERDADYGDDPLHGNAGPIPIGRVDSRHWSDFTVAATQALEAAGLPNIHDQ
NARFDDGYFPPAFTLKGEERFSAARGYLDASVRVRPNLSLWTESRVLKLLTTGNAITGVSVLR
GRETLQVQAREVILTAGALQSPAILLRTGIGPAADLHALGIPVLADRPGVGRNLWEHSSIGVV
APLTEQARADASTGKAGSRHQLGIRASSGVDPATPSDLFLHIGADPVSGLASAVFWVNKPSST
GWLKLKDADPFSYPDVDFNLLSDPRDLGRLKAGLRLITHYFAAPSLAKYGLALALSRFAAPQ
PGGPLLNDLLQDEAALERYLRTNVGGVHHASGTARIGRADDSQAVVDKAGRVYGVTGLRV
ADASIMPTVPTANTNLPTLMLAEKIADAILTQA

DNA sequence:
ATGACTGATACGATTTTTGACTACGTGATTGTTGGCGGTGGCACGGCGGGTAGCGTTCTG
GCCAACCGTCTGTCCGCCCGTCCGGAGAATCGCGTGTTGCTGATTGAGGCCGGTATTGAT
ACCCCGGAAAACAATATTCCGCCGGAGATCCACGATGGCCTGCGCCCGTGGCTGCCGCGT

CTGAGCGGTGATAAGTTCTTTTGGCCGAATCTGACCATCCACCGTGCCGCGGAACACCCG
GGTATCACGCGCGAGCCGCAGTTCTATGAACAAGGCCGTCTGCTGGGCGGTGGTAGCAGC
GTGAACATGGTCGTTTCTAACCGTGGTCTGCCTCGCGACTATGACGAATGGCAGGCACTG
GGCGCAGATGGTTGGGATTGGCAGGGTGTTCTGCCGTACTTCATCAAGACCGAGCGTGAC
GCGGACTACGGTGACGACCCGTTGCATGGCAATGCGGGTCCGATTCCGATCGGTCGCGTC
GATTCGCGTCACTGGAGCGACTTCACGGTGGCGGCAACCCAAGCTCTGGAAGCGGCTGGC
CTGCCGAACATTCACGACCAAAACGCACGTTTTGATGACGGTTACTTCCCACCGGCATTT
ACGTTGAAAGGTGAAGAGCGCTTCAGCGCCGCACGCGGTTATCTGGATGCGAGCGTCCGT
GTGCGTCCGAACCTGAGCCTGTGGACTGAGAGCCGTGTCCTGAAGCTGCTGACCACTGGC
AATGCAATCACCGGTGTGAGCGTGCTGCGTGGTCGCGAAACCCTGCAAGTTCAAGCGCGC
GAGGTCATCCTGACCGCCGGTGCGTTGCAAAGCCCAGCGATTCTGTTGCGCACCGGCATC
GGCCCTGCGGCGGATCTGCACGCACTGGGTATTCCTGTTCTGGCAGACCGTCCGGGTGTT
GGTCGCAATCTGTGGGAGCACAGCTCTATCGGTGTGGTTGCCCCGCTGACCGAGCAGGCA
CGTGCAGACGCCAGCACGGGTAAAGCCGGCTCTCGCCATCAACTGGGTATCCGTGCGTCG
TCCGGCGTAGATCCGGCGACGCCTAGCGACCTGTTTCTGCATATCGGTGCTGATCCAGTC
AGCGGTCTGGCAAGCGCTGTGTTCTGGGTGAACAAGCCAAGCTCCACCGGCTGGCTGAAG
CTGAAGGACGCGGACCCGTTTAGCTACCCGGACGTAGACTTCAATCTGCTGAGCGATCCG
CGCGACTTGGGTCGTCTGAAAGCGGGCCTGCGTCTGATCACCCATTACTTCGCAGCGCCG
TCCCTGGCGAAATATGGTTTGGCGCTGGCATTGAGCCGTTTTGCGGCACCGCAGCCGGGT
GGTCCGCTGCTGAACGACCTGTTGCAGGACGAAGCCGCCCTGGAACGCTATTTGCGTACG
AACGTCGGCGGTGTTCATCATGCGAGCGGCACGGCGCGTATCGGCCGTGCGGATGATTCC
CAGGCTGTTGTCGATAAAGCGGGTCGTGTGTACGGCGTCACCGGCCTGCGTGTTGCGGAC
GCAAGCATTATGCCGACCGTTCCGACCGCCAATACCAATCTGCCGACGCTGATGCTGGCT
GAGAAAATTGCGGATGCGATTCTGACCCAGGCTCTGCAGGAGAATTTATATTTTCAAGGT
GCCGGCTGGAGCCACCCGCAGTTTGAAAAATAA

***iv)* HMFO V465T/W466H (pEG 395)**

Amino acid sequence:

MTDTIFDYVIVGGGTAGSVLANRLSARPENRVLLIEAGIDTPENNIPPEIHDGLRPWLPRLSGD
KFFWPNLTIHRAAEHPGITREPQFYEQGRLLGGGSSVNMVVSNRGLPRDYDEWQALGADGW
DWQGVLPYFIKTERDADYGDDPLHGNAGPIPIGRVDSRHWSDFTVAATQALEAAGLPNIHDQ
NARFDDGYFPPAFTLKGEERFSAARGYLDASVRVRPNLSLWTESRVLKLLTTGNAITGVSVLR
GRETLQVQAREVILTAGALQSPAILLRTGIGPAADLHALGIPVLADRPGVGRNLWEHSSIGVV
APLTEQARADASTGKAGSRHQLGIRASSGVDPATPSDLFLHIGADPVSGLASAVFWVNKPSST
GWLKLKDADPFSYPDVDFNLLSDPRDLGRLKAGLRLITHYFAAPSLAKYGLALALSRFAAPQ
PGGPLLNDLLQDEAALERYLRTNVGGTHHASGTARIGRADDSQAVVDKAGRVYGVTGLRVA
DASIMPTVPTANTNLPTLMLAEKIADAILTQA

DNA sequence:
ATGACTGATACGATTTTGACTACGTGATTGTTGGCGGTGGCACGGCGGGTAGCGTTCTG
GCCAACCGTCTGTCCGCCCGTCCGGAGAATCGCGTGTTGCTGATTGAGGCCGGTATTGAT
ACCCCGGAAAACAATATTCCGCCGGAGATCCACGATGGCCTGCGCCCGTGGCTGCCGCGT
CTGAGCGGTGATAAGTTCTTTGGCCGAATCTGACCATCCACCGTGCCGCGGAACACCCG
GGTATCACGCGCGAGCCGCAGTTCTATGAACAAGGCCGTCTGCTGGGCGGTGGTAGCAGC
GTGAACATGGTCGTTTCTAACCGTGGTCTGCCTCGCGACTATGACGAATGGCAGGCACTG
GGCGCAGATGGTTGGGATTGGCAGGGTGTTCTGCCGTACTTCATCAAGACCGAGCGTGAC
GCGGACTACGGTGACGACCCGTTGCATGGCAATGCGGGTCCGATTCCGATCGGTCGCGTC
GATTCGCGTCACTGGAGCGACTTCACGGTGGCGGCAACCCAAGCTCTGGAAGCGGCTGGC
CTGCCGAACATTCACGACCAAAACGCACGTTTTGATGACGGTTACTTCCCACCGGCATTT
ACGTTGAAAGGTGAAGAGCGCTTCAGCGCCGCACGCGGTTATCTGGATGCGAGCGTCCGT
GTGCGTCCGAACCTGAGCCTGTGGACTGAGAGCCGTGTCCTGAAGCTGCTGACCACTGGC
AATGCAATCACCGGTGTGAGCGTGCTGCGTGGTCGCGAAACCCTGCAAGTTCAAGCGCGC
GAGGTCATCCTGACCGCCGGTGCGTTGCAAAGCCCAGCGATTCTGTTGCGCACCGGCATC
GGCCCTGCGGCGGATCTGCACGCACTGGGTATTCCTGTTCTGGCAGACCGTCCGGGTGTT
GGTCGCAATCTGTGGGAGCACAGCTCTATCGGTGTGGTTGCCCCGCTGACCGAGCAGGCA
CGTGCAGACGCCAGCACGGGTAAAGCCGGCTCTCGCCATCAACTGGGTATCCGTGCGTCG
TCCGGCGTAGATCCGGCGACGCCTAGCGACCTGTTTCTGCATATCGGTGCTGATCCAGTC
AGCGGTCTGGCAAGCGCTGTGTTCTGGGTGAACAAGCCAAGCTCCACCGGCTGGCTGAAG
CTGAAGGACGCGGACCCGTTTAGCTACCCGGACGTAGACTTCAATCTGCTGAGCGATCCG
CGCGACTTGGGTCGTCTGAAAGCGGGCCTGCGTCTGATCACCCATTACTTCGCAGCGCCG
TCCCTGGCGAAATATGGTTTGGCGCTGGCATTGAGCCGTTTTGCGGCACCGCAGCCGGGT
GGTCCGCTGCTGAACGACCTGTTGCAGGACGAAGCCGCCCTGGAACGCTATTTGCGTACG
AACGTCGGCGGTAGCCATCATGCGAGCGGCACGGCGCGTATCGGCCGTGCGGATGATTCC
CAGGCTGTTGTCGATAAAGCGGGTCGTGTGTACGGCGTCACCGGCCTGCGTGTTGCGGAC
GCAAGCATTATGCCGACCGTTCCGACCGCCAATACCAATCTGCCGACGCTGATGCTGGCT
GAGAAAATTGCGGATGCGATTCTGACCCAGGCTCTGCAGGAGAATTTATATTTTCAAGGT
GCCGGCTGGAGCCACCCGCAGTTTGAAAAATAA

2. Results

2.1. Biotransformations

2.1.1. Cosolvent study with substrate 4a by using HMFO variants (1.4 µM)

Substrate **4a** was chosen for the cosolvent study by using four different variants of HMFO including V465S, V465T, W466H and V465T/W466H. DMSO, isooctane, *n*-heptane and glycerol were chosen as cosolvents and different ratio starting from 5% v/v to 50% v/v of these cosolvents was tested in the oxidation reaction.

i) HMFO V465S

Results from testing V465S, revealed that better conversion levels were achieved by using glycerol as cosolvent (Table S4, Figure S2). By using 20% v/v of glycerol 29% conversion (entry 3) was observed but by increasing the cosolvent ratio to 50%, conversion was dropped to 16% (entry 5). In case of water immiscible cosolvent no big different in term of conversion levels in the presence of different ratio of cosolvent was observed.

Table S4. Cosolvent study with substrate **4a** by using HMFO V465S [a]

**Entry**	**Cosolvent** / **v/v % of cosolvent**	**DMSO**	**Glycerol**	**Isooctane**	***n*-Heptane**
1	5	5	21	5	6
2	10	3	26	5	5
3	20	3	29	6	4
4	30	n.d.[a]	25	5	3
5	50	n.d.	16	5	2

[a] Condition: KPi (200 mM, pH 7.0) containing the oxidases (1.4 µM µg/mL final concentration in 1 mL reaction volume), catalase from *Micrococcus lysodeikticus* (30 µL, 170000 U/mL), the substrate (50 mM), 5% to 50% v/v of different cosolvents. The reaction mixtures and blanks were shaken 16 hours (170 rpm, 21 ºC) and extracted with ethyl acetate (2 x 500 µL), dried with Na_2SO_4 and measured with GC-MS (method: lowboilers) afterwards. Conversions were measured based on area ratio of ketone to substrate.
GC-MS Method: Injector temperature: 250 °C; Injection volume: 1 µL; Flow rate: 0.7 mL/min; Temperature program (low boilers method): 40 °C, hold time 2.0 min, 10 °C/min to 180 °C, hold time 1.0 min; EI mode, energy 70 eV, MS Source: 230 °C, MS Quadrupole: 150 °C. HP-5MS column (5% phenylmethylsiloxane, 30 m x 0.20 mm x 0.25 µm, J&W Scientific, Agilent Technologies) using He as carrier gas.
[b] No conversion was detected.

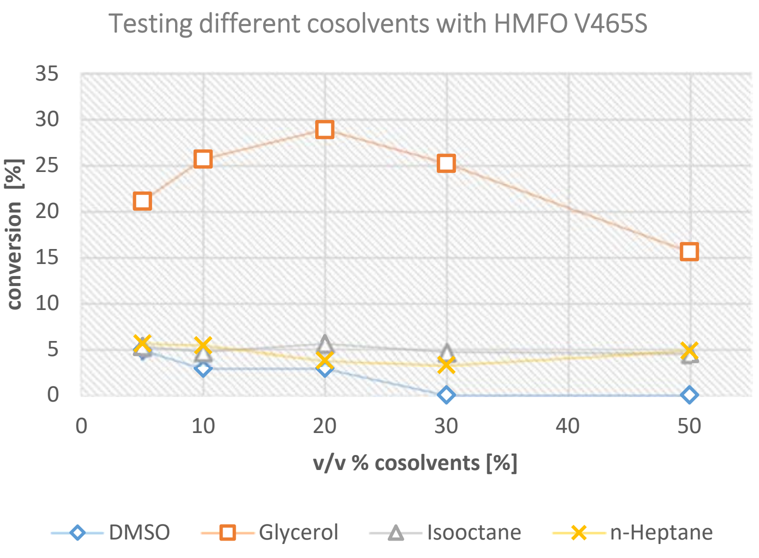

Figure S2. Cosolvent study with substrate **4a** by using HMFO V465S

ii) HMFO V465T

Results from testing V465T revealed that better conversions were achieved by using glycerol as cosolvent (Table S5, Figure S3).

Table S5. Cosolvent study with substrate **4a** by using HMFO V465T [a]

Entry	Cosolvent / v/v % of cosolvent	DMSO	Glycerol	Isooctane	*n*-Heptane
1	5	5	14	5	6
2	10	4	13	5	5
3	20	3	10	5	4
4	30	2	11	5	4
5	50	3	11	4	4

[a] Condition: KPi (200 mM, pH 7.0) containing the oxidases (1.4 µM final concentration in 1 mL reaction volume), catalase from *Micrococcus lysodeikticus* (30 µL, 170000 U/mL), the substrate (50 mM), 5% to 50% v/v of different cosolvents. The reaction mixtures and blanks were shaken 16 hours (170 rpm, 21 ºC) and extracted with ethyl acetate (2 x 500 µL), dried with Na_2SO_4 and measured with GC-MS (method: lowboilers) afterwards. Conversions were measured based on area ratio of ketone to substrate.
GC-MS Method: Injector temperature: 250 °C; Injection volume: 1 µL; Flow rate: 0.7 mL/min; Temperature program (low boilers method): 40 °C, hold time 2.0 min, 10 °C/min to 180 °C, hold time 1.0 min; EI mode, energy 70 eV, MS Source: 230 °C, MS Quadrupole: 150 °C. HP-5MS column (5% phenylmethylsiloxane, 30 m x 0.20 mm x 0.25 µm, J&W Scientific, Agilent Technologies) using He as carrier gas.
[b] No conversion was detected.

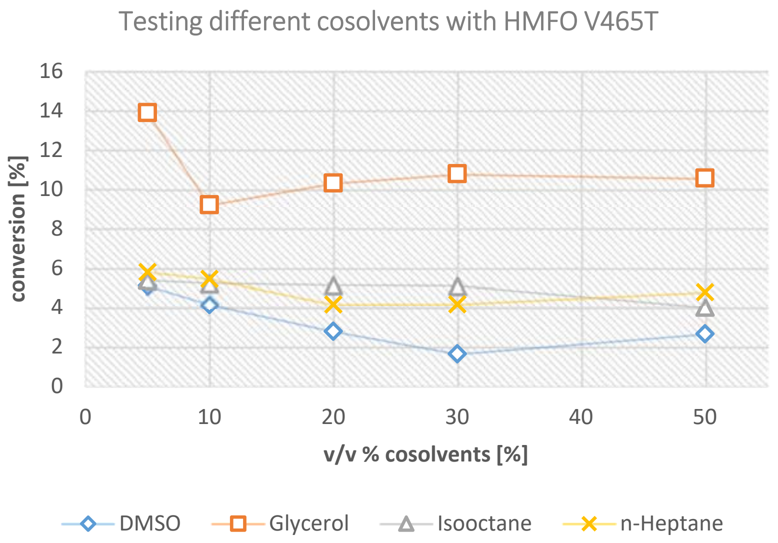

Figure S3. Cosolvent study with substrate **4a** by using HMFO V465T

iii) HMFO W466H

Results from testing W466H with different cosolvents are shown in Table S6 and Figure S4. As results reveal this mutation considerably reduced the activity of the enzyme since very poor conversions were observed by using this variant at different conditions.

Table S6. Cosolvent study with substrate **4a** by using HMFO W466H [a]

Entry	Cosolvent / v/v % of cosolvent	DMSO	Glycerol	Isooctane	*n*-Heptane
1	5	1	3	1	1
2	10	1	3	1	0.4
3	20	1	2	0.2	n.d.
4	30	n.d.	2	n.d.	n.d.
5	50	n.d.	2	n.d.	n.d.

[a] Condition: KPi (200 mM, pH 7.0) containing the oxidases (1.4 µM final concentration in 1 mL reaction volume), catalase from *Micrococcus lysodeikticus* (30 µL, 170000 U/mL), the substrate (50 mM), 5% to 50% v/v of different cosolvents. The reaction mixtures and blanks were shaken 16 hours (170 rpm, 21 ºC) and extracted with ethyl acetate (2 x 500 µL), dried with Na_2SO_4 and measured with GC-MS (method: lowboilers) afterwards. Conversions were measured based on area ratio of ketone to substrate.
GC-MS Method: Injector temperature: 250 °C; Injection volume: 1 µL; Flow rate: 0.7 mL/min; Temperature program (low boilers method): 40 °C, hold time 2.0 min, 10 °C/min to 180 °C, hold time 1.0 min; EI mode, energy 70 eV, MS Source: 230 °C, MS Quadrupole: 150 °C. HP-5MS column (5% phenylmethylsiloxane, 30 m x 0.20 mm x 0.25 µm, J&W Scientific, Agilent Technologies) using He as carrier gas.
[b] No conversion was detected.

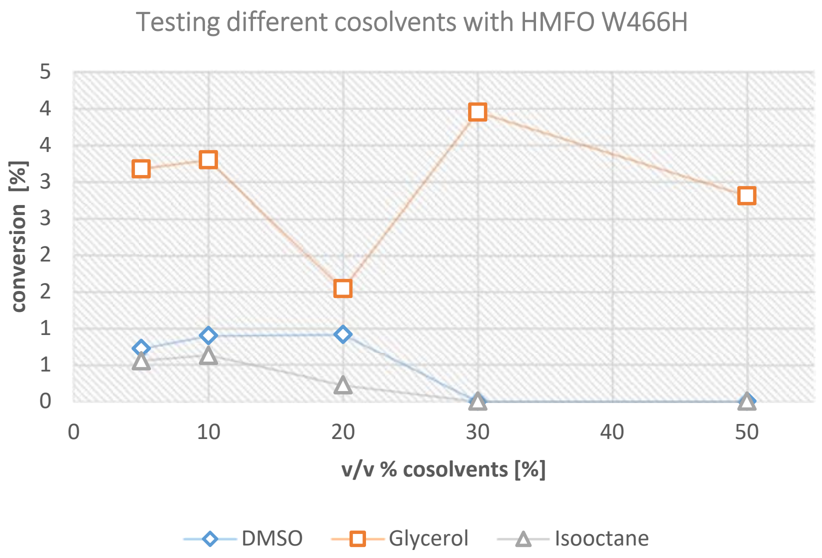

Figure S4. Cosolvent study with substrate **4a** by using HMFO W466H

iv) HMFO V465T/W466H

Results from oxidation of **4a** with V465T/W466H in the presence of various cosolvents revealed that better conversions were achieved by using glycerol as cosolvent (Table S7, Figure S5). By using 30% v/v of glycerol, conversion was reached to 25.1% (entry 3). *n*-Heptane was not accepted by the enzyme, since no conversion was observed in the presence of these cosolvents.

Table S7. Cosolvent study with substrate **4a** by using HMFO V465T/W466H [a]

Entry	Cosolvent / v/v % of cosolvent	DMSO	Glycerol	Isooctane	*n*-Heptane
1	5	3	10	3	n.d. [b]
2	10	3	20	4	0.7
3	20	2	25	4	n.d.
4	30	1	12	3	n.d.
5	50	n.d.	6	2	n.d.

[a] Condition: KPi (200 mM, pH 7.0) containing the oxidases (1.4 µM final concentration in 1 mL reaction volume), catalase from *Micrococcus lysodeikticus* (30 µL, 170000 U/mL), the substrate (50 mM), 5% to 50% v/v of different cosolvents. The reaction mixtures and blanks were shaken 16 hours (170 rpm, 21 ºC) and extracted with ethyl acetate (2 x 500 µL), dried with Na_2SO_4 and measured with GC-MS (method: lowboilers) afterwards. Conversions were measured based on area ratio of ketone to substrate.
GC-MS Method: Injector temperature: 250 °C; Injection volume: 1 µL; Flow rate: 0.7 mL/min; Temperature program (low boilers method): 40 °C, hold time 2.0 min, 10 °C/min to 180 °C, hold time 1.0 min; EI mode, energy 70 eV, MS Source: 230 °C, MS Quadrupole: 150 °C. HP-5MS column (5% phenylmethylsiloxane, 30 m x 0.20 mm x 0.25 µm, J&W Scientific, Agilent Technologies) using He as carrier gas.
[b] No conversion was detected.

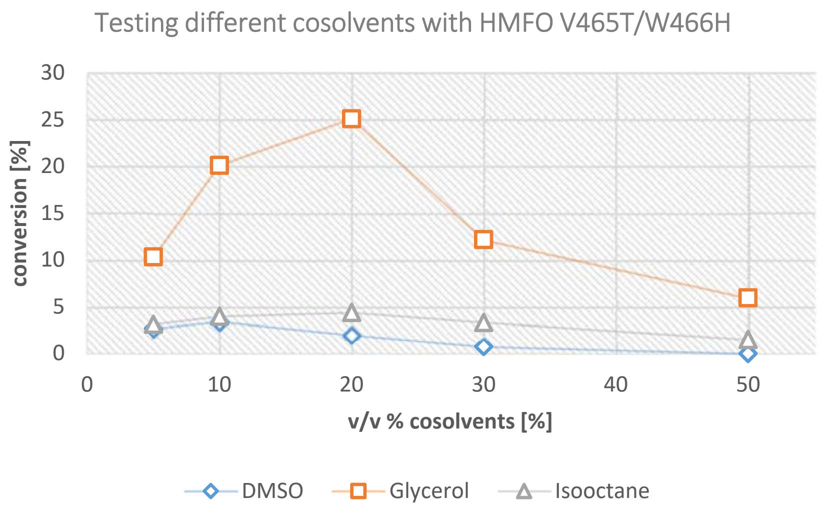

Figure S5. Cosolvent study with substrate **4a** by using HMFO V465T/W466H

2.1.2. Investigating the oxygen pressure effect on the oxidation of various substrates by using two different variants of HMFO

Substrate screening in the oxidation step was done by using two variants of HMFO including V465S and V465T in the presence of 10% v/v of DMSO and glycerol as cosolvent. The reactions were performed at two different conditions (with and without oxygen pressure) in parallel. Results revealed that in most of the cases, by using V465T variants higher conversion was achieved. It is worth to mention that in case of α-ionol (**6a**) very low conversion level (<3%) was observed in all tested conditions. In general by using DMSO as cosolvent low conversions were obtained. In the same line as the other results, higher conversions were obtained by using glycerol as cosolvent. Results are shown in Table S8.

Table S8. Substrate screening by using HMFO V465S and V465T with and without O_2 pressure [a]

Entry	Substr.	Cosolvent	Variant	Conv. [%] (without O_2 pressure)	Conv. [%] (with 1.5 bar O_2 pressure)
1	**1a**	DMSO	V465S	9	5
2	**1a**	DMSO	V465T	12	8
3	**1a**	Glycerol	V465S	24	20
4	**1a**	Glycerol	V465T	14	10
5	**2a**	DMSO	V465S	20	24
6	**2a**	DMSO	V465T	27	29
7	**2a**	Glycerol	V465S	47	49
8	**2a**	Glycerol	V465T	55	57
9	**3a**	DMSO	V465S	8	11
10	**3a**	DMSO	V465T	11	15
11	**3a**	Glycerol	V465S	27	30
12	**3a**	Glycerol	V465T	11	43
13	**4a**	DMSO	V465S	5	5
14	**4a**	DMSO	V465T	3	6
15	**4a**	Glycerol	V465S	55	58
16	**4a**	Glycerol	V465T	50	54
17	**5a**	DMSO	V465S	2	5
18	**5a**	DMSO	V465T	2	6
19	**5a**	Glycerol	V465S	35	50
20	**5a**	Glycerol	V465T	13	24
21	**6a**	DMSO	V465S	<1	3
22	**6a**	DMSO	V465T	<1	3
23	**6a**	Glycerol	V465S	<1	3
24	**6a**	Glycerol	V465T	<1	3

[a] Condition: KPi (200 mM, pH 7.0) containing the oxidases (2.1 µM final concentration in 1 mL reaction volume), catalase from *Micrococcus lysodeikticus* (30 µL, 170000 U/mL), the substrate (50 mM), 10% v/v DMSO or glycerol as cosolvent. The reaction mixtures and blanks were shaken 16 hours (170 rpm, 21 °C; additional 1.5 bar O_2 for the mixtures with O_2 pressure) and extracted with ethyl acetate (2 x 500 µL), dried with Na_2SO_4 and measured with GC-MS (method: lowboilers) afterwards. Conversions were measured based on area ratio of ketone to substrate. Reactions have been done in duplicate and the average of data was reported.
GC-MS Method: Injector temperature: 250 °C; Injection volume: 1 µL; Flow rate: 0.7 mL/min; Temperature program (low boilers method): 40 °C, hold time 2.0 min, 10 °C/min to 180 °C, hold time 1.0 min; EI mode, energy 70 eV, MS Source: 230 °C, MS Quadrupole: 150 °C. HP-5MS column (5% phenylmethylsiloxane, 30 m x 0.20 mm x 0.25 µm, J&W Scientific, Agilent Technologies) using He as carrier gas.
[b] No conversion was detected.

In the oxidation of substrate **1a**, by applying oxygen pressure the conversion level dropped. In the oxidation of substrates **2a**, **3a** and **4a** (aromatic substrates) using HMFO V465T and V465S variants, the oxygen did not have a high impact on the conversion level. In the oxidation of substrate **5a**, with using different variants by applying oxygen pressure, especially in case of glycerol, an improvement in terms of conversion was observed.

The results from oxidation of substrates **3a-5a** with different HMFO variants as well as wild type without using any cosolvent in the presence of air, 2 and 4 bar oxygen pressure are shown in Table S9.

Table S9. Oxidation of *sec*-allylic alcohols **3a**-**5a** employing HMFO variants in the presence of air, 2 and 4 bar O_2 pressure [a]

Entry	Substr.	Variant	Conv. [%]		
			air	O_2 (2 bar)	O_2 (4 bar)
1	**3a**	wt	10	15	27
2	**3a**	V465T	48	46	42
3	**3a**	V465S	50	46	42
4	**3a**	V465T/W466H	50	48	48
5	**3a**	V367R/W466F	46	45	48
6	**4a**	wt	13	20	34
7	**4a**	V465T	48	49	44
8	**4a**	V465S	48	48	42
9	**4a**	V465T/W466H	50	40	42
10	**4a**	V367R/W466F	32	36	40
11	**5a**	wt	4	4	15
12	**5a**	V465T	32	37	49
13	**5a**	V465S	38	38	47
14	**5a**	V465T/W466H	4	4	10
15	**5a**	V367R/W466F	4	4	8

[a] Condition: KPi (200 mM, pH 7.0) containing the oxidases (14.2 µM final concentration in 500 µL reaction volume), catalase from *Micrococcus lysodeikticus* (15 µL, 170000 U/mL), **3a**-**5a** (50 mM). The reaction mixtures were shaken 16 hours (170 rpm, 21 ºC) and extracted with ethyl acetate (2 x 500 µL), dried with Na_2SO_4 and analyzed by GC-FID. Conversions were measured based on area ratio of ketone to substrate. Reactions have been done in duplicate.

2.1.3. NMR of purified ketones from upscaling reaction with HMFO V465S

i) (E)-oct-3-en-2-one (1b)

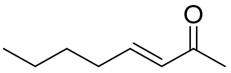

310 mg (70% isolated yield, colorless oil), ^{1}H NMR (300 MHz, $CDCl_3$): δ 6.73 (1H, dt, *J* = 15.9, 6.9 Hz, $CH_2C\mathbf{H}$=), 5.99 (1H, d, *J*= 16.0 Hz, =C**H**C(O)), 2.24-2.07 (5H, m, =CHC$\mathbf{H_2}$ + C$\mathbf{H_3}$C(O)), 1.50-1.18 (4H, m, $CH_3C\mathbf{H_2}C\mathbf{H_2}$), 0.84 (3H, t, *J* = 7.1 Hz, C$\mathbf{H_3}$$CH_2$).; ^{13}C NMR (75 MHz, $CDCl_3$): δ 198.5, 148.4, 131.2, 32.0, 30.1, 26.7, 22.1, 13.7.[3]

ii) (E)-4-phenylbut-3-en-2-one (2b)

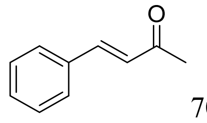

70 mg (33% isolated yield, pale yellow solid, mp 38-42 ºC), ^{1}H NMR (300 MHz, $CDCl_3$): δ 7.58 – 7.40 (6H, m, $C_6\mathbf{H_5}$ + $C_6H_5\mathbf{H}$C=), 6.73 (1H, d, *J* = 16.3 Hz, =C**H**CO), 2.40 (3H, s, C$\mathbf{H_3}$). ^{13}C NMR (75 MHz, $CDCl_3$) δ 1198.4, 143.43, 134.4, 130.5, 129.0, 128.3, 127.2, 27.5.[5]

iii) (E)-4-(4-chlorophenyl)but-3-en-2-one (3b)

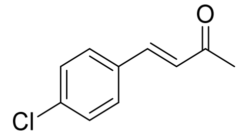

104.1 mg (54% isolated yield, colorless solid, mp 58-62 ºC), ^{1}H NMR (300 MHz, $CDCl_3$) δ 7.50– 7.37 (m, 5H, Ar + Ar**H**C=), 6.70 (d, *J* = 16.3 Hz, 1H, =C**H**CO), 2.40 (s, 3H, COC$\mathbf{H_3}$). ^{13}C NMR (75 MHz, $CDCl_3$) δ 198.1, 141.9, 136.4, 132.9, 129.4, 129.3, 127.5, 27.7.[7]

iv) (E)-4-(4-methylphenyl)but-3-en-2-one (4b)

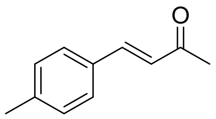

180 mg (64% isolated yield, pale yellow solid, mp 29-33 ºC), ^{1}H NMR (300 MHz, $CDCl_3$) δ 7.54– 7.45 (m, 3H, Ar + Ar**H**C=), 7.22 (d, J = 8.0 Hz, 2H, Ar), 6.70 (d, J = 16.2 Hz, 1H, =C**H**CO), 2.39 (d, J = 2.4 Hz, 6H, 2 x C**H$_3$**). ^{13}C NMR (75 MHz, $CDCl_3$) δ 198.5, 143.5, 141.0, 131.7, 129.7, 128.3, 126.3, 27.4, 21.5.[7]

v) β-ionone (5b)

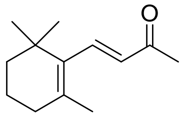

50 mg (31% isolated yield, colorless oil), ^{1}H NMR (300 MHz, $CDCl_3$) δ 7.31– 7.26 (m, 1H, =C**H**), 6.13 (d, J = 16.4 Hz, 1H, =C**H**CO), 2.31 (s, 3H,COC**H$_3$**), 2.08 (t, J = 6.2 Hz, 2H, C**H$_2$**), 1.78 (d, J = 0.6 Hz, =CC**H$_3$**), 1.72 – 1.56 (m, 2H, C**H$_2$**), 1.55 – 1.44 (m, 2H, C**H$_2$**), 1.08 (s, 6H, 2 x C**H$_3$**). ^{13}C NMR (75 MHz, $CDCl_3$) δ 198.8, 143.2, 136.1, 135.9, 131.6, 39.7, 34.1, 33.6, 28.8, 27.2, 21.7, 18.9.[9]

3. Analytics

All obtained substrates and reference compounds were analyzed by ^{1}H NMR and ^{13}C NMR. ^{1}H and ^{13}C NMR spectra were recorded using a 300 and 75 MHz instrument, respectively. Chemical shifts (*δ*) are given in parts per million (ppm) relative to TMS (*δ* = 0 ppm) or to the residual solvent signal, and coupling constants (*J*) are reported in Hertz (Hz). Thin layer chromatography was carried out on silica gel 60 F254 plates and compounds were visualized by UV.

GC-MS:

The reference materials were used for co-injection on GC-MS. GC-MS measurements were carried out on a 7890A GC System (Agilent Technologies, Santa Clara, CA, USA), equipped with a 5975C mass selective detector and a HP-5MS column (5% phenylmethylsiloxane, 30 m x 0.20 mm x 0.25 µm, J&W Scientific, Agilent Technologies) using He as carrier gas. Injector temperature: 250 °C; Injection volume: 1 µL; Flow rate: 0.7 mL/min; Temperature program (low boilers method): 40 °C, hold time 2.0 min, 10 °C/min to 180 °C, hold time 1.0 min; EI mode, energy 70 eV, MS Source: 230 °C, MS Quadrupole: 150 °C.

HPLC:

The enantiometric excess of the remaining substrates was determined by using chiral GC or HPLC. Chiral HPLC measurements were done by using OD-H and AS-H chiral columns using different methods, which are mentioned in Tables S10-S13.

Table S10. Retention times of allylic alcohol (**2a**) and its corresponding ketone (**2b**) measured with Shimadzu HPLC equipped with OD-H column [a]

	**Retention time [min]** [12]	
	**X= OH alcohol**	**X= =O ketone**
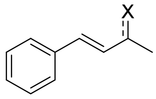	10.8 (*R*), 15.3 (*S*) [12]	9.7

[a] Method: OD-H, *n*-Hep: *i*PrOH 90:10, Flow: 0.7 mL/min, 230 nm, Temperature: 25 ºC.

Table S11. Retention times of allylic alcohol (**4a**) and its corresponding ketone (**4b**) measured with Shimadzu HPLC equipped with OD-H column [a]

	**Retention time [min]** [12]	
	**X= OH alcohol**	**X= =O ketone**
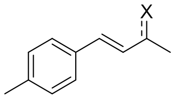	33.6 (*R*), 42.2 (*S*) [12]	9.4

[a] Method: OD-H, *n*-Hep: *i*PrOH 99:1, Flow: 1.0 mL/min, 230 nm, Temperature: 30 ºC.

Table S12. Retention times of allylic alcohol (**3a**) and its corresponding ketone (**3b**) measured with Shimadzu HPLC equipped with AS-H column [a]

	**Retention time [min]** [13]	
	**X= OH alcohol**	**X= =O ketone**
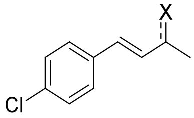	32.3 (*S*), 34.3 (*R*) [13]	40.6

[a] Method: AS-H, *n*-Hep: *i*PrOH 97:3, Flow: 0.5 mL/min, 230 nm, Temperature: 30 ºC.

Table S13. Retention times of allylic alcohol (**5a**) and its corresponding ketone (**5b**) measured with Shimadzu HPLC equipped with AS-H column [a]

	**Retention time [min]** [14]	
	**X= OH alcohol**	**X= =O ketone**
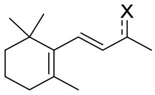	11.8 (*S*), 14.3 (*R*) [14]	24.0

[a] Method: AS-H, *n*-Hep: *i*PrOH 99:1, Flow: 0.5 mL/min, 230 nm, Temperature: 25 ºC.

GC with a achiral phase:

For substrate **1a,** GC equipped with chiral column was used. Chiral GC measurements were performed on an Agilent Technologies 7890 A GC system equipped with a FID-detector and a 7683B injector in combination with a 7683 Series Autosampler and using a Chirasil ChiralDexCB column (25m x 320µm x 0.25µm) and H_2 as carrier gas. Injector temperature: 250 °C; Injection volume: 5 µL; Flow rate: 1.7 mL/min; Detector temperature: 250 °C; Temperature program 1 (for non-derivatized substrate **1a**): 60 °C, hold time 1 min; 2 °C/ min to 65 °C, hold time 1 min; 2 °C/min, 70 °C, hold time 6 min; 2 °C/ min to 75 °C, hold time 8 min; 2 °C/min, 80 °C, hold time 8 min; 5 °C/min, 90 °C, hold time 1 min. Retention

times are shown in the Table S14. Temperature program 2 (for derivatized substrate **1a**): 50 °C, hold time 8 min; 10 °C/ min to 150 °C, hold time 2 min. Retention times related to this temperature program are shown in the Table S15.

Table S14. Retention times for allylic alcohol (**1a**) and its corresponding ketone (**1b**) measured by GC using a Chirasil ChiralDexCB column (25m x 320µm x 0.25µm) and H_2 as carrier gas [a]

	**Retention time [min]**	
	**X= OH**<br>**alcohol**	**X= =O**<br>**ketone**
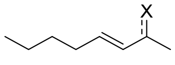	19.7 (*R*), 20.5 (*S*) [b]	15.4

[a] Injector temperature: 250 °C; Injection volume: 5 µL; Flow rate: 1.7 mL/min; Detector temperature: 250 °C; Temperature program 1: 60 °C, hold time 1 min; 2 °C/ min to 65 °C, hold time 1 min; 2 °C/min, 70 °C, hold time 6 min; 2 °C/ min to 75 °C, hold time 8 min; 2 °C/min, 80 °C, hold time 8 min; 5 °C/min, 90 °C, hold time 1 min.
[b] Absolute configuration was confirmed using derivatized alcohol.

Table S15. Retention times for derivatized allylic alcohol (**1a**) and its corresponding ketone (**1b**) measured by GC using a Chirasil ChiralDexCB column (25m x 320µm x 0.25µm) and H_2 as carrier gas [a]

	**Retention time [min]**	
	**X= OAc**<br>**Derivatized alcohol**	**X= =O**<br>**ketone**
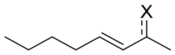	14.6 (*S*), 15.0 (*R*) [15]	13.9

[a] Injector temperature: 250 °C; Injection volume: 1 µL; Flow rate: 1.7 mL/min; Detector temperature: 250 °C; Temperature program 2: 50 °C, hold time 8 min; 10 °C/ min to 150 °C, hold time 2 min

4. Supplementary

4.1. NMRs

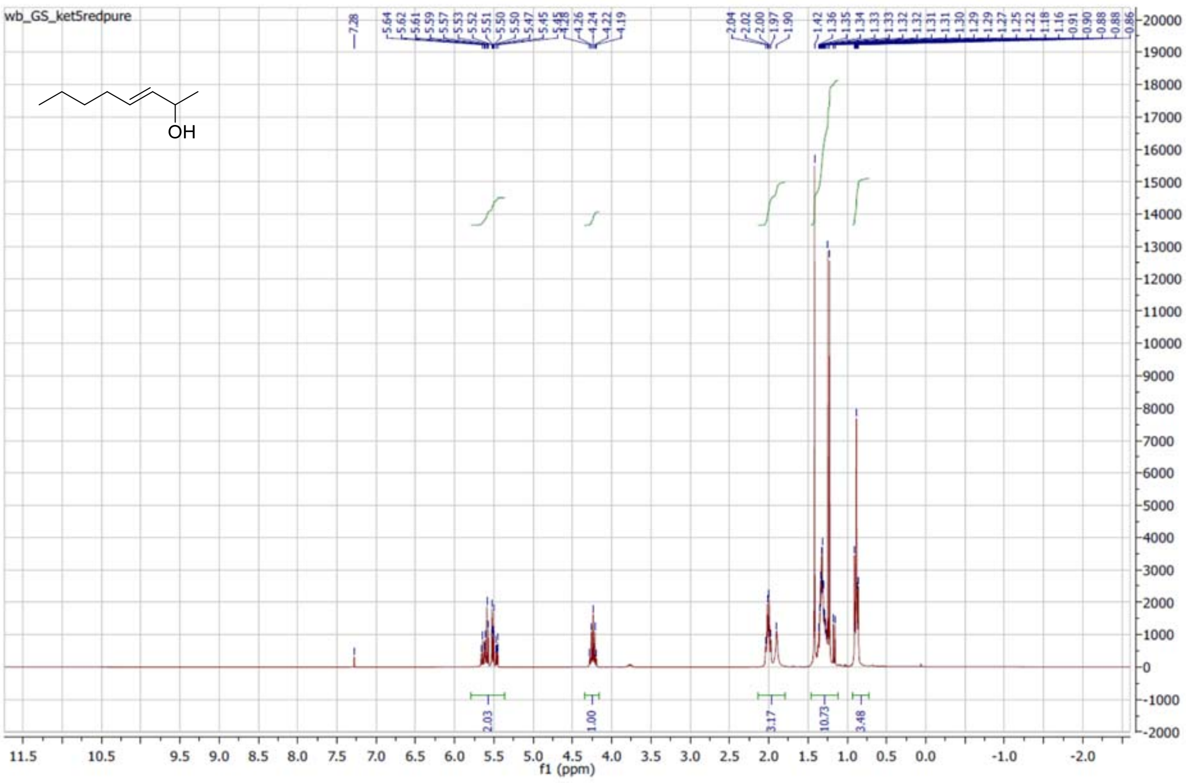

Figure S6. ^{1}H NMR of (*E*)-oct-3-en-2-ol (**1a**) in $CDCl_3$

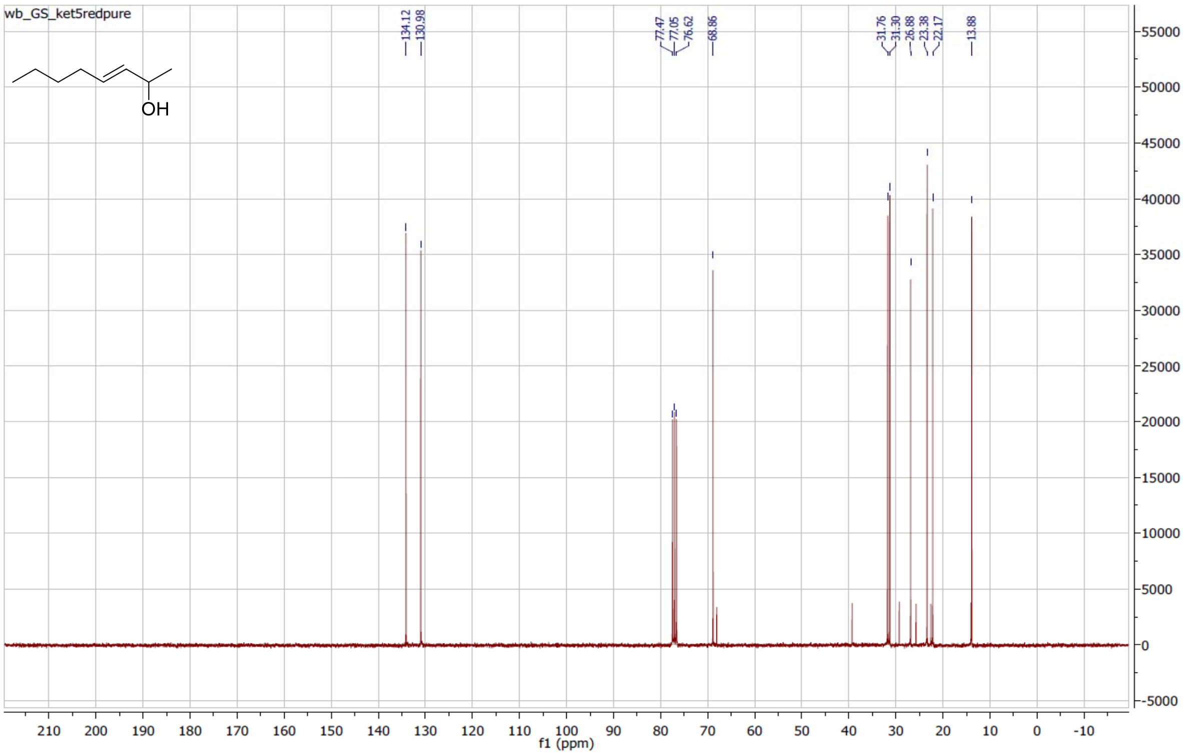

Figure S7. ^{13}C NMR of (*E*)-oct-3-en-2-ol (**1a**) in $CDCl_3$

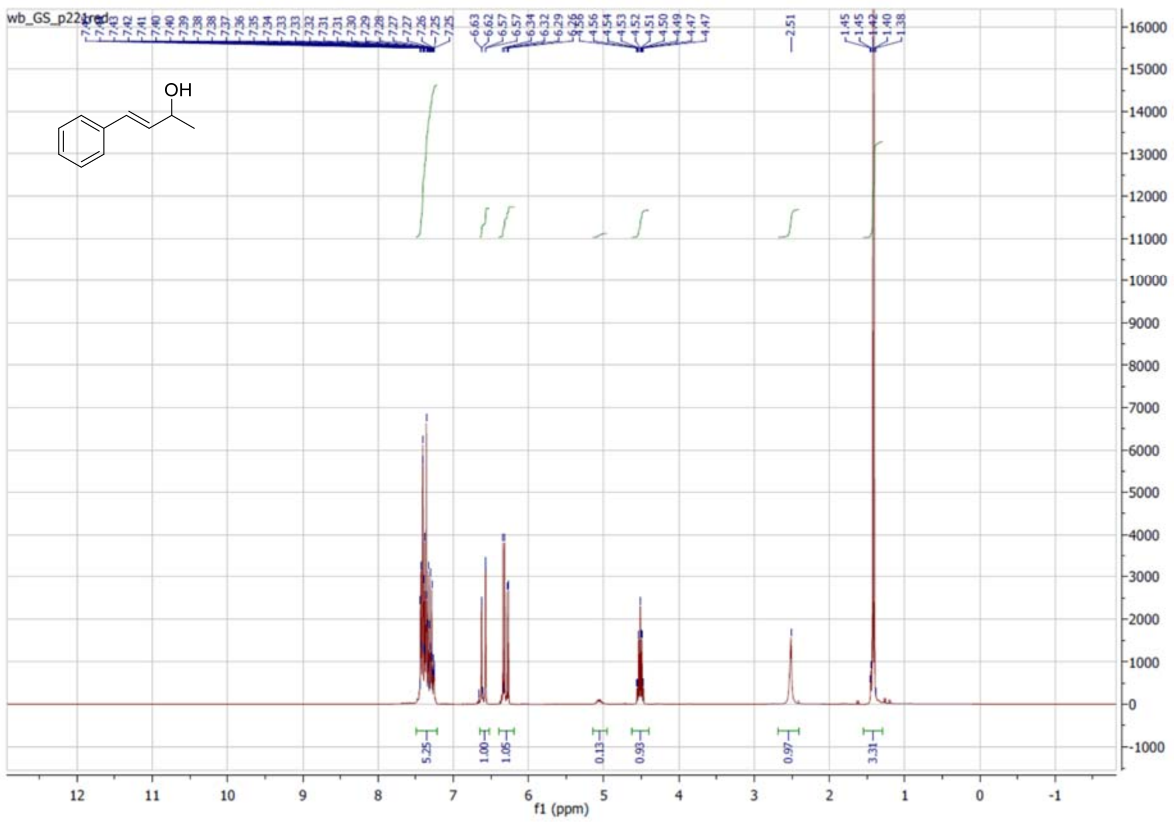

Figure S8. ^{1}H NMR of 4-phenylbut-3-en-2-ol (**2a**) in $CDCl_3$

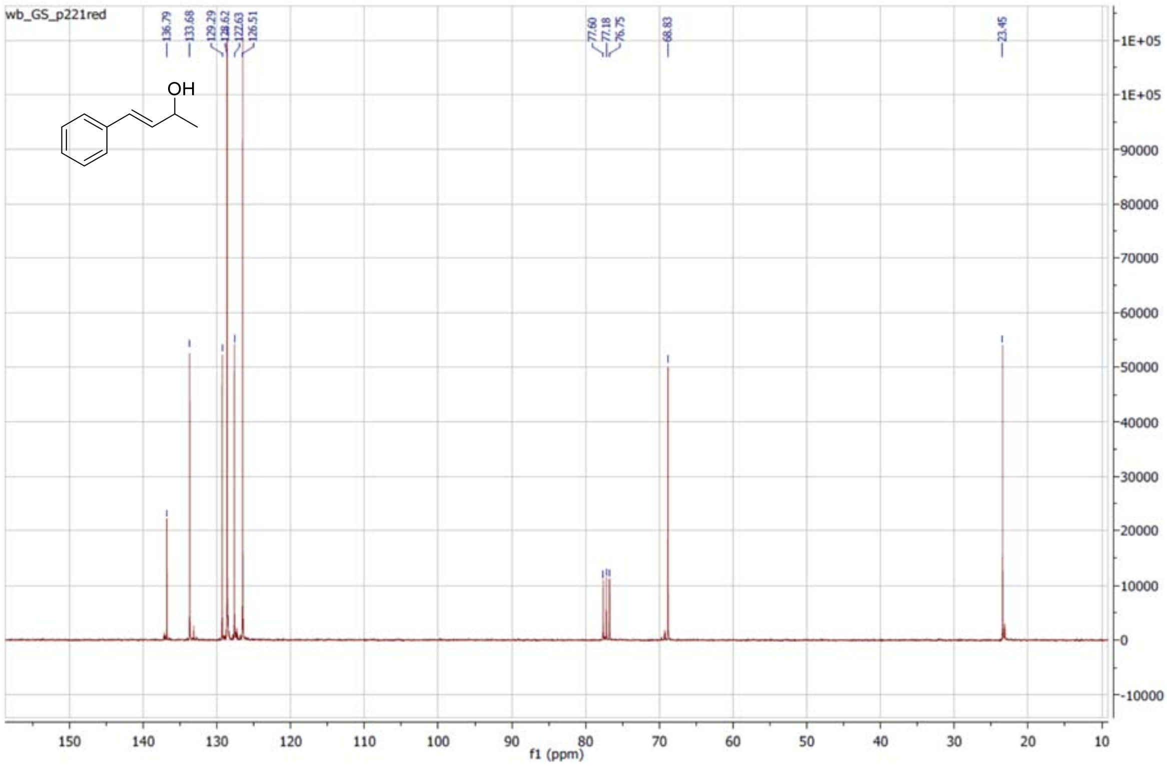

Figure S9. ^{13}C NMR of 4-phenylbut-3-en-2-ol (**2a**) in $CDCl_3$

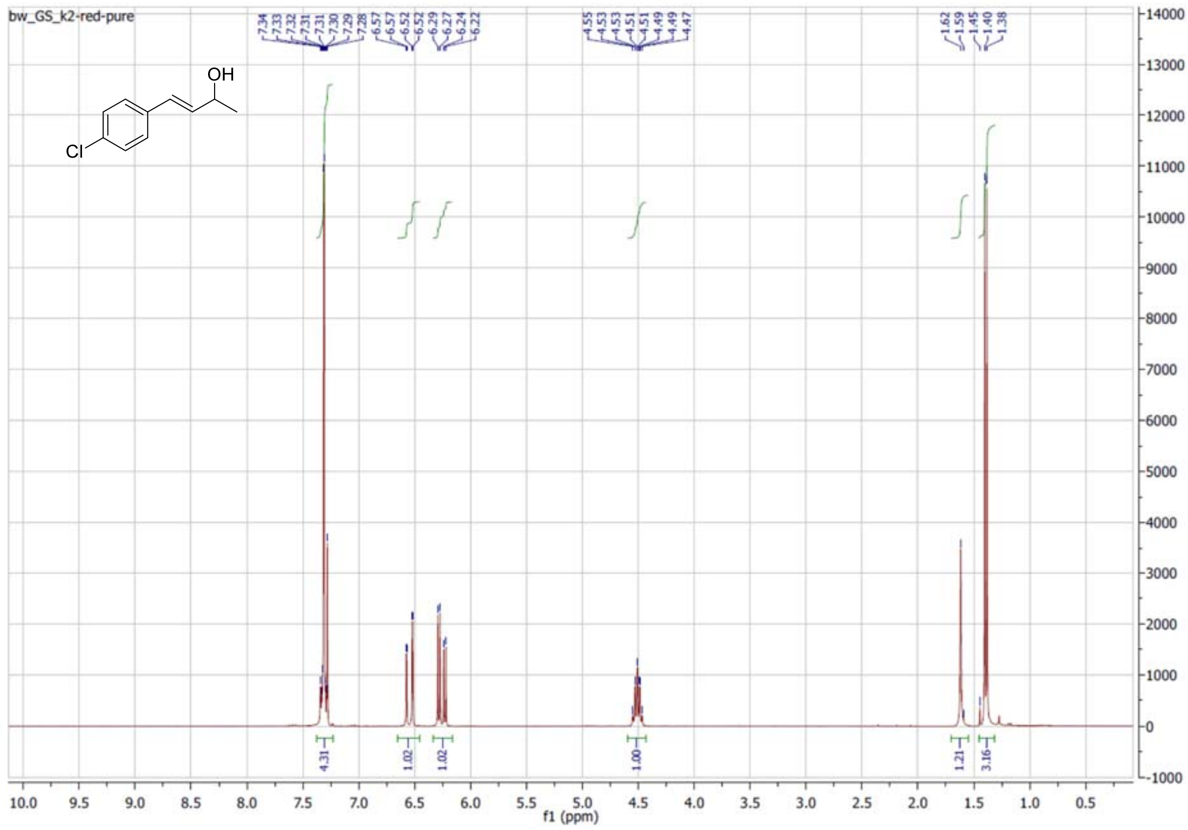

Figure S10. ^{1}H NMR of 4-(4-chlorophenyl)but-3-en-2-ol (**3a**) in $CDCl_3$

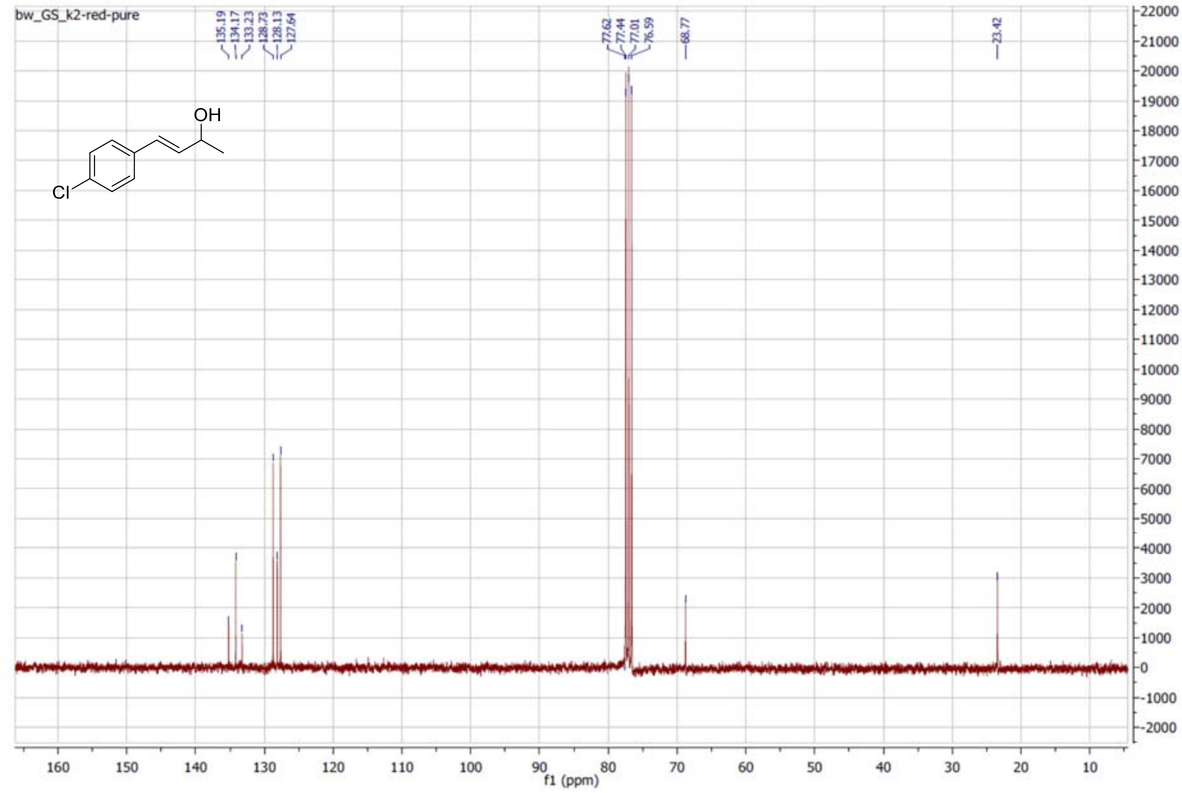

Figure S11. ^{13}C NMR of 4-(4-chlorophenyl)but-3-en-2-ol (**3a**) in $CDCl_3$

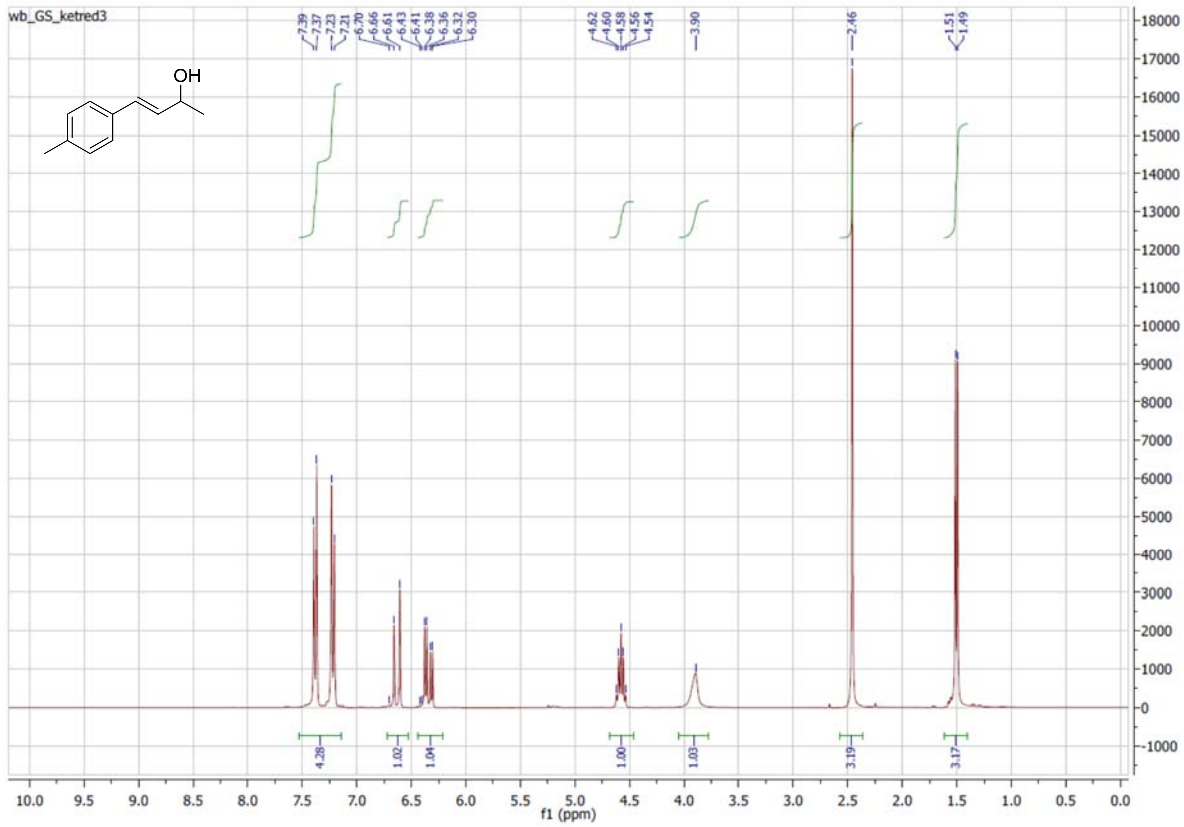

Figure S12. ^{1}H NMR of 4-(4-methylphenyl)but-3-en-2-ol (**4a**) in $CDCl_3$

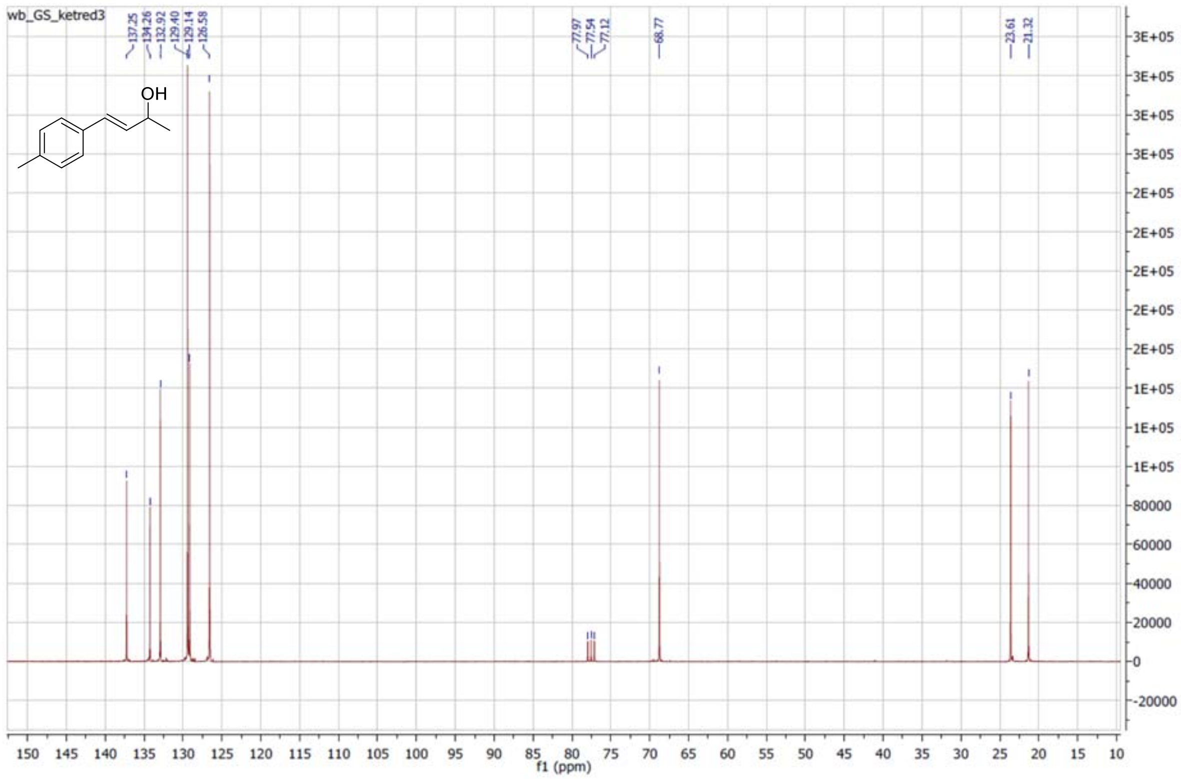

Figure S13. ^{13}C NMR of 4-(4-methylphenyl)but-3-en-2-ol (**4a**) in $CDCl_3$

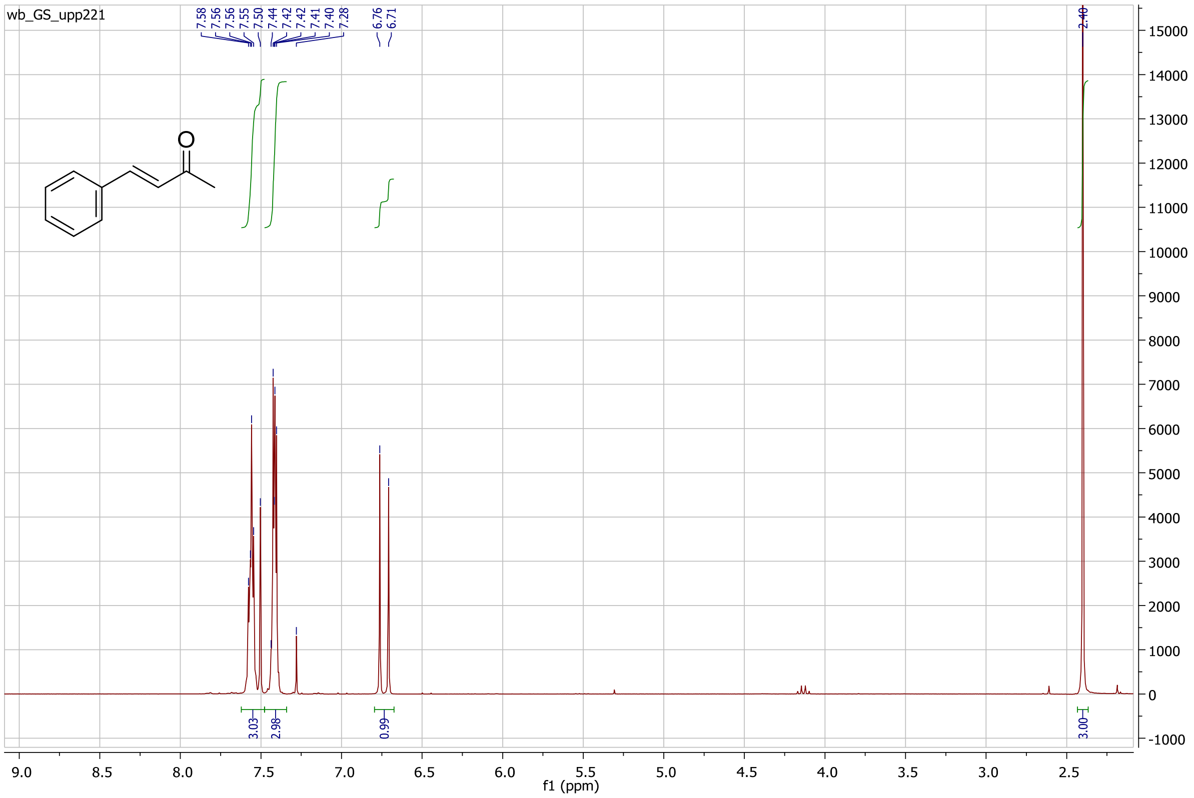

Figure S14. ^{1}H NMR of **2b** purified from upscaling in $CDCl_3$

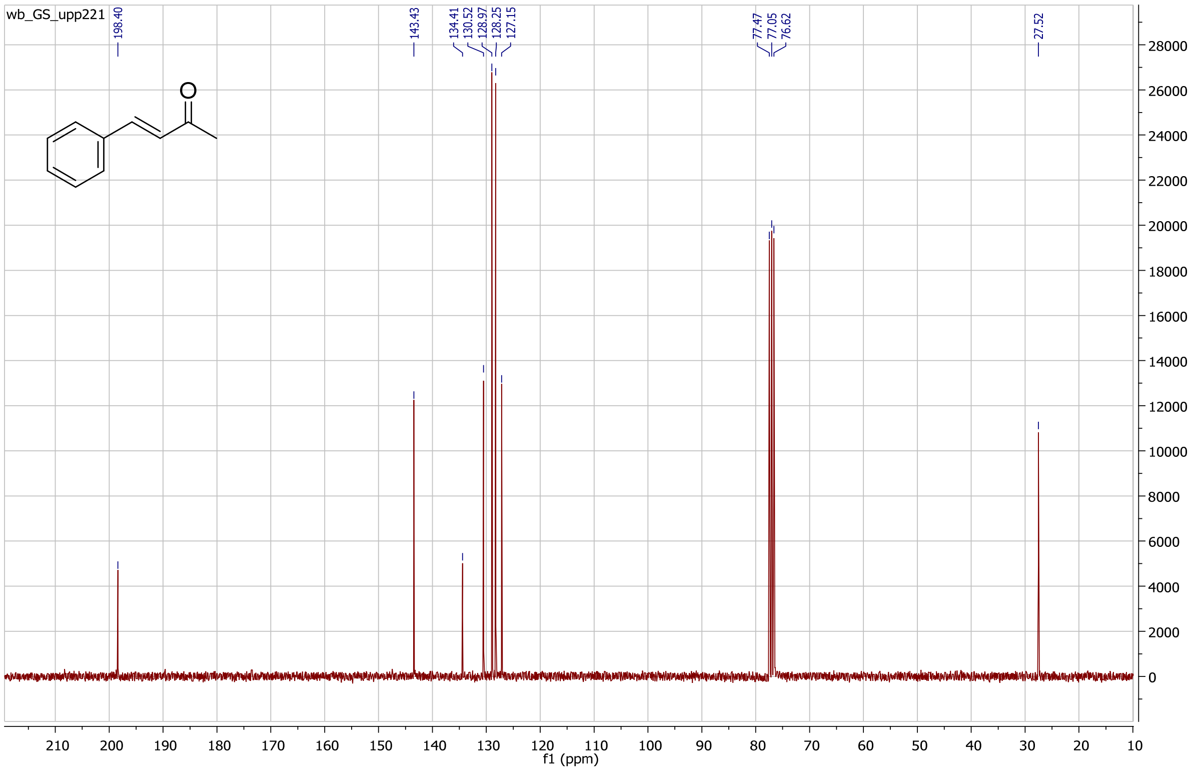

Figure S15. ^{13}C NMR of **2b** purified from upscaling in $CDCl_3$

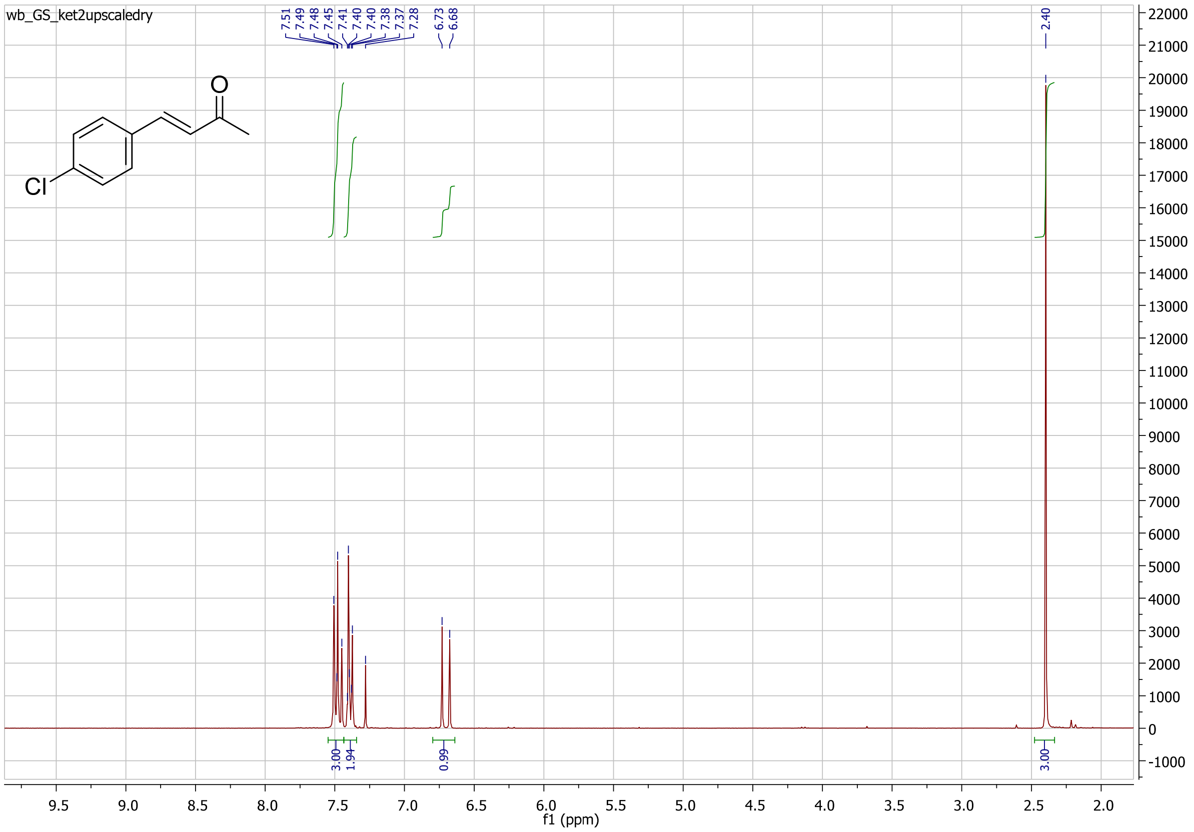

Figure S16. ^{1}H NMR of **3b** purified from upscaling in $CDCl_3$

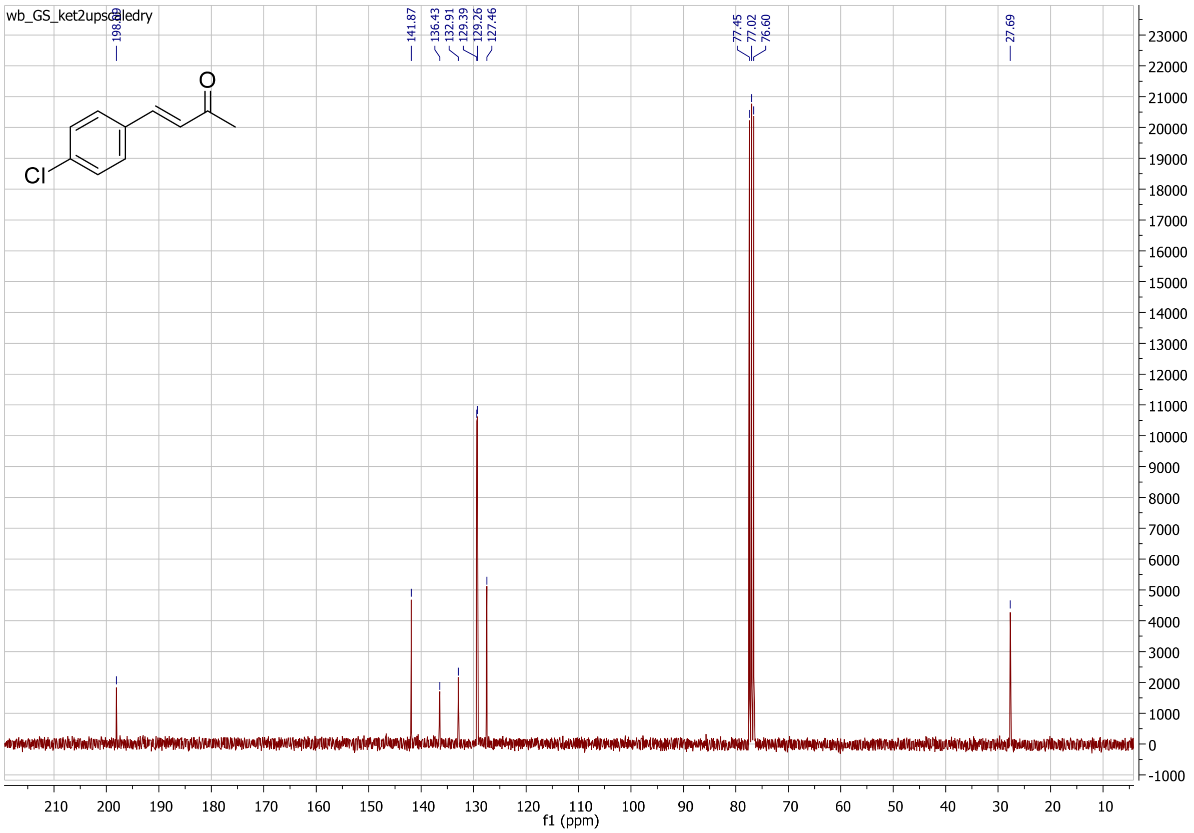

Figure S17. ^{13}C NMR of **3b** purified from upscaling in $CDCl_3$

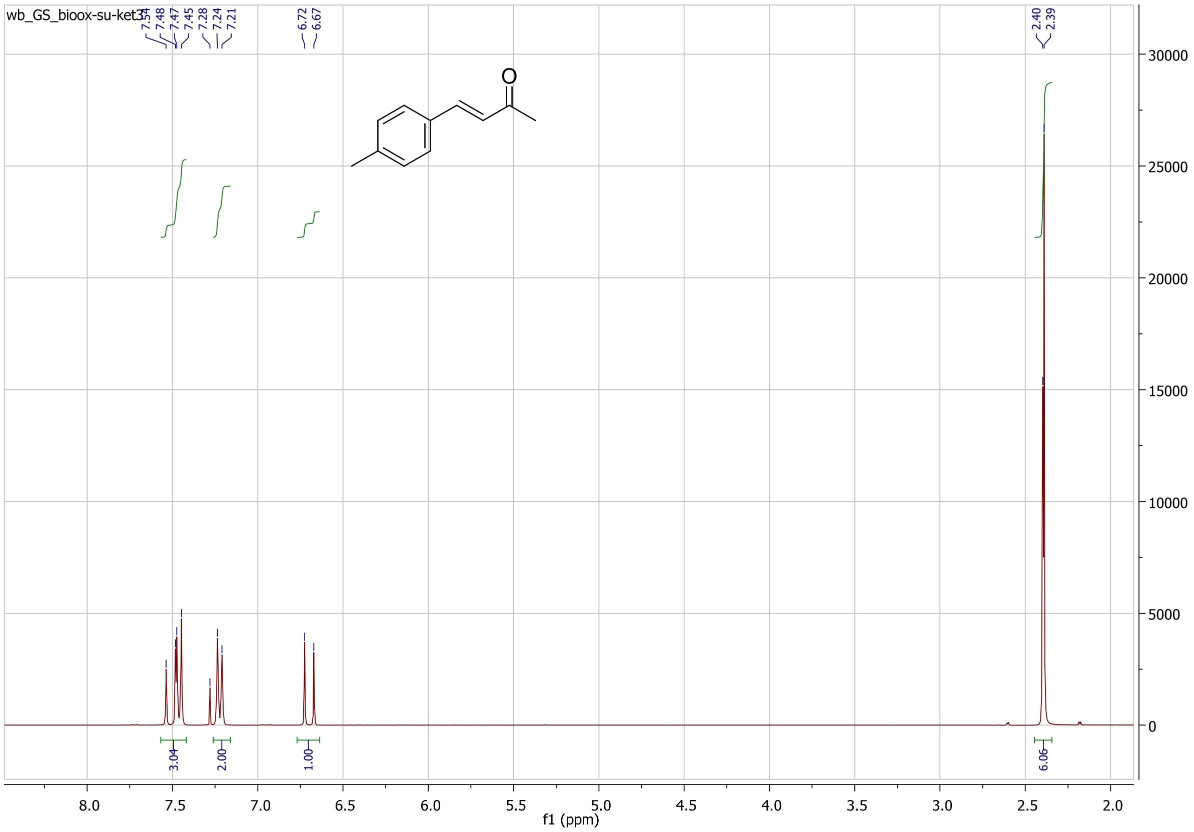

Figure S18. ^{1}H NMR of **4b** purified from upscaling in $CDCl_3$

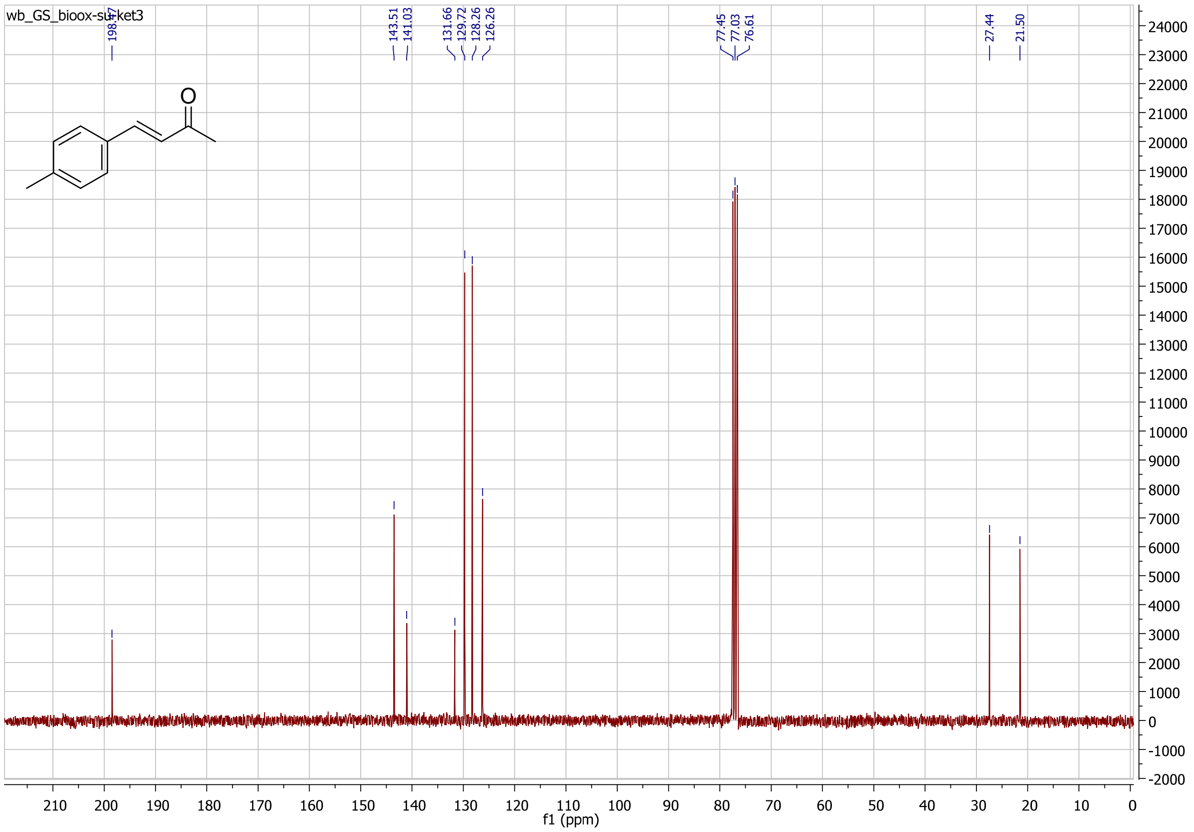

Figure S19. ^{13}C NMR of **4b** purified from upscaling in $CDCl_3$

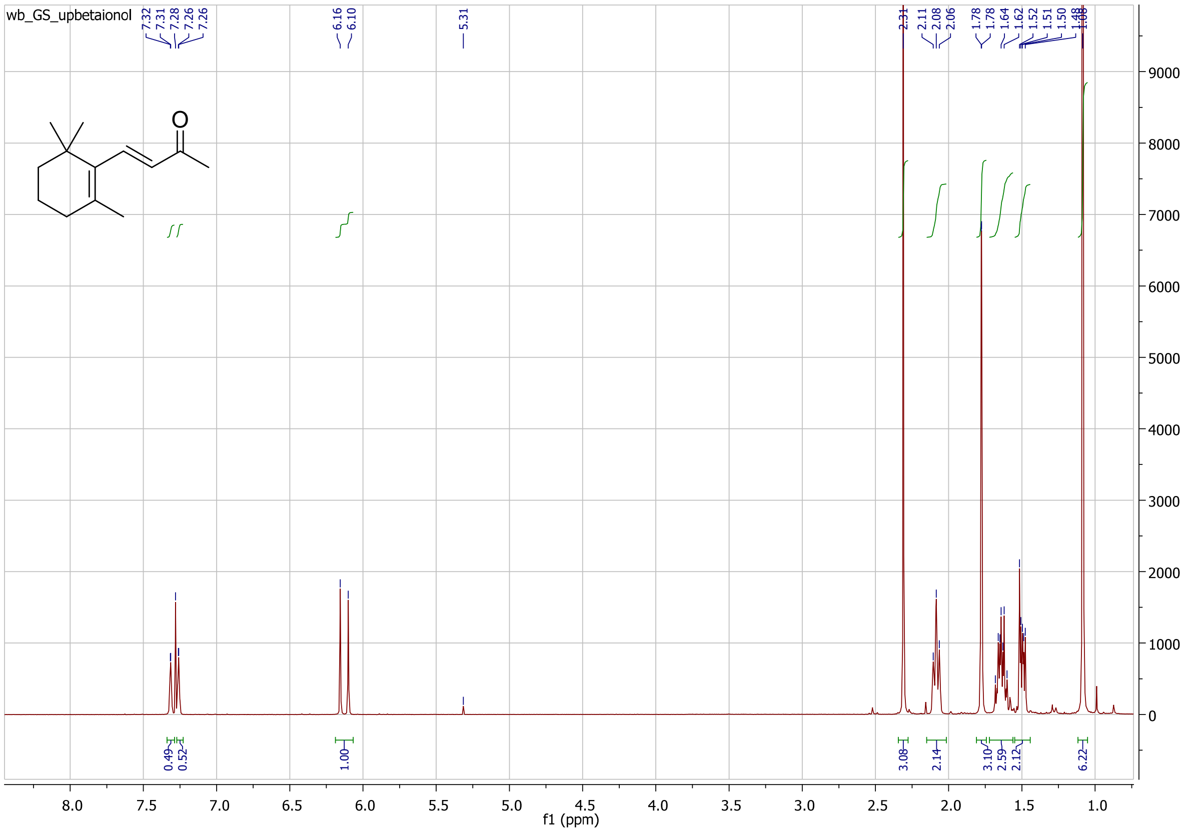

Figure S20. ^{1}H NMR of **5b** purified from upscaling in $CDCl_3$

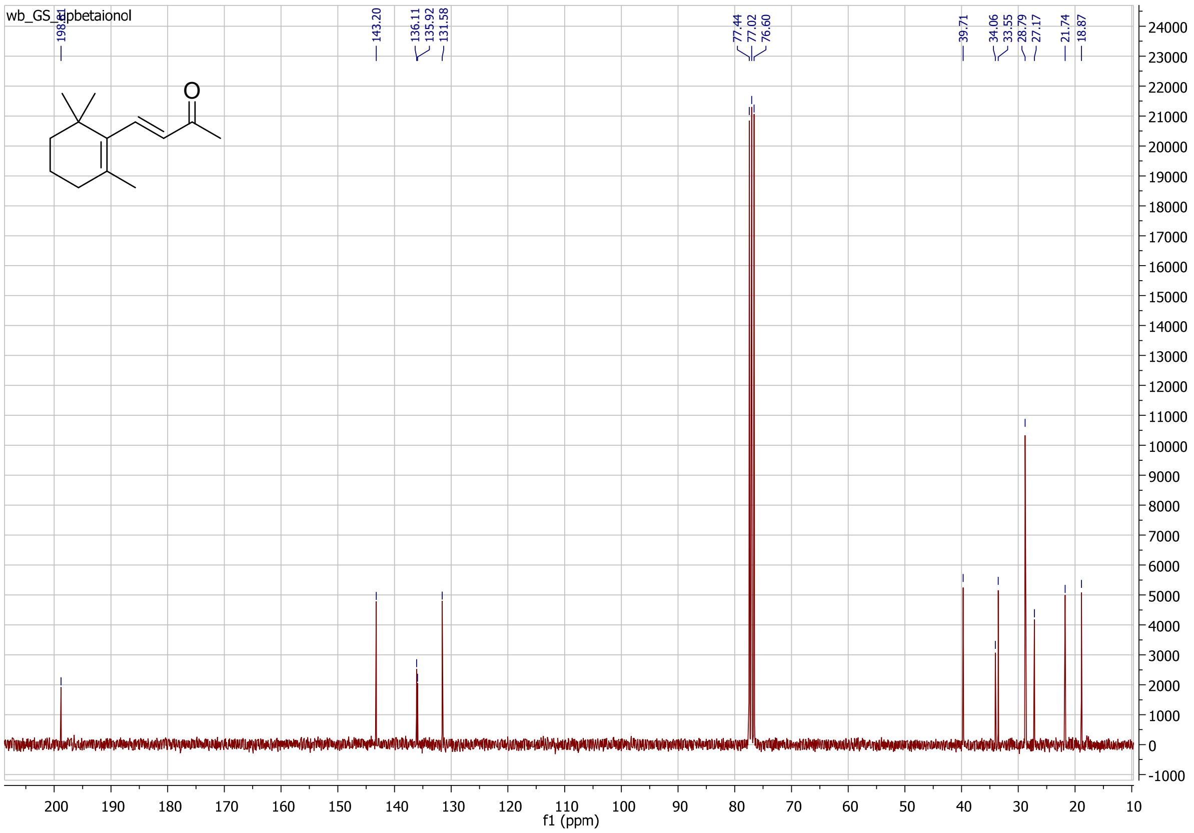

Figure S21. ^{13}C NMR of **5b** purified from upscaling in $CDCl_3$

4.2. GC-MS chromatograms

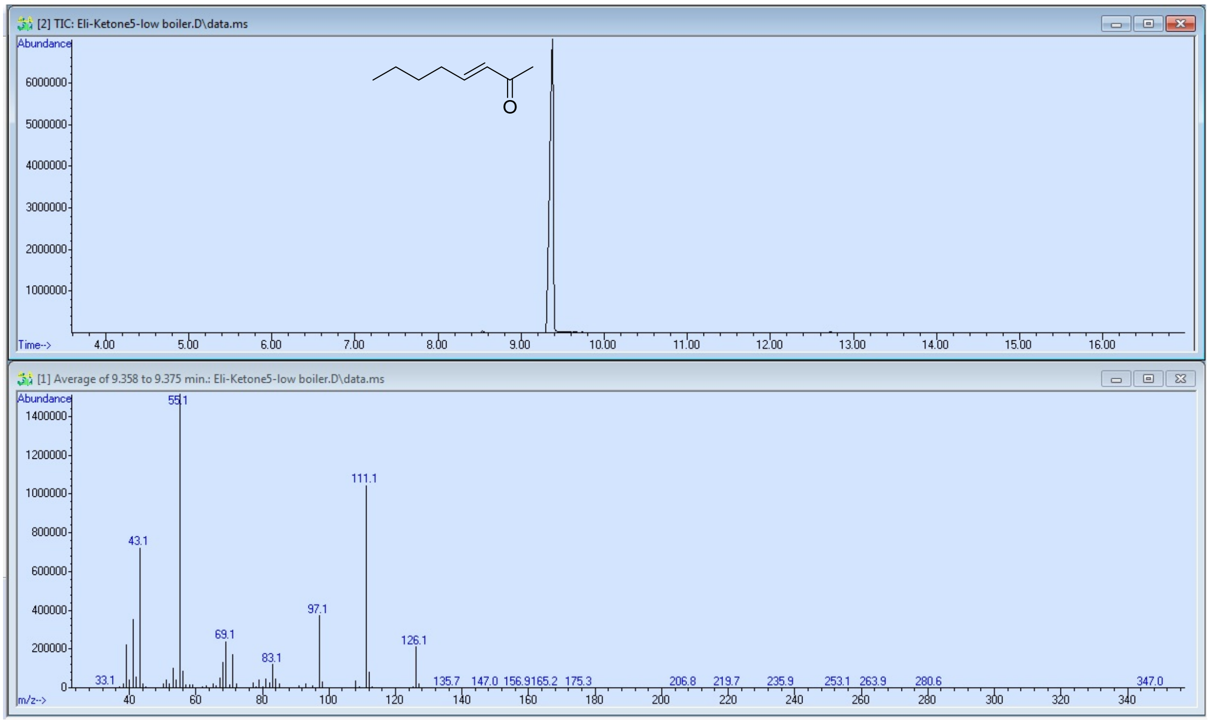

Figure S22. GC-MS chromatogram of (*E*)-oct-3-en-2-one (**1b**)

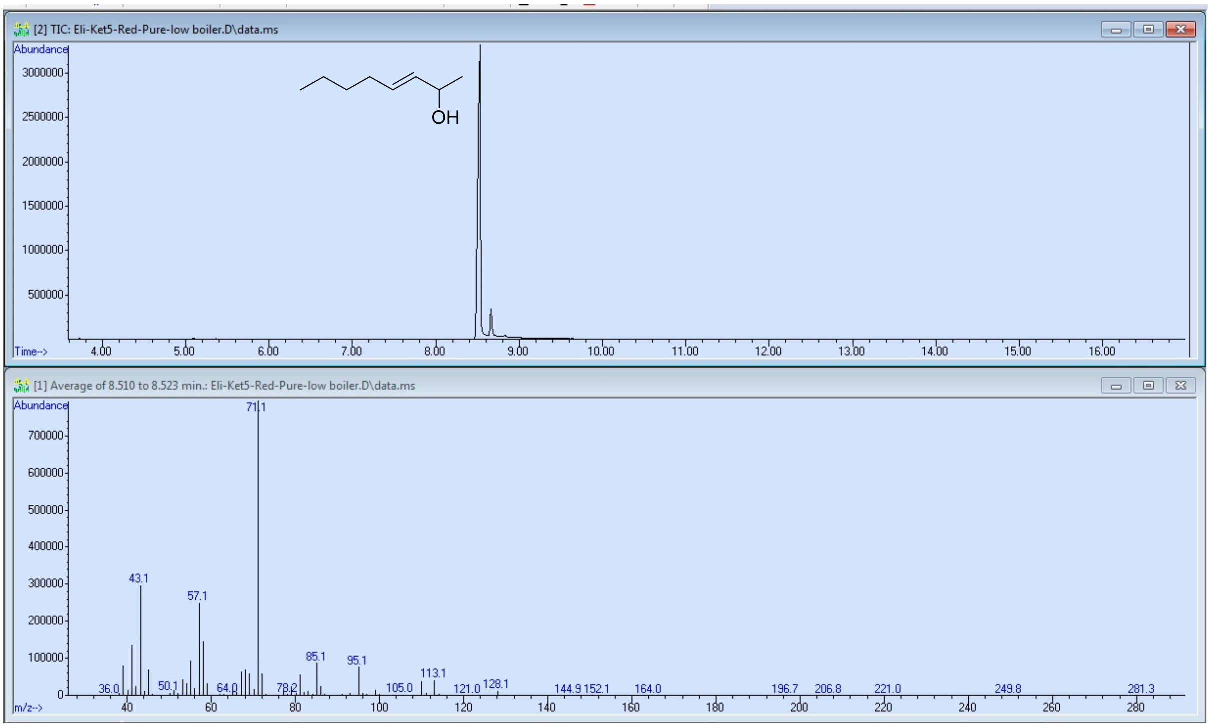

Figure S23. GC-MS chromatogram of (*E*)-oct-3-en-2-ol (**1a**) (signal at 8.7 min is related to the impurity in the substrate)

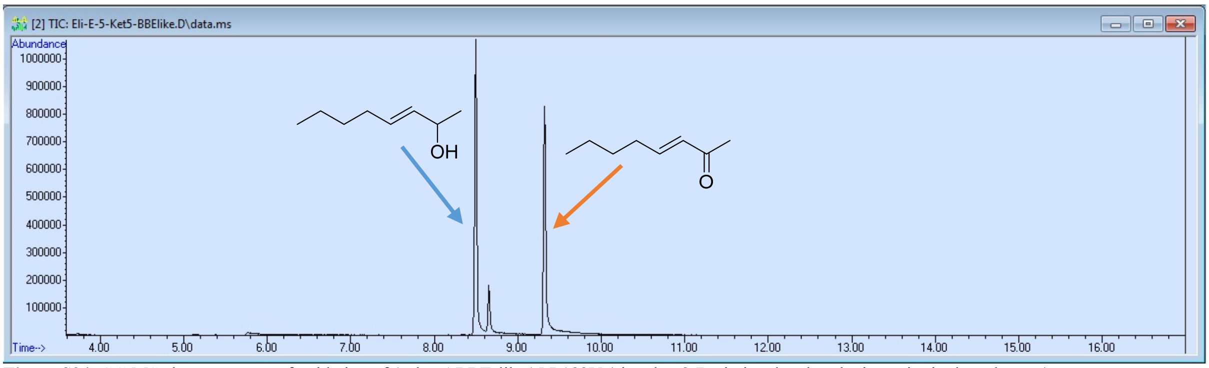

Figure S24. GC-MS chromatogram of oxidation of **1a** by *At*BBE-like15 L182V (signal at 8.7 min is related to the impurity in the substrate)

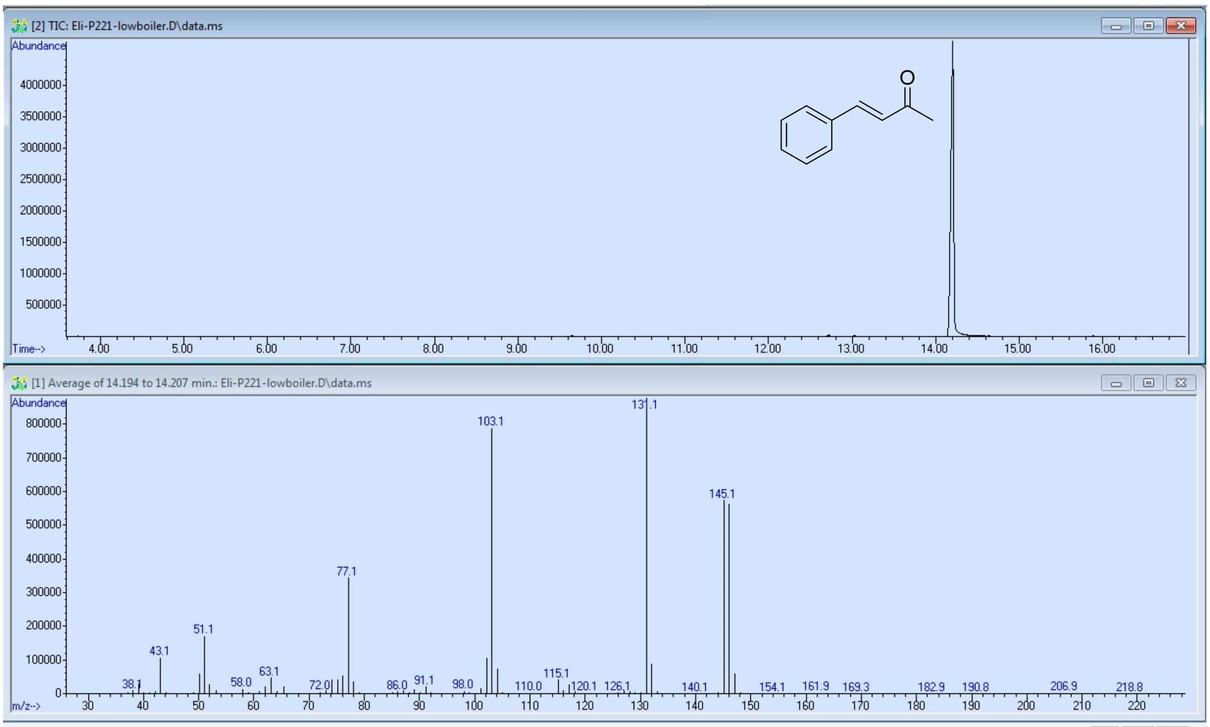

Figure S25. GC-MS chromatogram of (*E*)-4-phenylbut-3-en-2-one (**2b**)

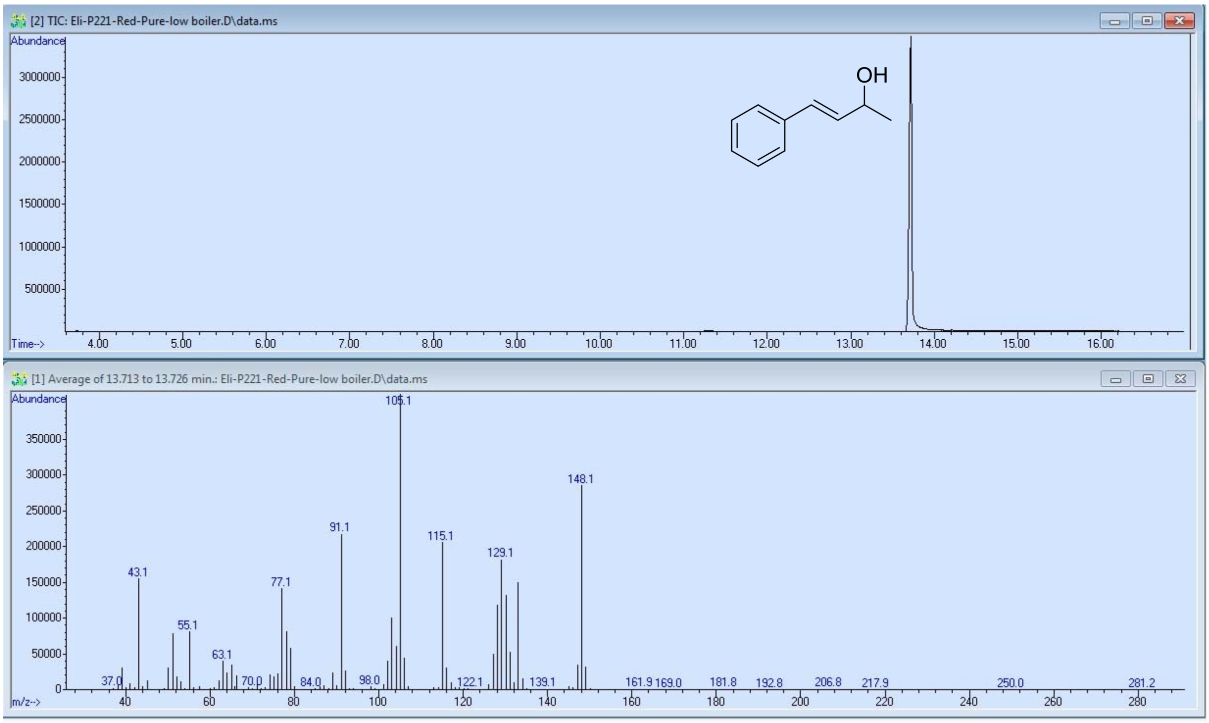

Figure S26. GC-MS chromatogram of (*E*)-4-phenylbut-3-en-2-ol (**2a**)

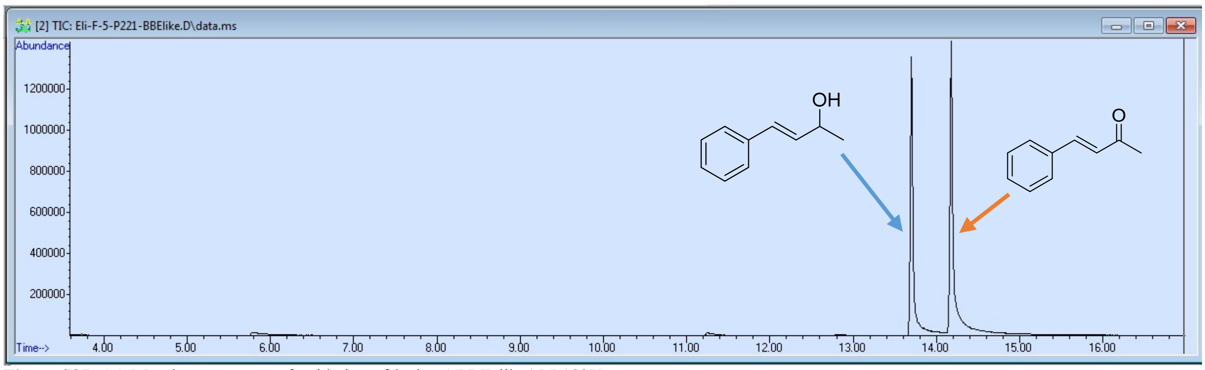

Figure S27. GC-MS chromatogram of oxidation of **2a** by *At*BBE-like15 L182V

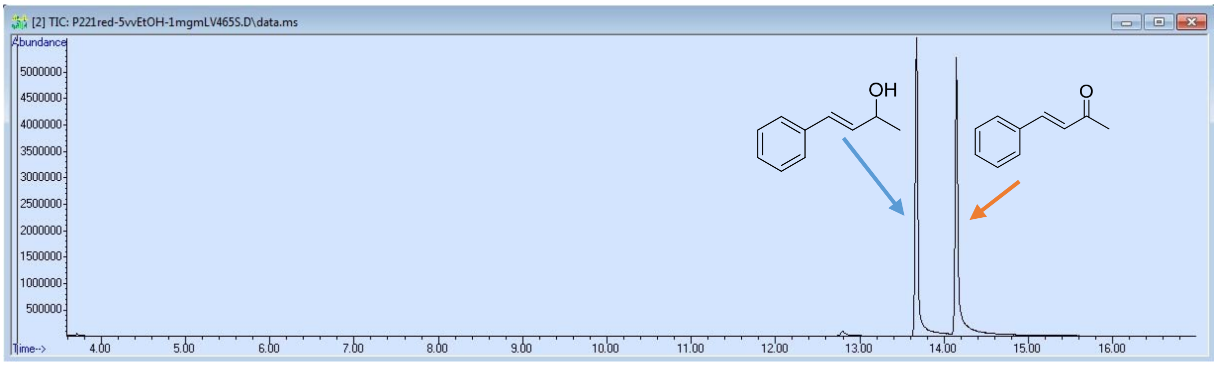

Figure S28. GC-MS chromatogram of oxidation of **2a** by HMFO V465S in the presence of 5% v/v ethanol

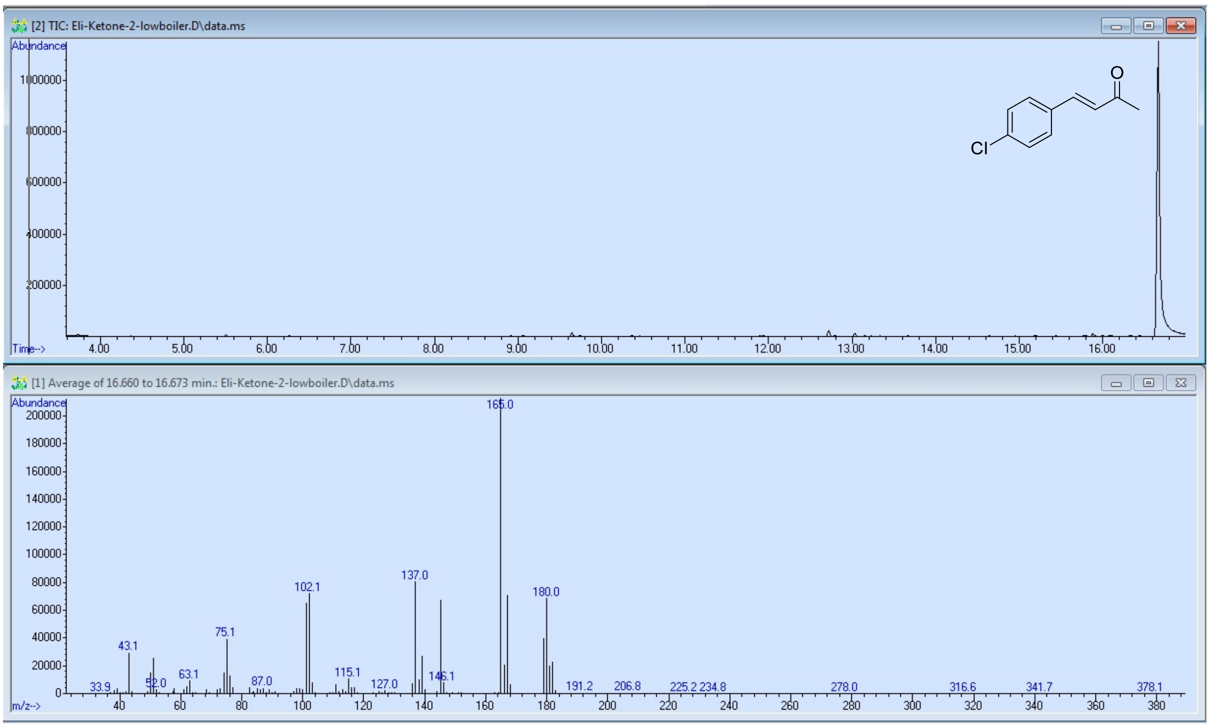

Figure S29. GC-MS chromatogram of (*E*)-4-(4-chlorophenyl)but-3-en-2-one (**3b**)

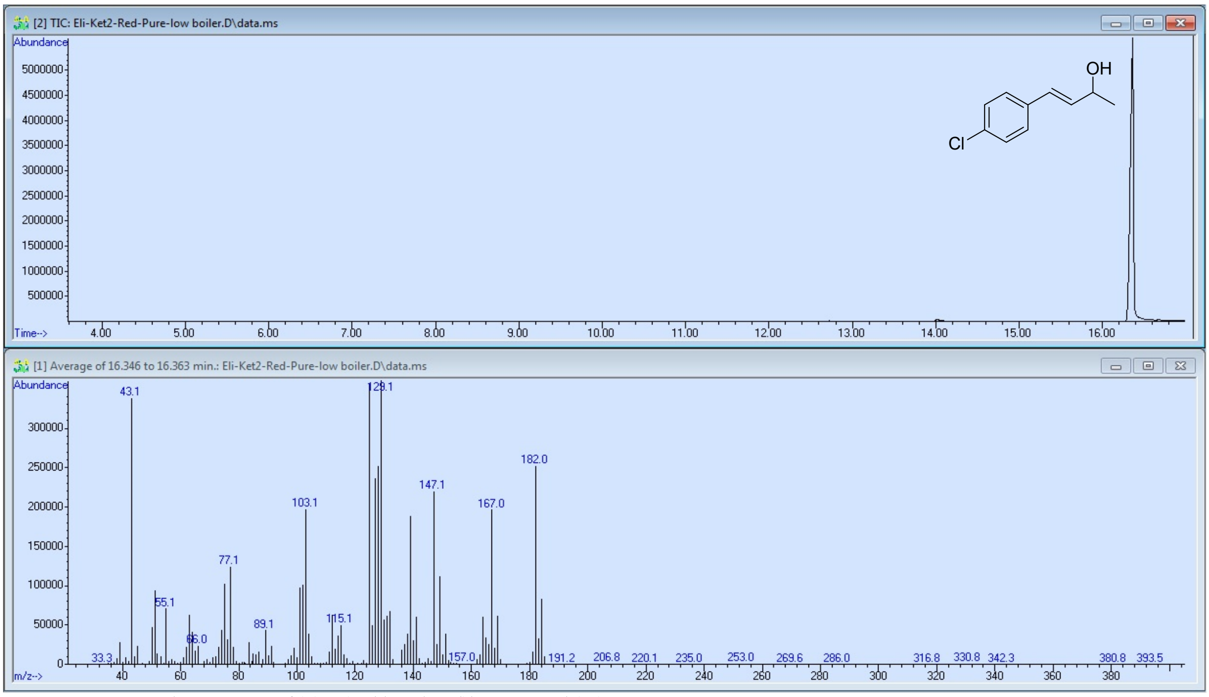

Figure S30. GC-MS chromatogram of (*E*)-4-(4-chlorophenyl)but-3-en-2-ol (**3a**)

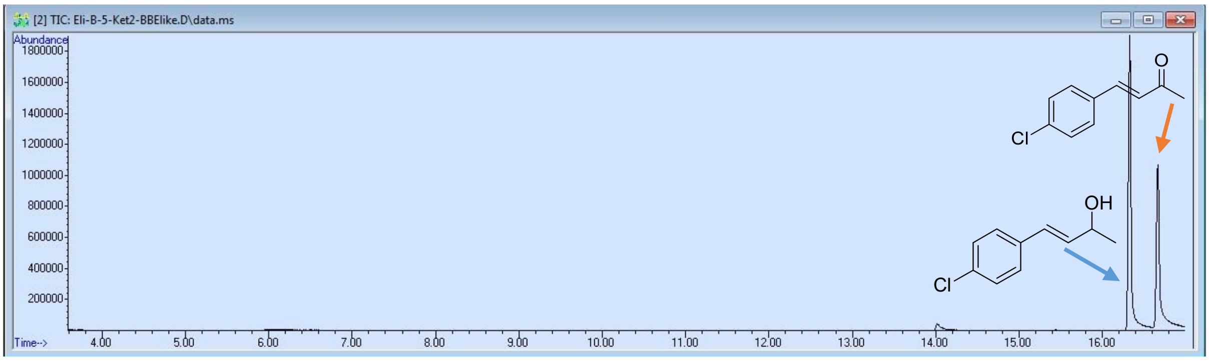

Figure S31. GC-MS chromatogram of oxidation of **3a** by *At*BBE-15like L182V

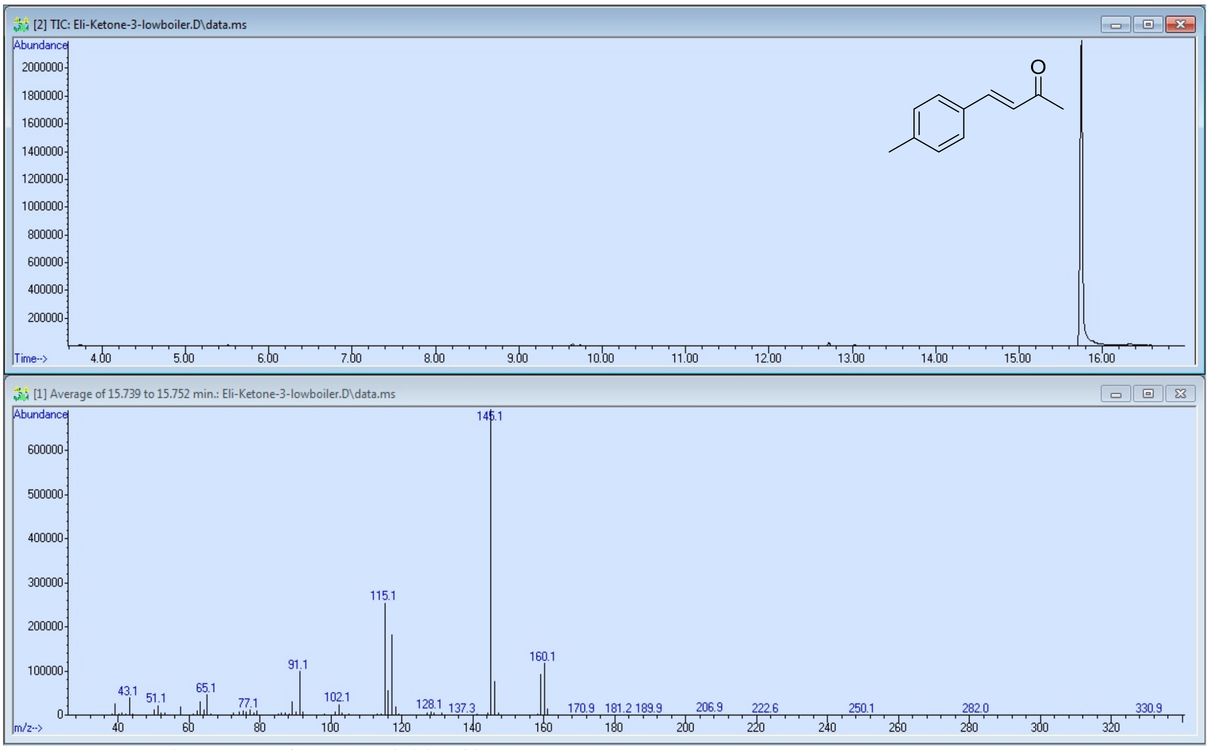

Figure S32. GC-MS chromatogram of (*E*)-4-(4-methylphenyl)but-3-en-2-one (**4b**)

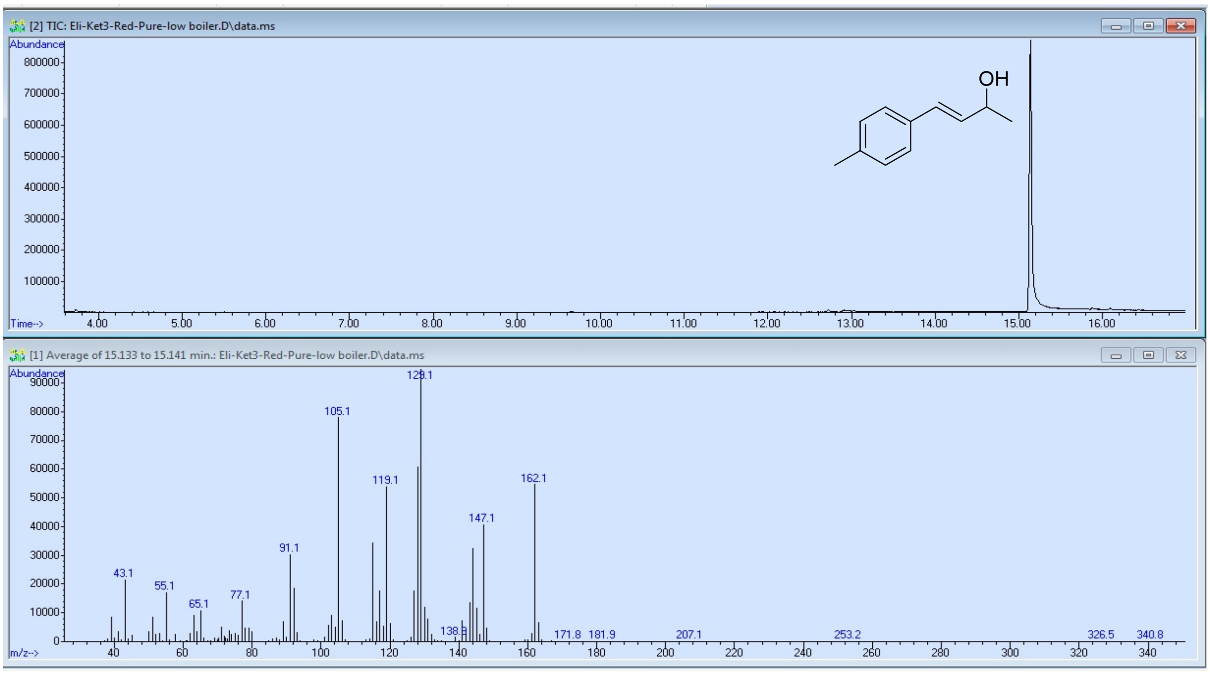

Figure S33. GC-MS chromatogram of (*E*)-4-(4-methylphenyl)but-3-en-2-ol (**4a**)

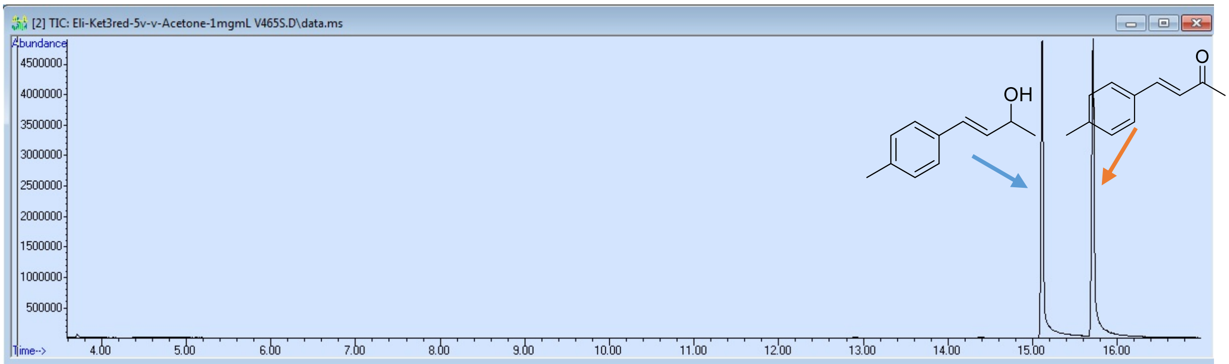

Figure S34. GC-MS of oxidation of **4a** by HMFO V465S in the presence of 5% v/v acetone

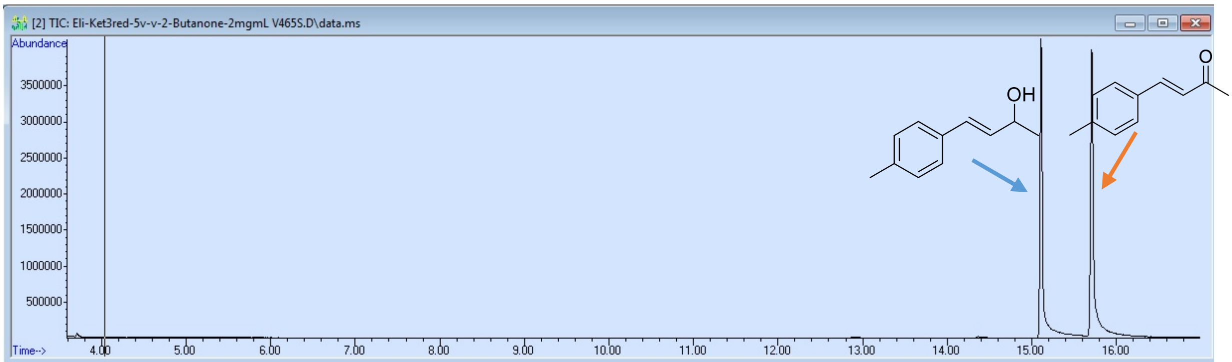

Figure S35. GC-MS of oxidation of **4a** by HMFO V465S in the presence of 5% v/v 2-butanone

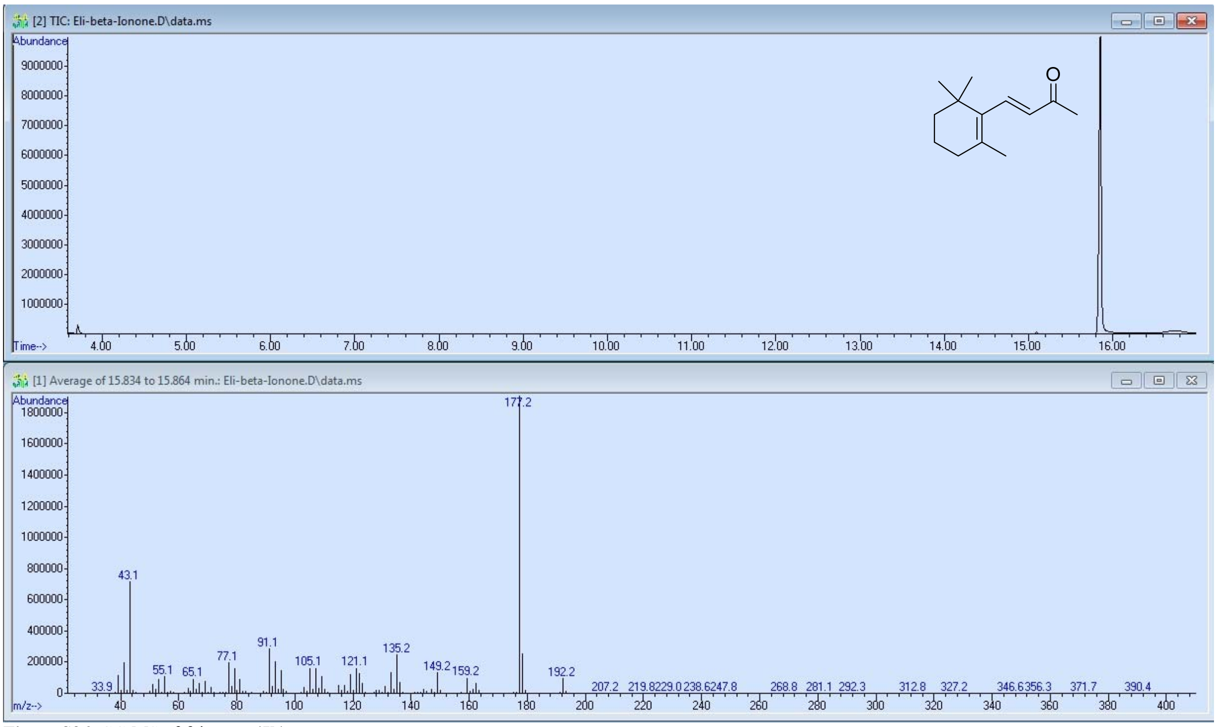

Figure S36. GC-MS of β-ionone (**5b**)

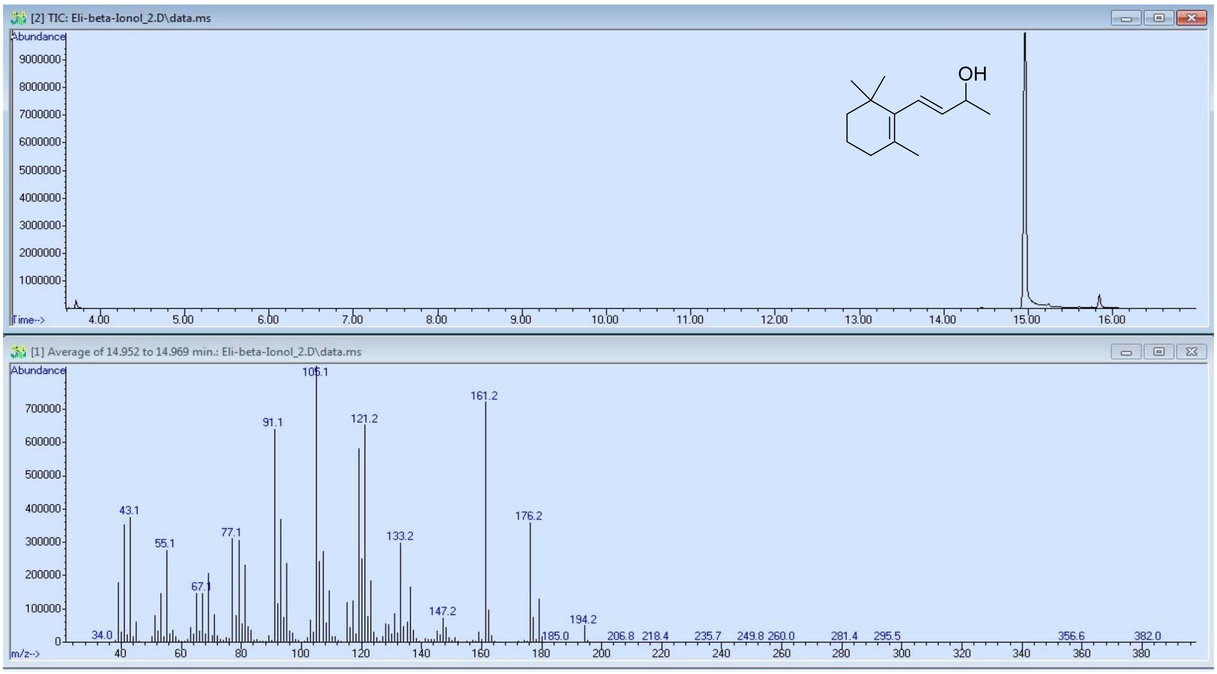

Figure S37. GC-MS of β-ionol (**5a**)

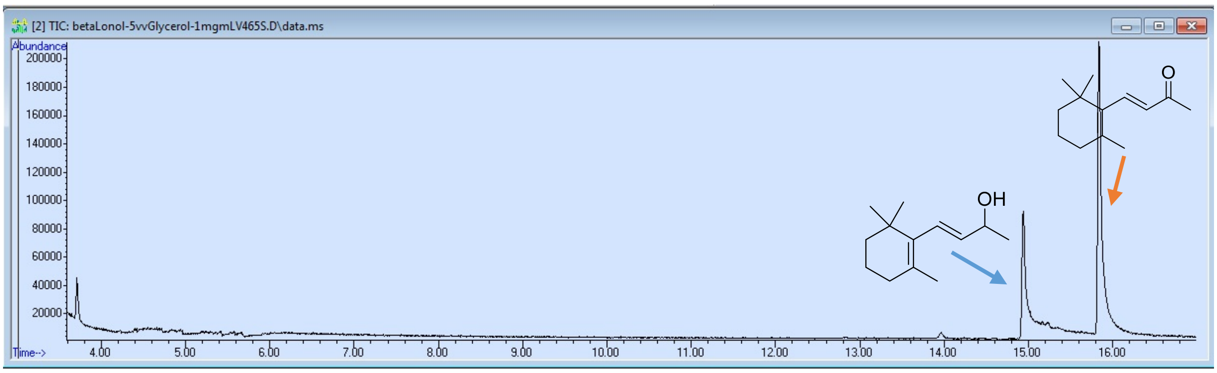

Figure S38. GC-MS chromatogram of oxidation of **5a** by HMFO V465S in the presence of 5% v/v glycerol

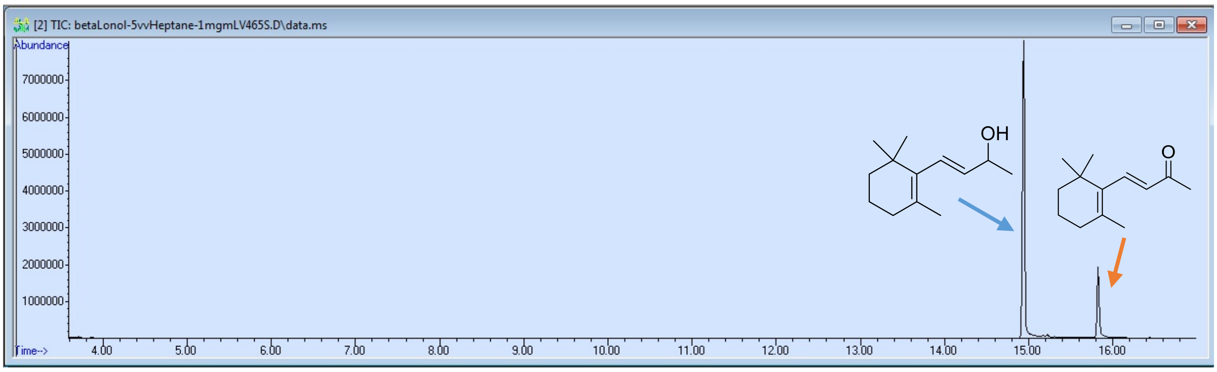

Figure S39. GC-MS chromatogram of oxidation of **5a** by HMFO V465S in the presence of 5% v/v *n*-heptane

4.3. HPLC chromatograms

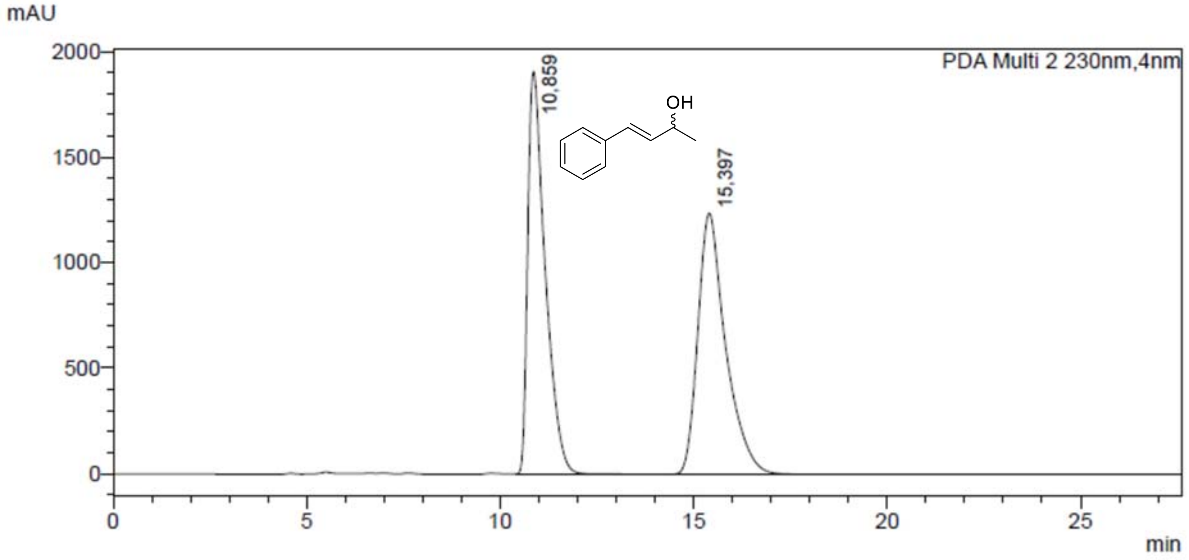

<Peak Table>

PDA Ch2 230nm

Peak#	Ret. Time	Area	Height	Name	Area%
1	10,859	59698701	1905718		49,207
2	15,397	61623196	1237443		50,793
Total		121321897	3143161		100,000

Figure S40. HPLC chromatogram of *rac*-**2a** on a chiral phase

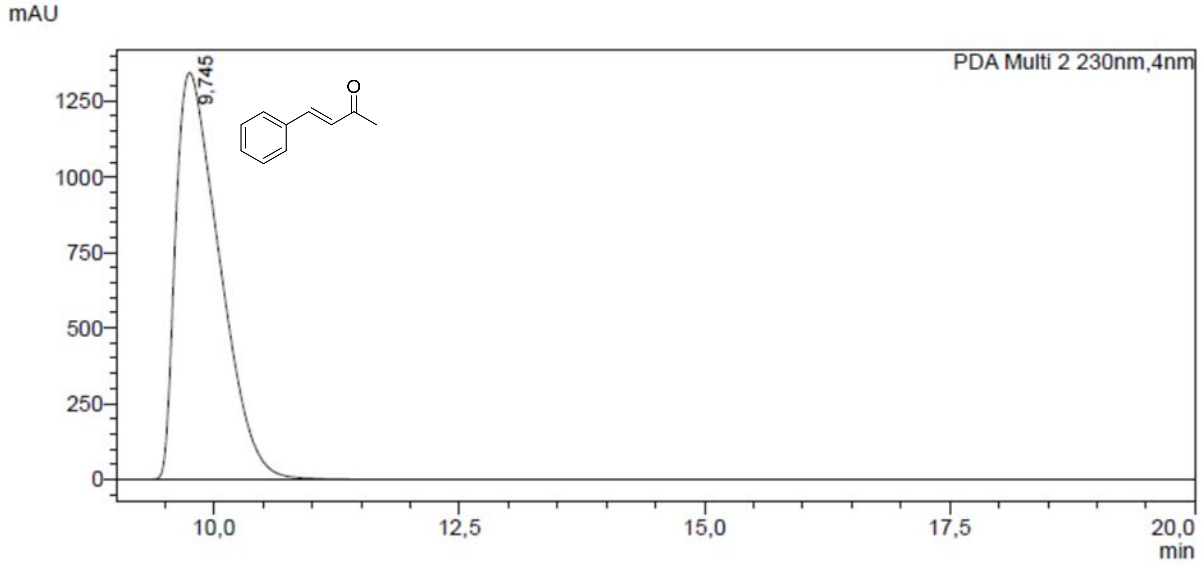

Figure S41. HPLC chromatogram of **2b** on a chiral phase

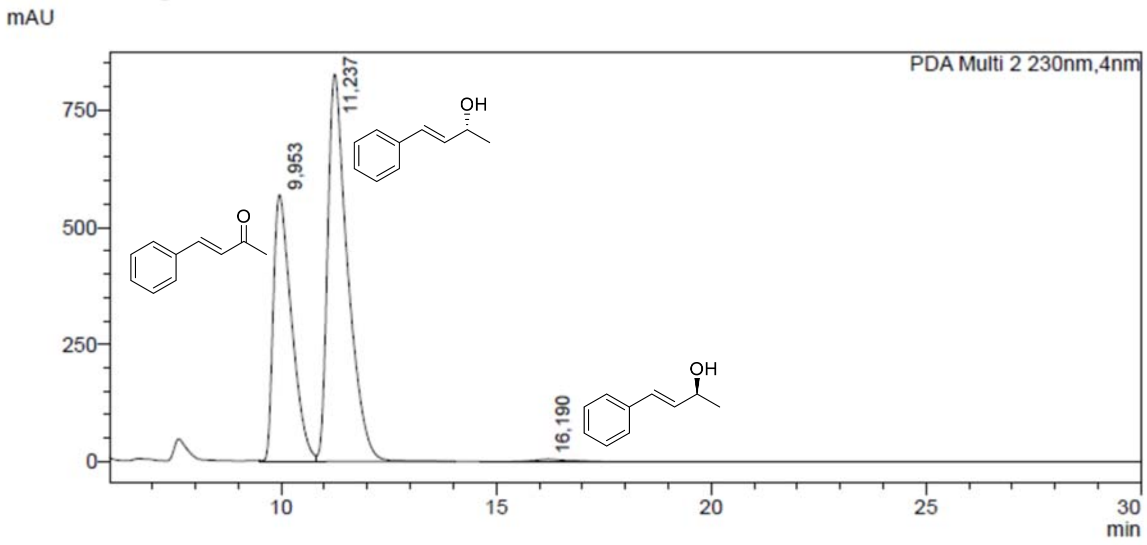

<Peak Table>

PDA Ch2 230nm

Peak#	Ret. Time	Area	Height	Name	Area%
1	9,953	16817015	569665		38,428
2	11,237	26697668	827231		61,005
3	16,190	248243	4544		0,567
Total		43762926	1401440		100,000

Figure S42. HPLC chromatogram (chiral phase) of oxidation of **2a** by HMFO V465S in the presence of 5% v/v DMSO

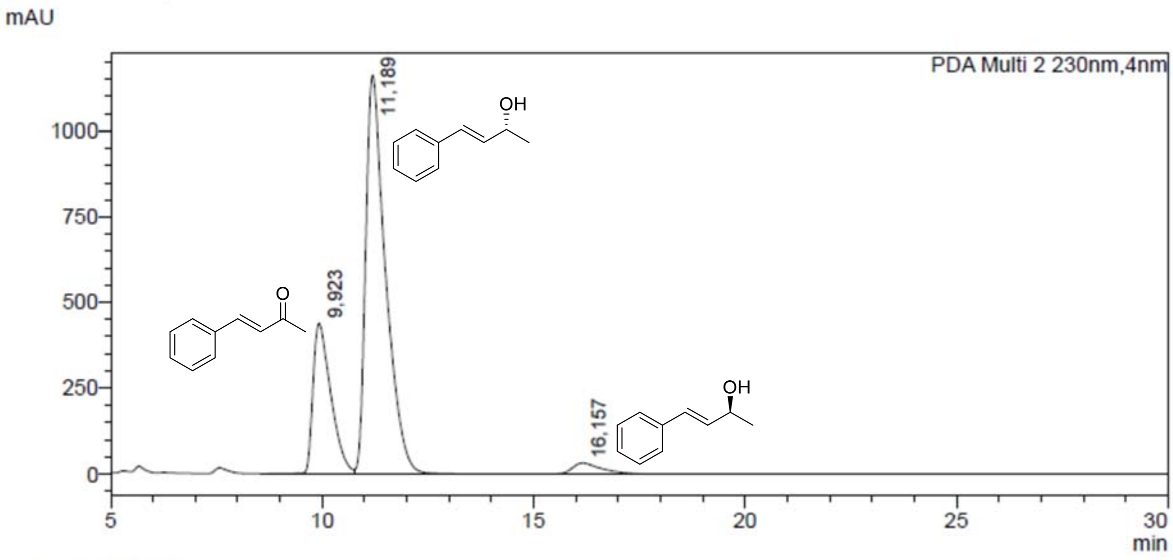

<Peak Table>

PDA Ch2 230nm

Peak#	Ret. Time	Area	Height	Name	Area%
1	9,923	12897639	437917		24,631
2	11,189	37980419	1161605		72,531
3	16,157	1486045	31593		2,838
Total		52364104	1631115		100,000

Figure S43. HPLC chromatogram (chiral phase) of oxidation of **2a** by HMFO V465S in the presence of 5% v/v ethanol

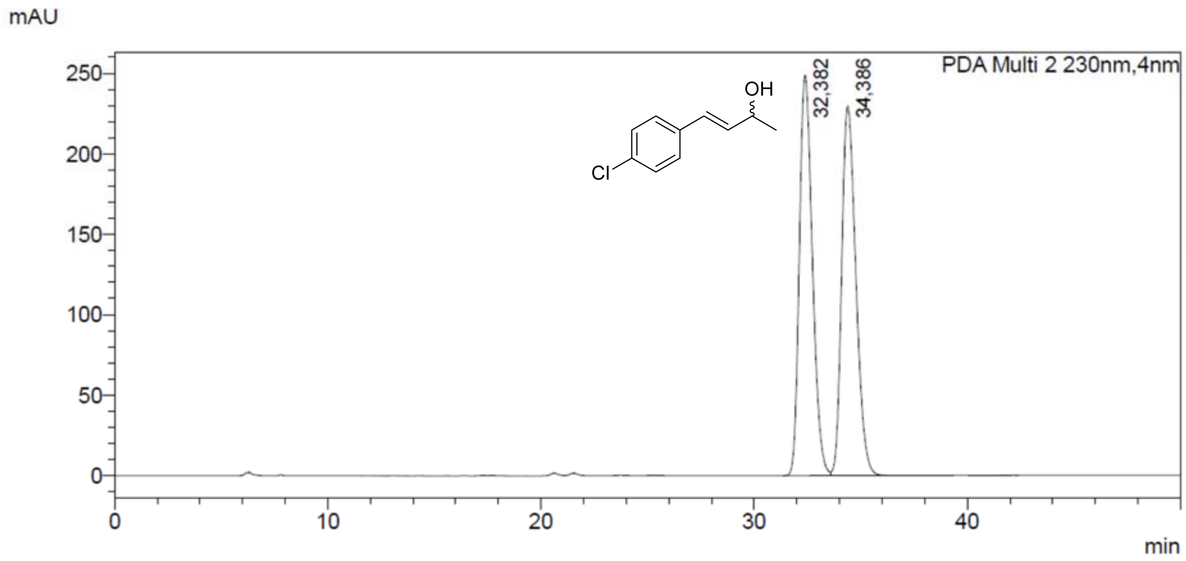

<Peak Table>

PDA Ch2 230nm

Peak#	Ret. Time	Area	Height	Name	Area%
1	32,382	10584056	248824		49,904
2	34,386	10624640	229547		50,096
Total		21208695	478371		100,000

Figure S44. HPLC chromatogram of *rac*-**3a** (chiral phase)

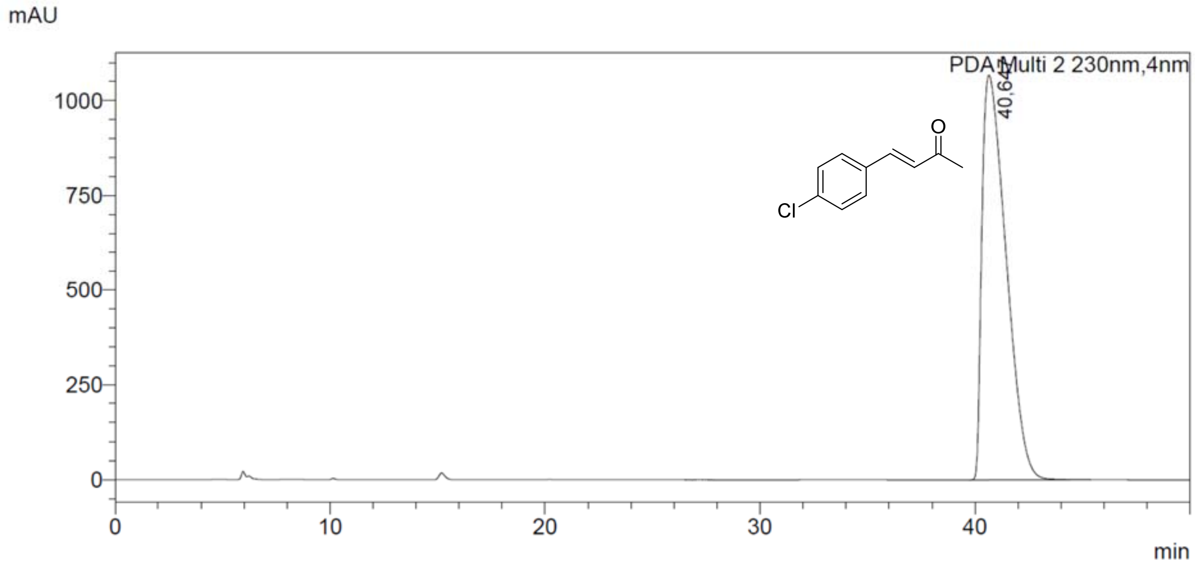

Figure S45. HPLC chromatogram of **3b** (chiral phase)

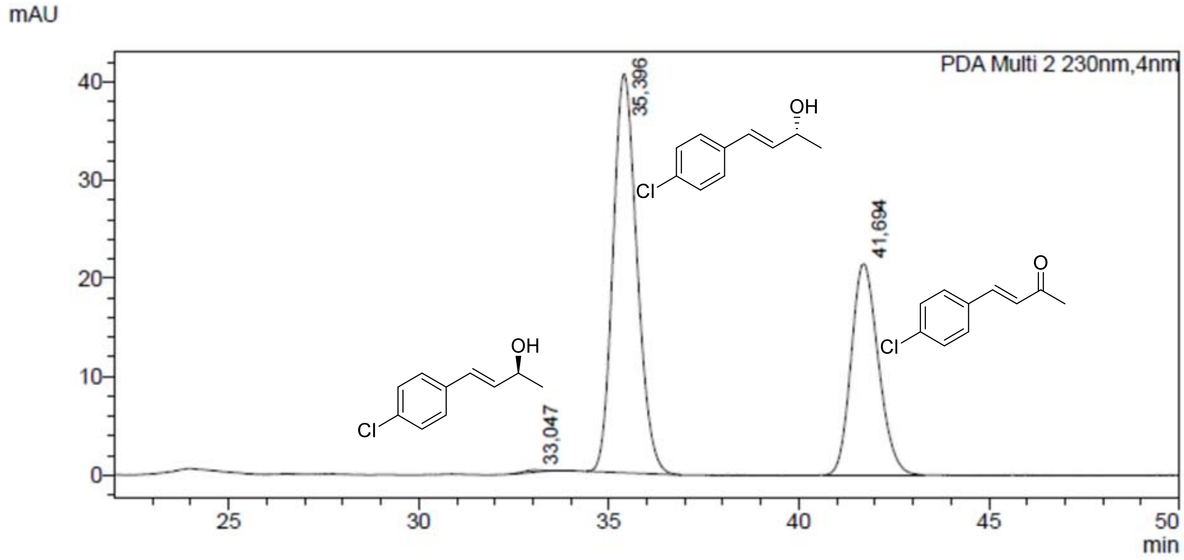

<Peak Table>

PDA Ch2 230nm

Peak#	Ret. Time	Area	Height	Name	Area%
1	33,047	7131	230		0,250
2	35,396	1779322	40539		62,339
3	41,694	1067795	21469		37,411
Total		2854248	62238		100,000

Figure S46. HPLC chromatogram (chiral phase) of oxidation of **3a** by HMFO V465S in the presence of 5% v/v glycerol

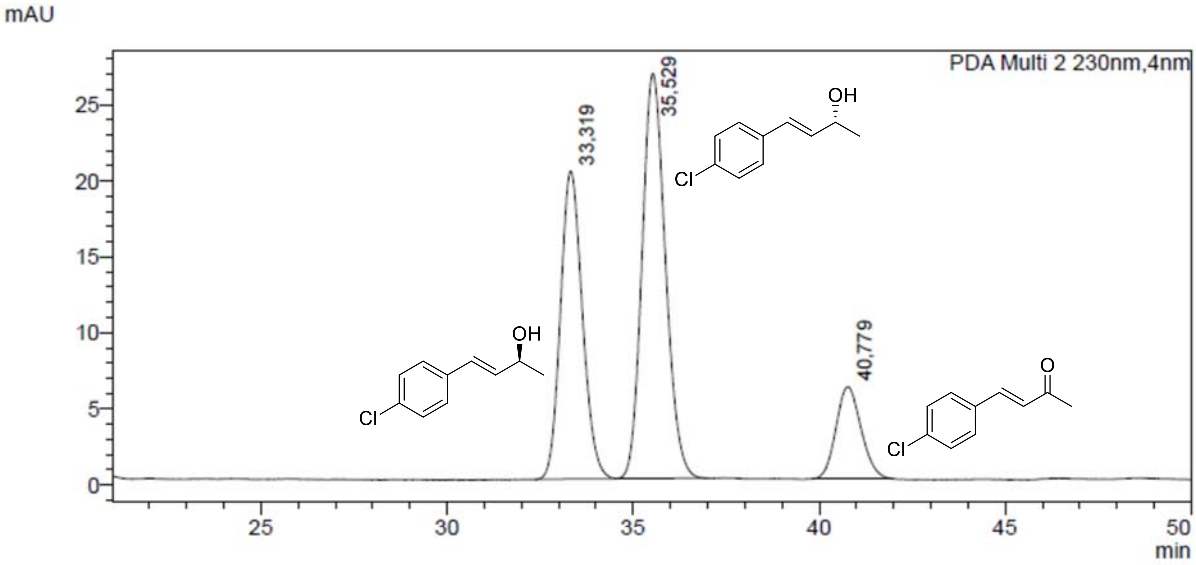

<Peak Table>

PDA Ch2 230nm

Peak#	Ret. Time	Area	Height	Name	Area%
1	33,319	830796	20299		36,473
2	35,529	1162363	26638		51,030
3	40,779	284649	6025		12,497
Total		2277808	52962		100,000

Figure S47. HPLC chromatogram (chiral phase) of oxidation of **3a** by *At*BBE-like15 L182V/L178V/I184

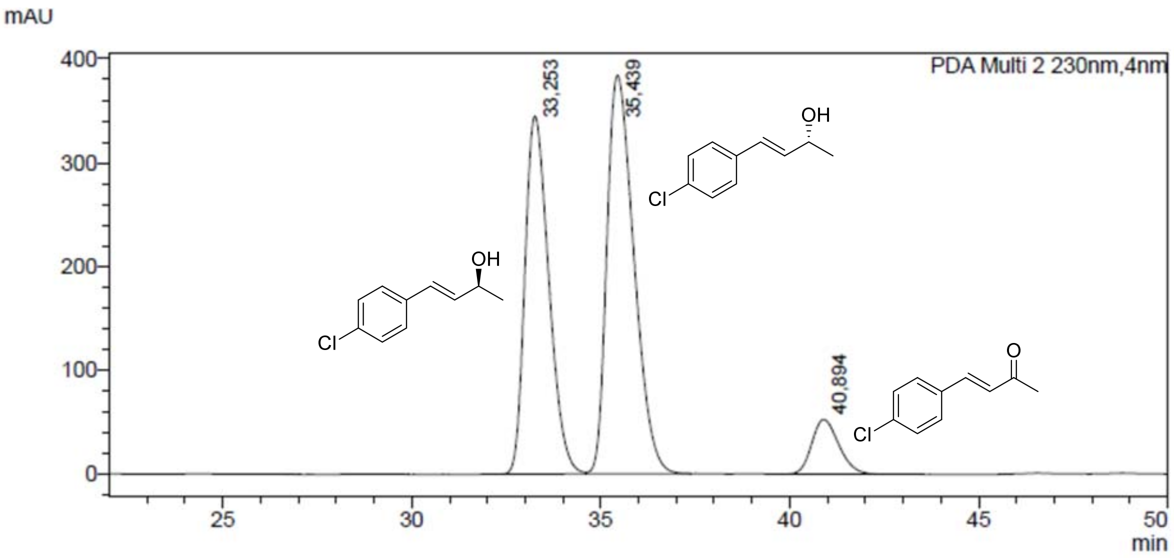

<Peak Table>

PDA Ch2 230nm

Peak#	Ret. Time	Area	Height	Name	Area%
1	33,253	15152098	344351		41,128
2	35,439	19063363	383121		51,745
3	40,894	2625574	52683		7,127
Total		36841035	780155		100,000

Figure S48. HPLC chromatogram (chiral phase) of oxidation of **3a** by HMFO wild type

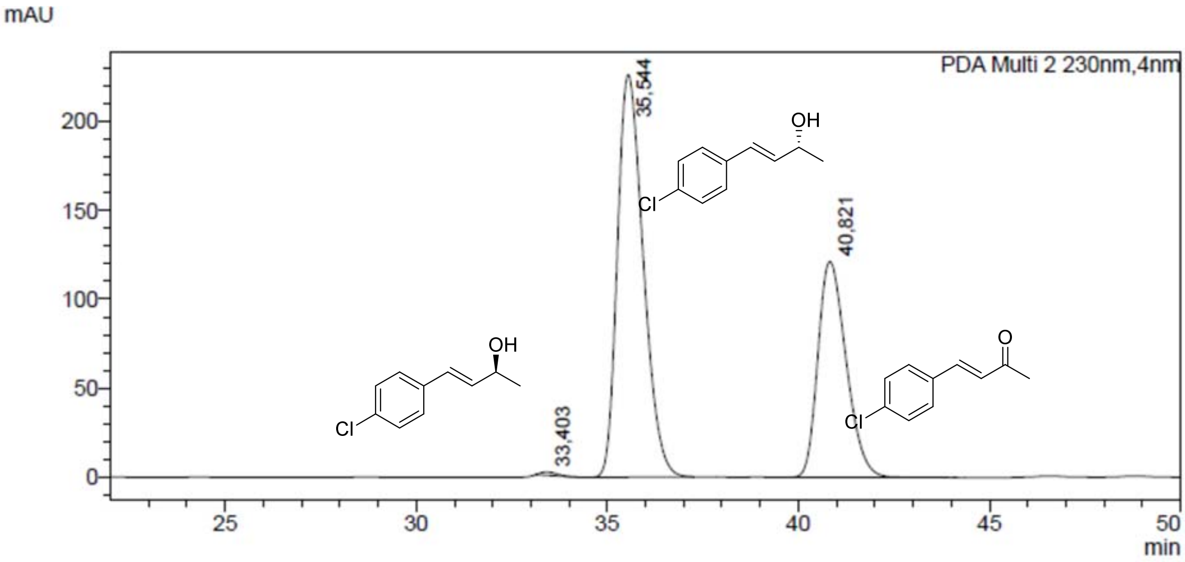

<Peak Table>

PDA Ch2 230nm

Peak#	Ret. Time	Area	Height	Name	Area%
1	33,403	54178	1899		0,320
2	35,544	10640292	226123		62,869
3	40,821	6230203	121346		36,811
Total		16924674	349368		100,000

Figure S49. HPLC chromatogram (chiral phase) of oxidation of **3a** by HMFO V465S

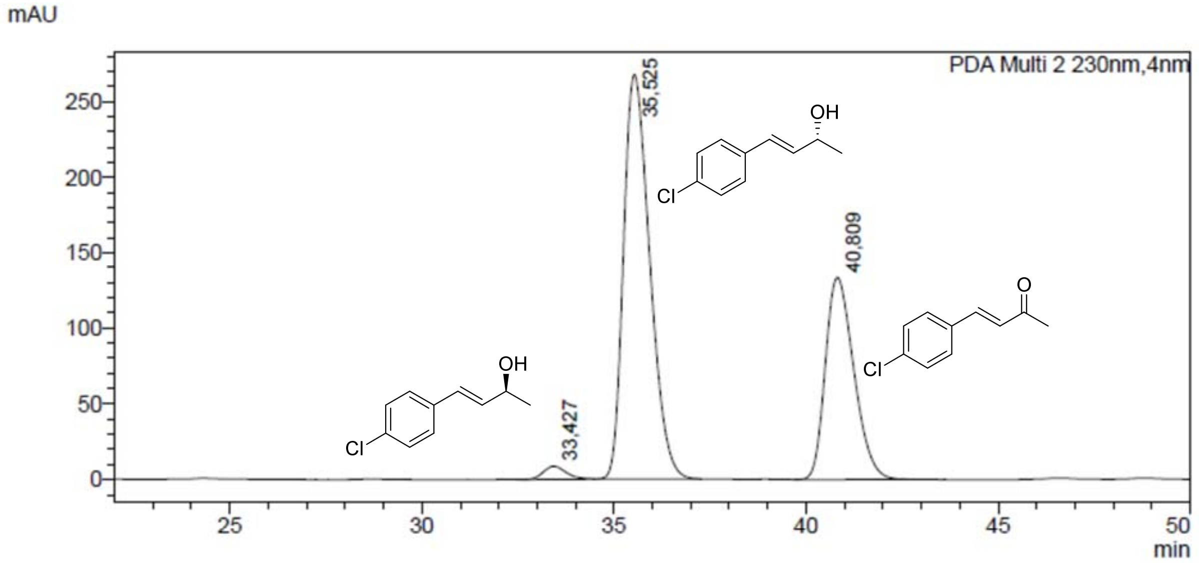

<Peak Table>

PDA Ch2 230nm

Peak#	Ret. Time	Area	Name	Area%
1	33,427	368176		1,844
2	35,525	12704696		63,644
3	40,809	6889150		34,511
Total		19962022		100,000

Figure S50. HPLC chromatogram (chiral phase) of oxidation of **3a** by HMFO V465T

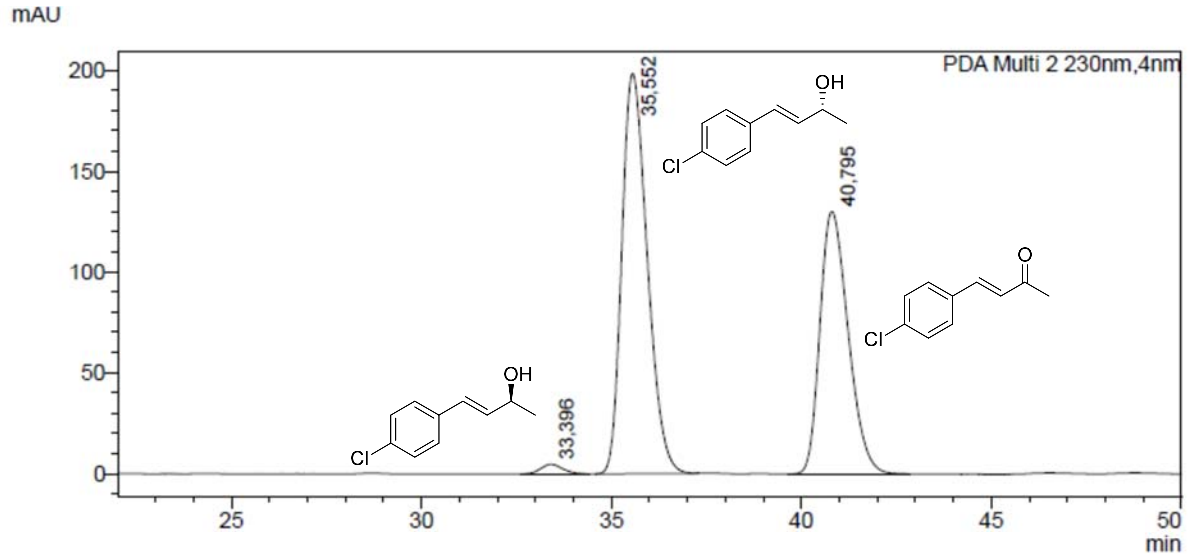

<Peak Table>

PDA Ch2 230nm

Peak#	Ret. Time	Area	Height	Name	Area%
1	33,396	191641	4738		1,187
2	35,552	9232591	198254		57,197
3	40,795	6717371	130276		41,615
Total		16141603	333268		100,000

Figure S51. HPLC chromatogram (chiral phase) of oxidation of **3a** by HMFO V465T/ W466H

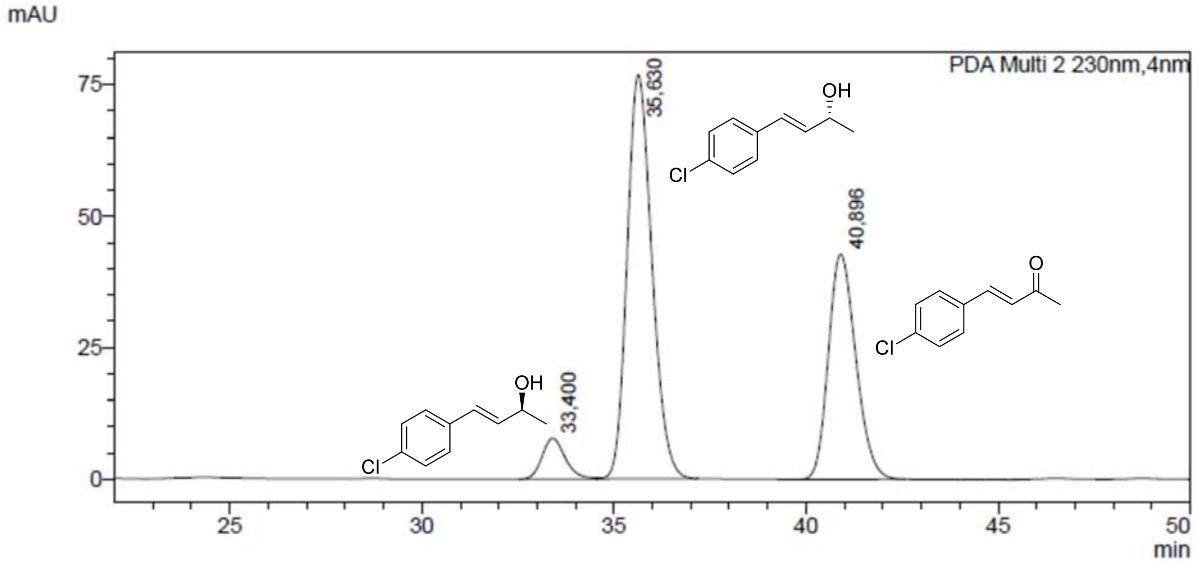

<Peak Table>

PDA Ch2 230nm

Peak#	Ret. Time	Area	Height	Name	Area%
1	33,400	325081	7751		5,507
2	35,630	3448573	76821		58,417
3	40,896	2129676	42904		36,076
Total		5903330	127476		100,000

Figure S52. HPLC chromatogram (chiral phase) of oxidation of **3a** by HMFO V465T/W466F

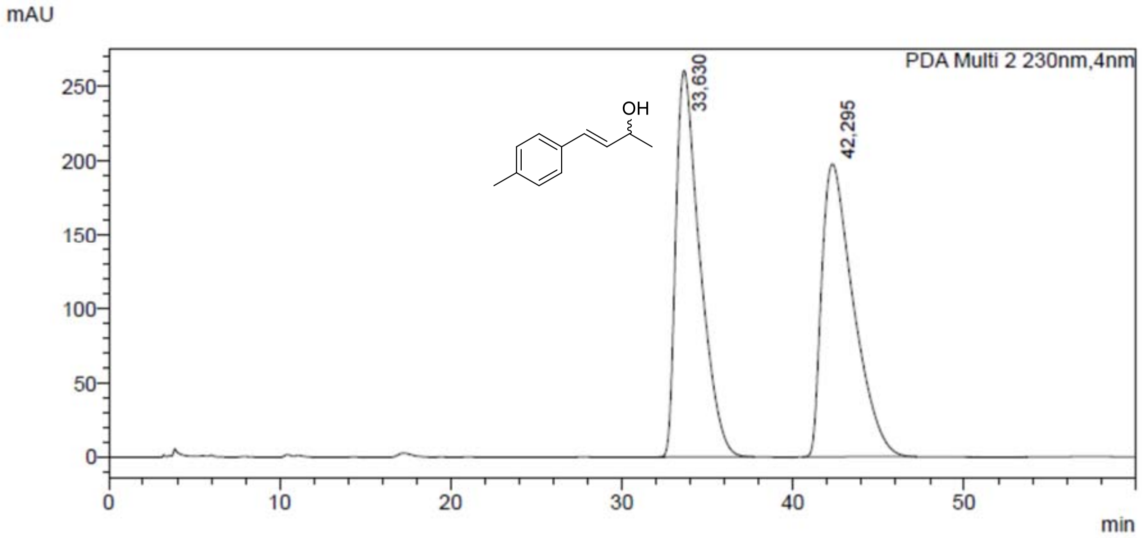

<Peak Table>

PDA Ch2 230nm

Peak#	Ret. Time	Area	Height	Name	Area%
1	33,630	25354077	260499		49,913
2	42,295	25442770	197757		50,087
Total		50796847	458256		100,000

Figure S53. HPLC chromatogram of *rac*-**4a** (chiral phase)

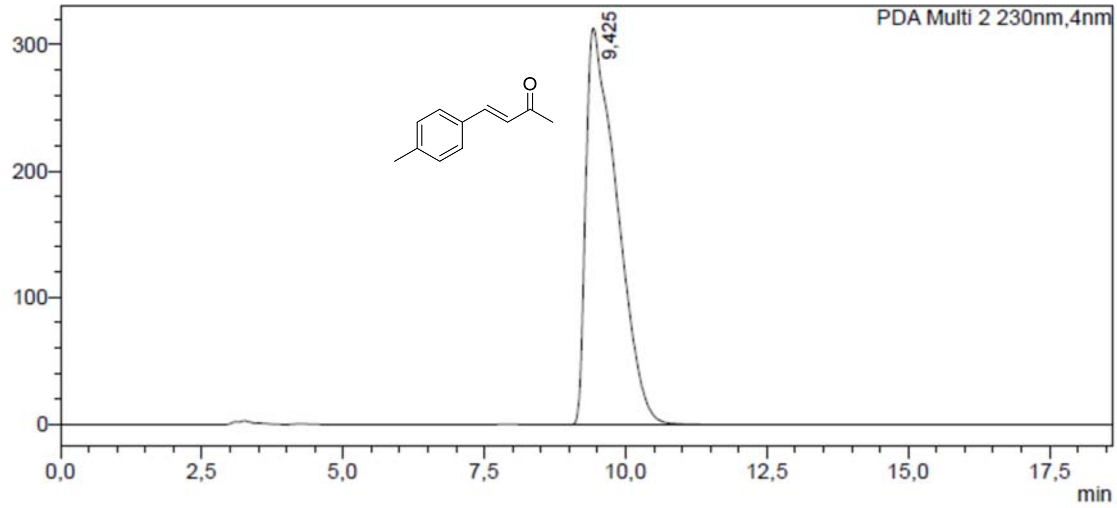

Figure S54. HPLC chromatogram of **4b** (chiral phase)

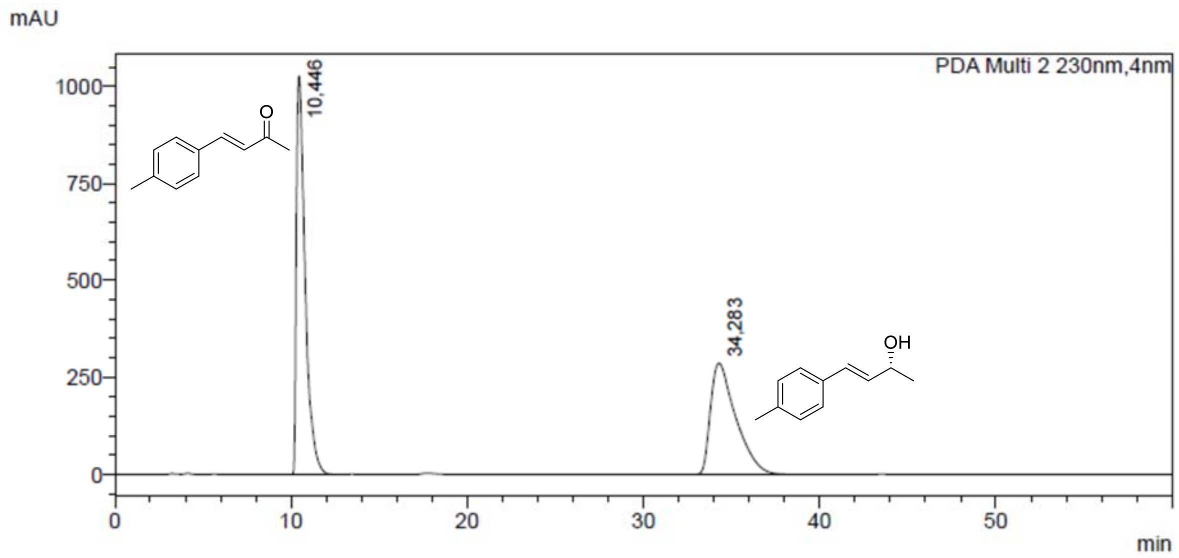

<Peak Table>

PDA Ch2 230nm

Peak#	Ret. Time	Area	Height	Name	Area%
1	10,446	35531695	1025951		55,515
2	34,283	28471954	284966		44,485
Total		64003649	1310917		100,000

Figure S55. HPLC chromatogram (chiral phase) of oxidation of **4a** by HMFO V465S in the presence of 5% v/v ethanol

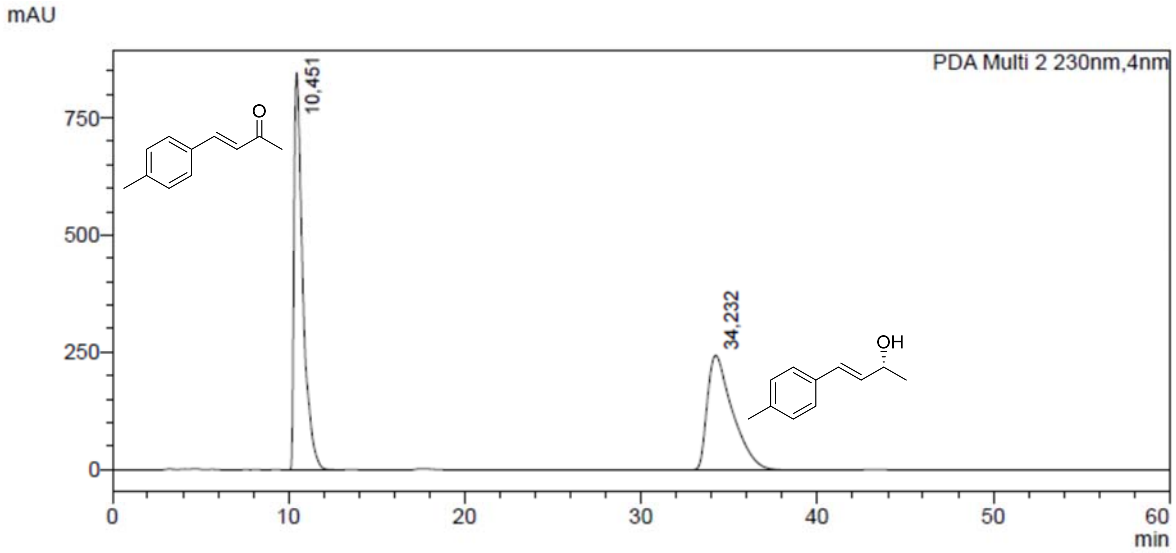

<Peak Table>

PDA Ch2 230nm

Peak#	Ret. Time	Area	Height	Name	Area%
1	10,451	29263727	845717		54,714
2	34,232	24221592	243375		45,286
Total		53485319	1089092		100,000

Figure S56. HPLC chromatogram (chiral phase) of oxidation of **4a** by HMFO V465S in the presence of 5% v/v dioxane

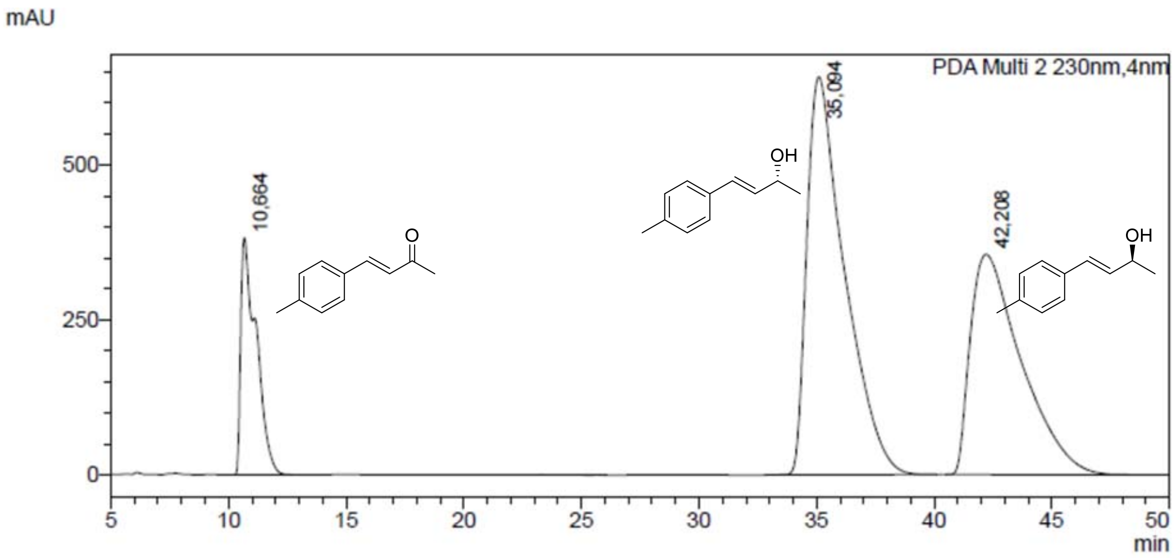

<Peak Table>

PDA Ch2 230nm

Peak#	Ret. Time	Area	Height	Name	Area%
1	10,664	17554730	382884		12,310
2	35,094	71611937	641095		50,215
3	42,208	53443620	355338		37,475
Total		142610287	1379318		100,000

Figure S57. HPLC chromatogram (chiral phase) of oxidation of **4a** by HMFO wild type

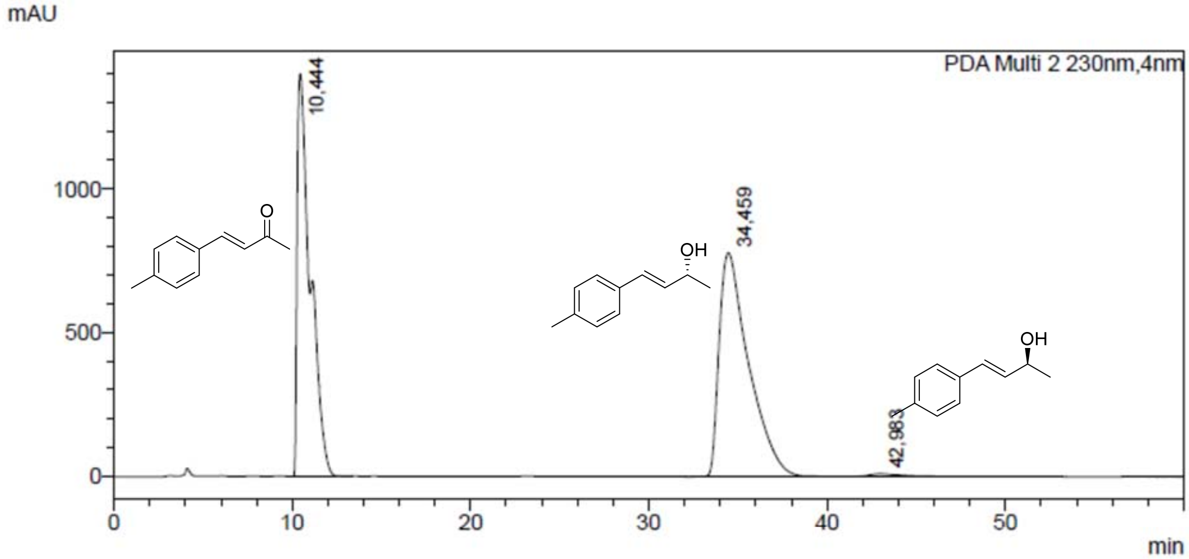

<Peak Table>

PDA Ch2 230nm

Peak#	Ret. Time	Area	Height	Name	Area%
1	10,444	72013117	1398223		44,395
2	34,459	89056673	778054		54,902
3	42,983	1140802	9042		0,703
Total		162210592	2185319		100,000

Figure S58. HPLC chromatogram (chiral phase) of oxidation of **4a** by HMFO V465S

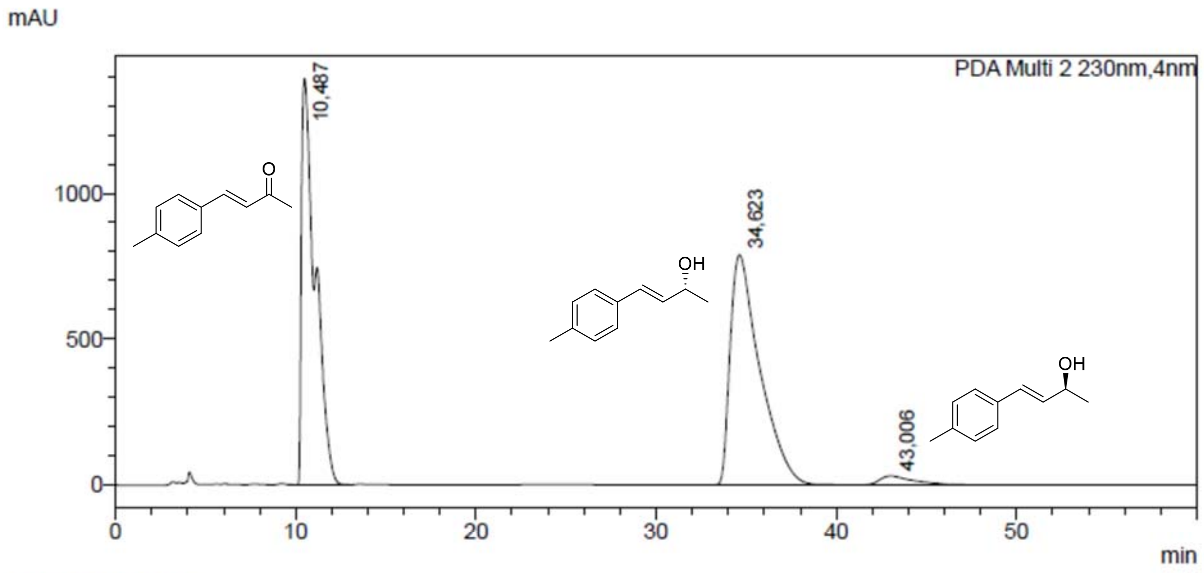

<Peak Table>

PDA Ch2 230nm

Peak#	Ret. Time	Area	Height	Name	Area%
1	10,487	74063757	1390911		44,064
2	34,623	90172666	789179		53,648
3	43,006	3846717	30322		2,289
Total		168083140	2210413		100,000

Figure S59. HPLC chromatogram (chiral phase) of oxidation of **4a** by HMFO V465T

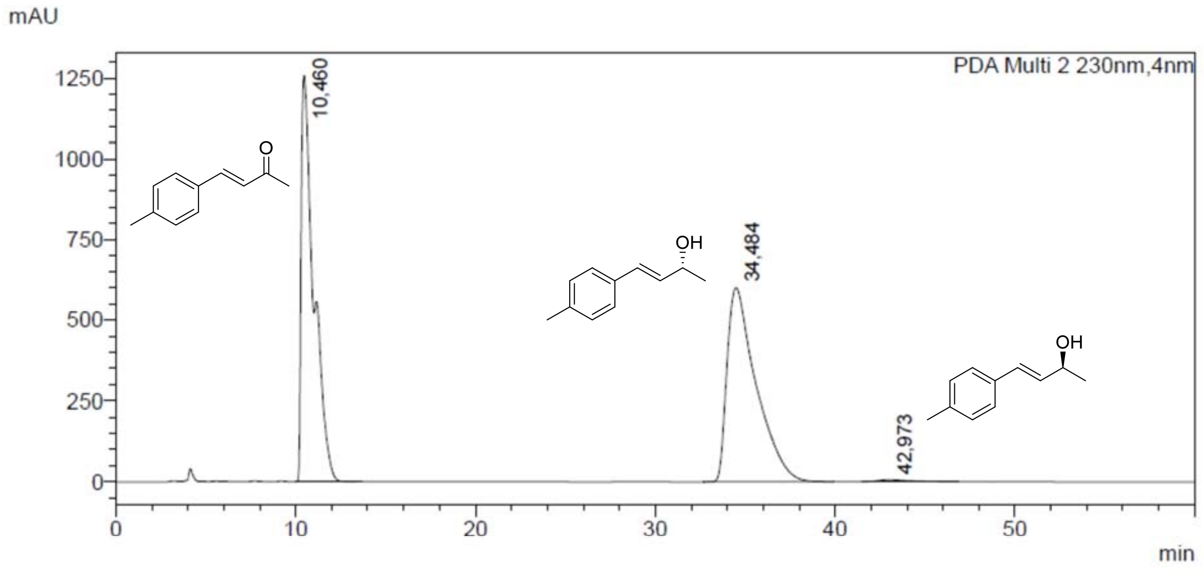

<Peak Table>

PDA Ch2 230nm

Peak#	Ret. Time	Area	Height	Name	Area%
1	10,460	61035656	1257551		47,329
2	34,484	67241954	599239		52,141
3	42,973	684126	5540		0,530
Total		128961737	1862330		100,000

Figure S60. HPLC chromatogram (chiral phase) of oxidation of **4a** by HMFO V465T/ W466H

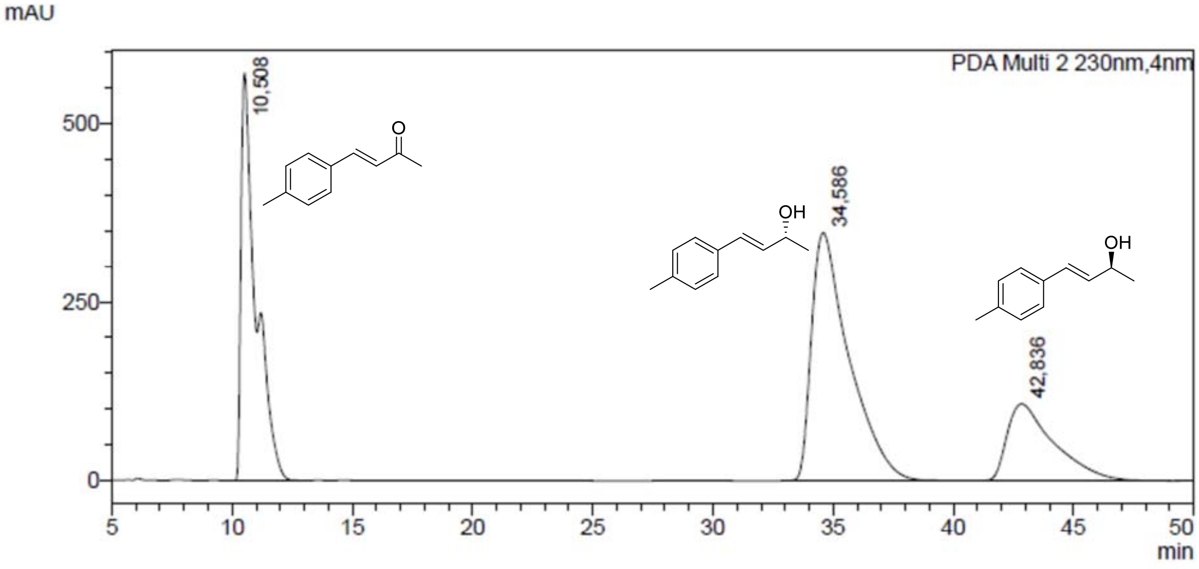

<Peak Table>

PDA Ch2 230nm

Peak#	Ret. Time	Area	Height	Name	Area%
1	10,508	24788647	570295		32,211
2	34,586	37712102	346871		49,004
3	42,836	14456548	107027		18,785
Total		76957298	1024193		100,000

Figure S61. HPLC chromatogram (chiral phase) of oxidation of **4a** by HMFO V367R/ W466F

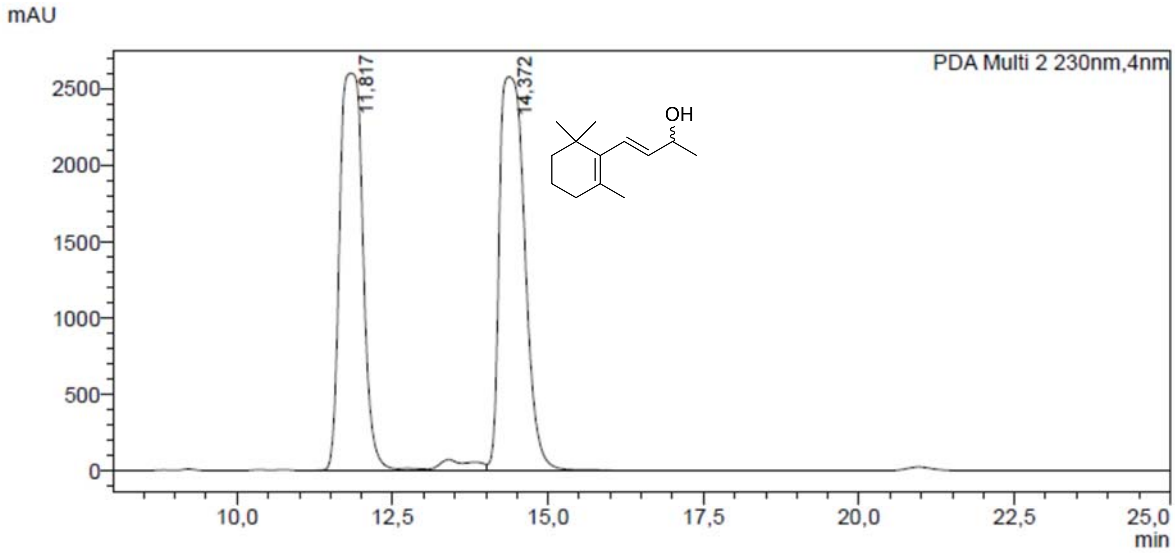

<Peak Table>

PDA Ch2 230nm

Peak#	Ret. Time	Area	Height	Name	Area%
1	11,817	70503581	2604152		49,007
2	14,372	73360357	2579478		50,993
Total		143863939	5183631		100,000

Figure S62. HPLC chromatogram of *rac*-**5a** (chiral phase)

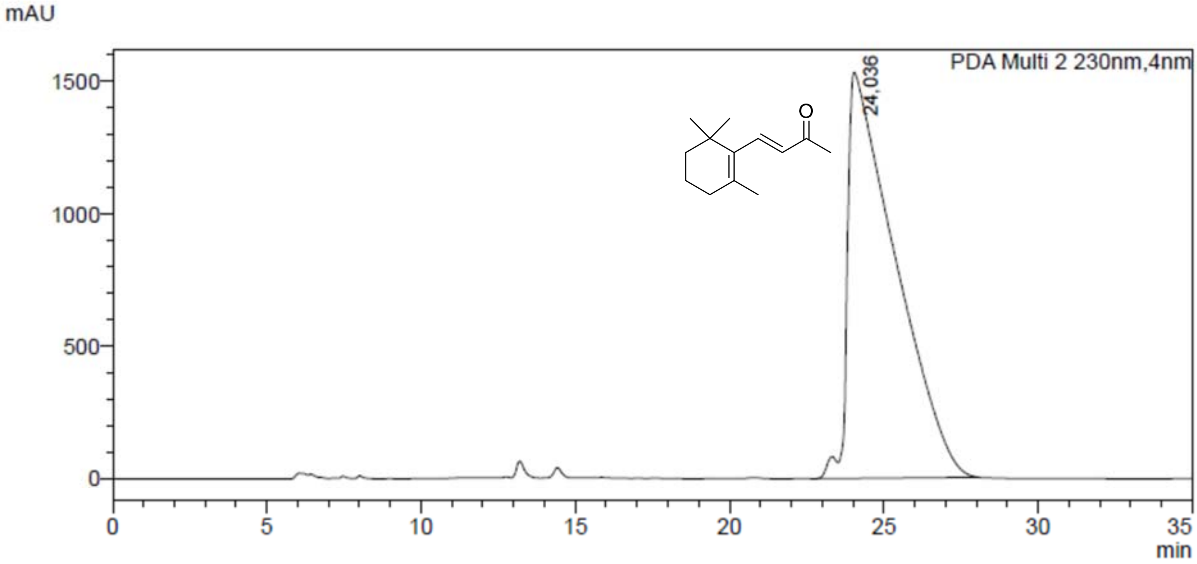

<Peak Table>

PDA Ch2 230nm

Peak#	Ret. Time	Area	Height	Conc.	Unit	Mark	Name
1	24,036	165920780	1532640	0,000		M	
Total		165920780	1532640				

Figure S63. HPLC chromatogram of **5b** (chiral phase)

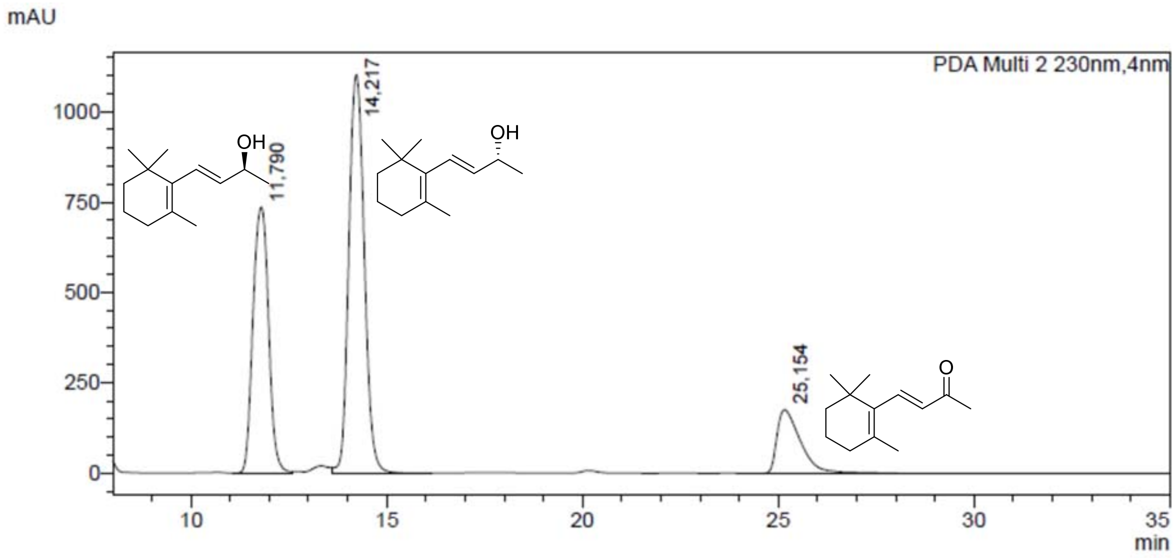

<Peak Table>

PDA Ch2 230nm

Peak#	Ret. Time	Area	Height	Name	Area%
1	11,790	20334378	735696		35,050
2	14,217	30317288	1100724		52,257
3	25,154	7364487	175647		12,694
Total		58016153	2012067		100,000

Figure S64. HPLC chromatogram (chiral phase) of oxidation of **5a** by HMFO V465S in the presence of 5% v/v isooctane

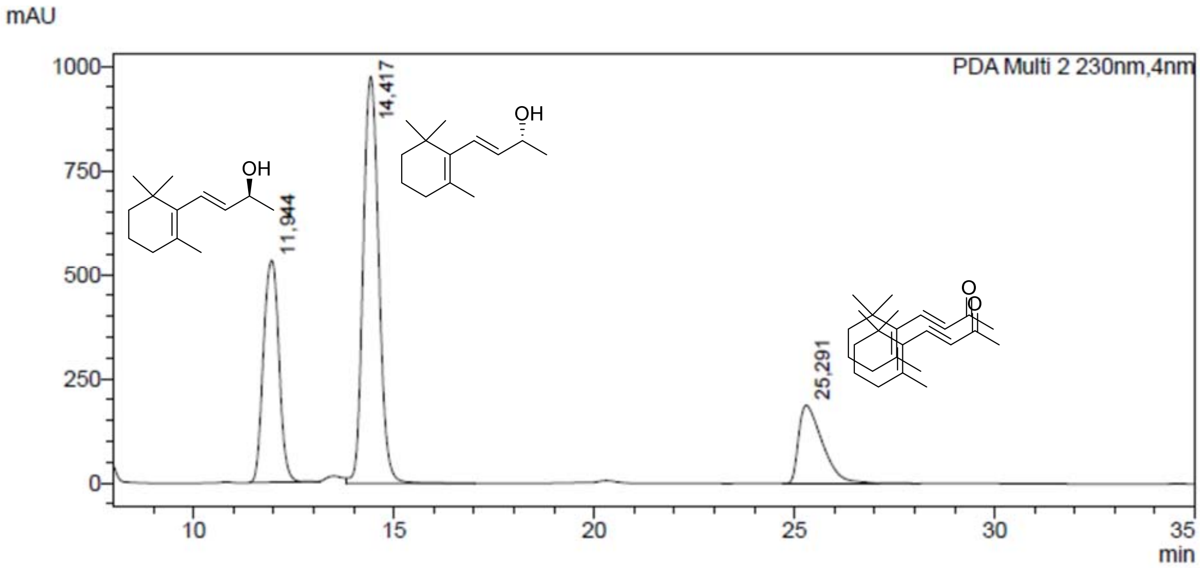

<Peak Table>

PDA Ch2 230nm

Peak#	Ret. Time	Area	Height	Name	Area%
1	11,944	14328066	533018		29,528
2	14,417	26401983	974869		54,410
3	25,291	7794356	187309		16,063
Total		48524405	1695196		100,000

Figure S65. HPLC chromatogram (chiral phase) of oxidation of **5a** by HMFO V465S in the presence of 5% v/v acetone

4.4. GC chromatograms

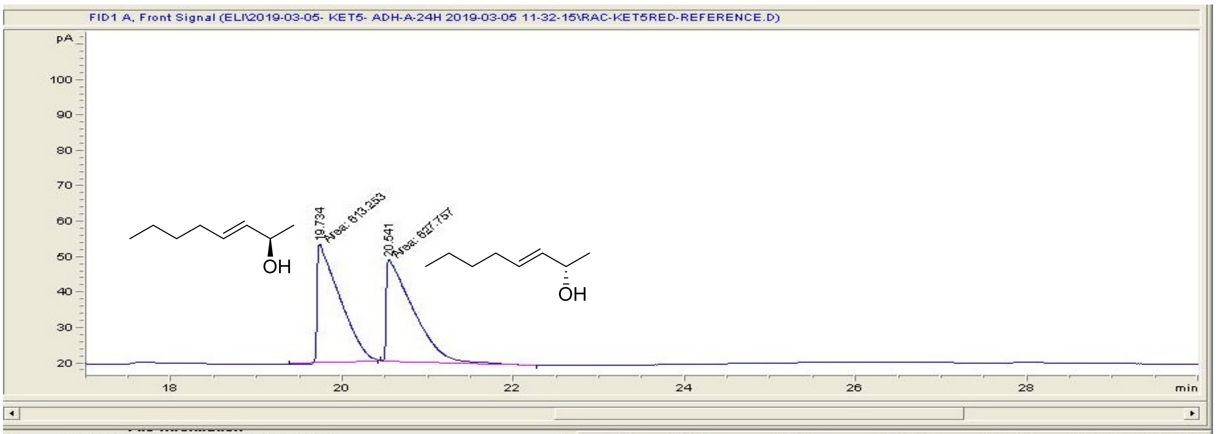

GC-File	RAC-KET5RED-REFERENCE.D
File Path	D:\CHEMSTATION\GC-1\DATA\ELI\2019-03-05- KET5- ADH-A-24
Date	05-Mar-19, 12:16:07
Sample	rac-ket5red-reference
Sample Info	
Barcode	
Operator	Eli
Method	DEX-CB- KET5RED- TRY7-5 µL.M

#	Time	Area	Height	Width	Area%	Symmetry
1	19.734	613.3	33.3	0.3073	49.416	0.133
2	20.541	627.8	28.5	0.3665	50.584	0.107

Figure S66. GC chromatogram (chiral phase) of *rac*-**1a** measured with temperature program 1

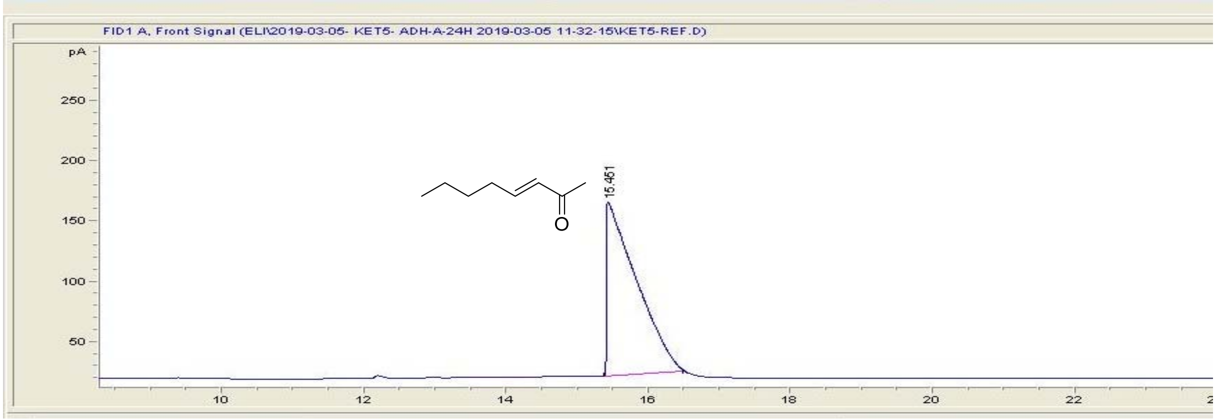

Figure S67. GC chromatogram (chiral phase) of *rac*-**1b** measured with temperature program 1

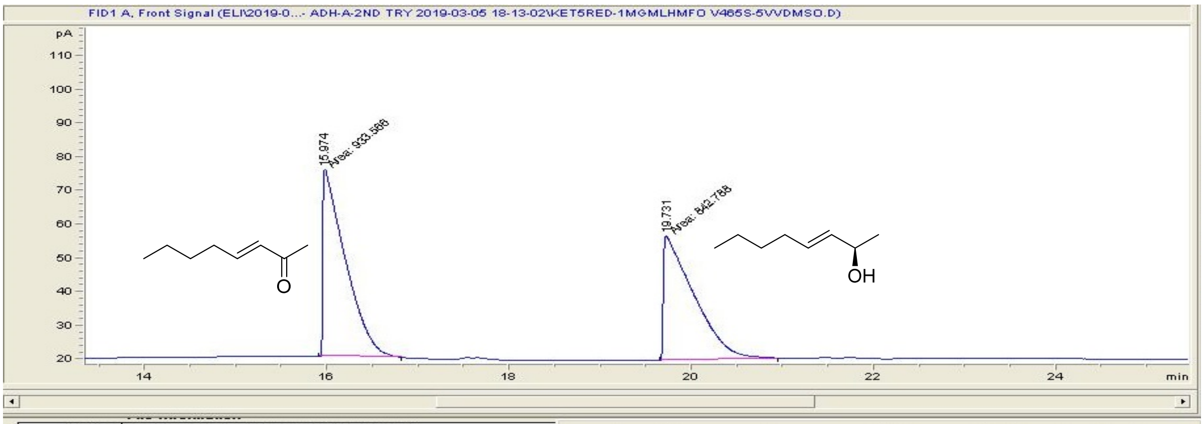

GC-File	KET5RED-1MGMLHMFO V465S-5VVDMSO.D
File Path	D:\CHEMSTATION\GC-1\DATA\ELI\2019-03-05- KET5- ADH-A-2N
Date	05-Mar-19, 23:30:50
Sample	ket5red-1mgmLHMFO V465S-5vvDMSO
Sample Info	
Barcode	
Operator	Eli
Method	DEX-CB- KET5RED- TRY7-5 µL.M

#	Time	Area	Height	Width	Area%	Symmetry
1	15.974	933.6	55.1	0.2822	52.555	0.102
2	19.731	842.8	36.6	0.3842	47.445	8.9E-2

Figure S68. GC chromatogram (chiral phase) of oxidation of **1a** by HMFO V465S in the presence of 5% v/v DMSO measured with temperature program 1

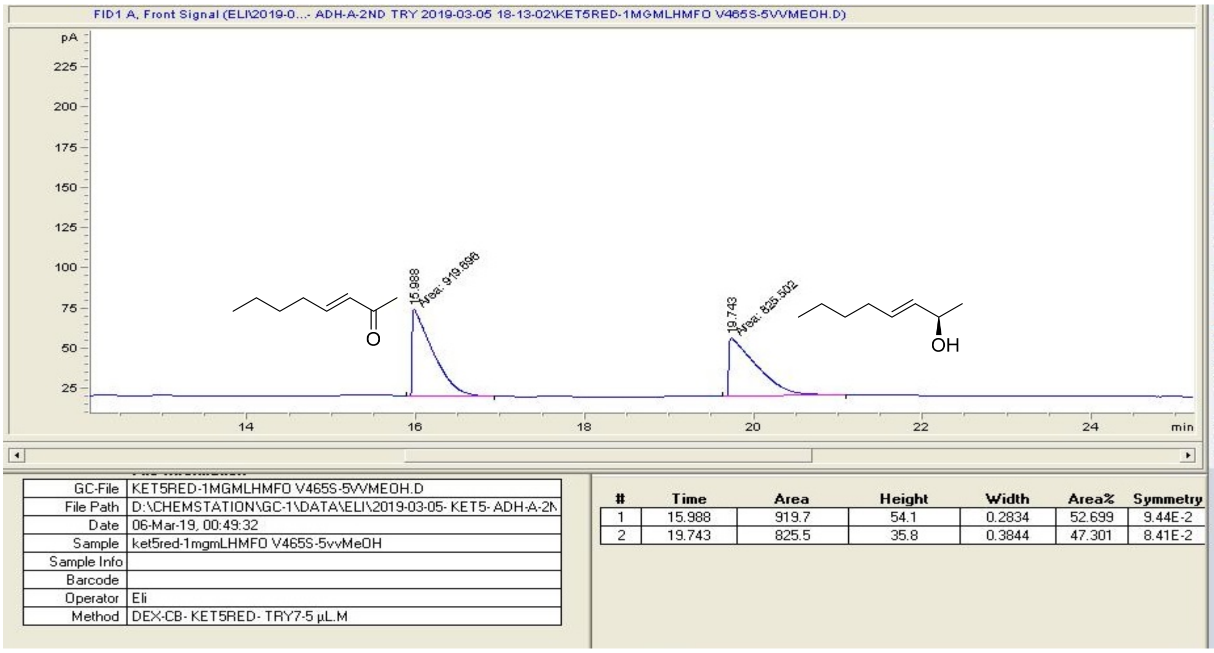

GC-File	KET5RED-1MGMLHMFO V465S-5VVMEOH.D
File Path	D:\CHEMSTATION\GC-1\DATA\ELI\2019-03-05- KET5- ADH-A-2N
Date	06-Mar-19, 00:49:32
Sample	ket5red-1mgmLHMFO V465S-5vvMeOH
Sample Info	
Barcode	
Operator	Eli
Method	DEX-CB- KET5RED- TRY7-5 µL.M

#	Time	Area	Height	Width	Area%	Symmetry
1	15.988	919.7	54.1	0.2834	52.699	9.44E-2
2	19.743	825.5	35.8	0.3844	47.301	8.41E-2

Figure S69. GC chromatogram (chiral phase) of oxidation of **1a** by HMFO V465S in the presence of 5% v/v methanol measured with temperature program 1

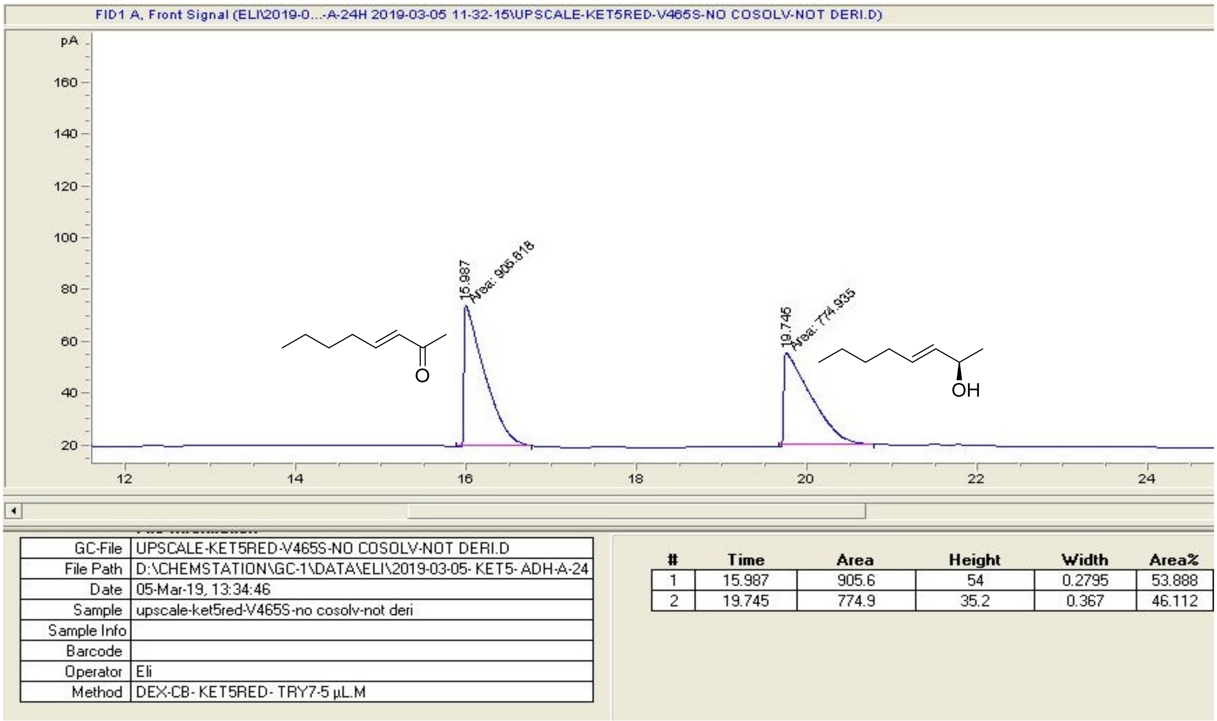

GC-File	UPSCALE-KET5RED-V465S-NO COSOLV-NOT DERI.D
File Path	D:\CHEMSTATION\GC-1\DATA\ELI\2019-03-05- KET5- ADH-A-24
Date	05-Mar-19, 13:34:46
Sample	upscale-ket5red-V465S-no cosolv-not deri
Sample Info	
Barcode	
Operator	Eli
Method	DEX-CB- KET5RED- TRY7-5 µL.M

#	Time	Area	Height	Width	Area%
1	15.987	905.6	54	0.2795	53.888
2	19.745	774.9	35.2	0.367	46.112

Figure S70. GC chromatogram (chiral phase) of oxidation of **1a** by HMFO V465S (upscale) measured with temperature program 1

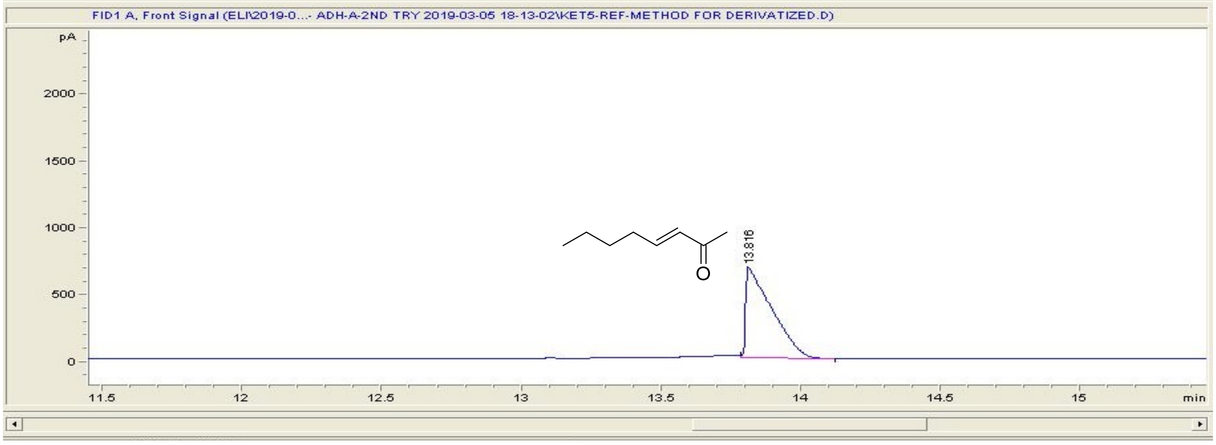

Figure S71. GC chromatogram (chiral phase) of **1b** with temperature program 2

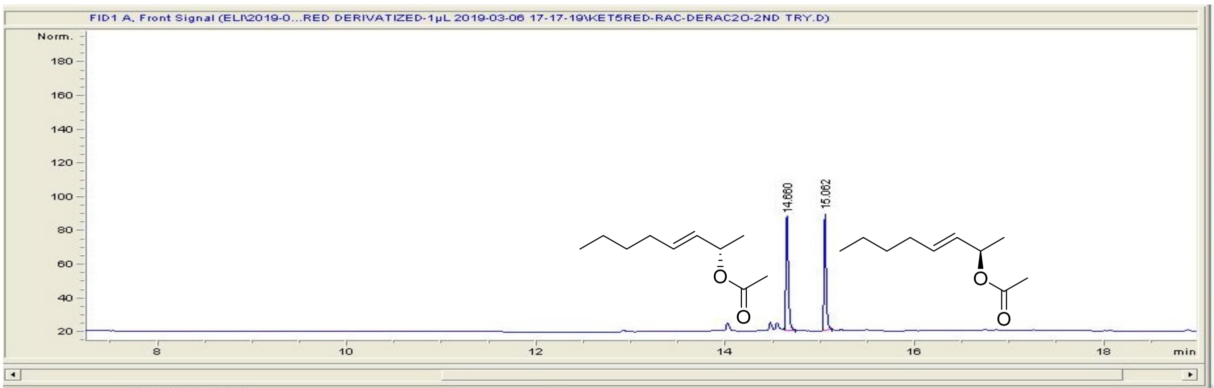

GC-File	KET5RED-RAC-DERAC2O-2ND TRY.D
File Path	D:\CHEMSTATION\GC-1\DATA\ELI\2019-03-05- KET5RED DERI\
Date	06-Mar-19, 17:19:07
Sample	ket5red-rac-derAC2O-2nd try
Sample Info	
Barcode	
Operator	Eli
Method	DEX-CB- KET5RED- DERIVATIZED.M

#	Time	Area	Height	Width	Area%	Symmetry
1	14.66	122	67.6	0.028	50.870	0.69
2	15.062	117.8	69.6	0.0258	49.130	0.698

Figure S72. GC chromatogram (chiral phase) of derivatized **1a** with acetic anhydride with temperature program 2

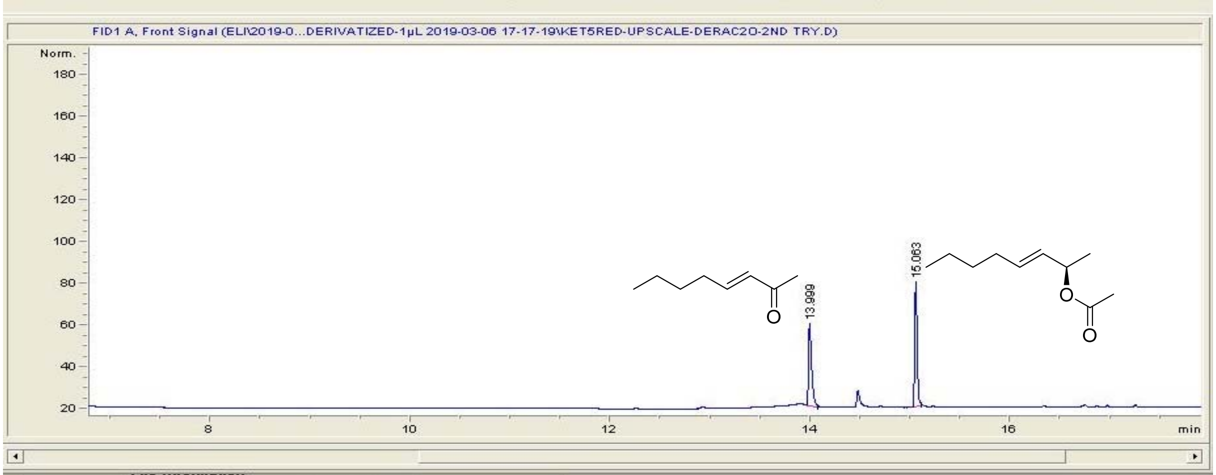

GC-File	KET5RED-UPSCALE-DERAC2O-2ND TRY.D
File Path	D:\CHEMSTATION\GC-1\DATA\ELI\2019-03-05- KET5RED DERI\
Date	06-Mar-19, 17:42:53
Sample	ket5red-upscale-derAC2O-2nd try
Sample Info	
Barcode	
Operator	Eli
Method	DEX-CB- KET5RED- DERIVATIZED.M

#	Time	Area	Height	Width	Area%	Symmetry
1	13.999	85.7	39.4	0.0314	45.744	0.487
2	15.063	101.6	60	0.0268	54.256	0.716

Figure S73. GC chromatogram (chiral phase) of upscale by HMFO V465S and substrate **1a**, which was derivatized with acetic anhydride and measured with temperature program 2

5. References

[1] M. Pickl, C. K. Winkler, S. M. Glueck, M. W. Fraaije, K. Faber, *Molecules* **2017**, *22*.

[2] G. Barker, D. G. Johnson, P. C. Young, S. A. Macgregor, A. L. Lee, *Chem. Eur. J.* **2015**, *21*, 13748-13757.

[3] J. Liu, S. Ma, *Org. Lett.* **2013**, *15*, 5150-5153.

[4] B. Meng, X. Huang, L. Wu, *Adv. Synth. Catal.* **2013**, *355*, 2637-2650.

[5] B. R. Vaddula, A. Saha, J. Leazer, R. S. Varma, *Green Chem.* **2012**, *14*, 2133-2136.

[6] T. Y. Chaudhari, A. Hossian, M. K. Manna, R. Jana, *Org. Biomol. Chem.* **2015**, *13*, 4841-4845.

[7] Y.-C. Liu, Z.-L. Wu, *Chem. Commun.* **2016**, *52*, 1158-1161.

[8] A. Bruneau-Voisine, D. Wang, V. Dorcet, T. Roisnel, C. Darcel, J.-B. Sortais, *Org. Lett.* **2017**, *19*, 3656-3659.

[9] N. J. McLean, A. Gansmuller, M. Concistre, L. J. Brown, M. H. Levitt, R. C. Brown, *Tetrahedron* **2011**, *67*, 8404-8410.

[10] B. Daniel, T. Pavkov-Keller, B. Steiner, A. Dordic, A. Gutmann, B. Nidetzky, C. W. Sensen, E. Van Der Graaff, S. Wallner, K. Gruber, *J. Biol. Chem.* **2015**, *290*, 18770-18781.

[11] S. Pils, K. Schnabl, S. Wallner, M. Kljajic, N. Kupresanin, R. Breinbauer, M. Fuchs, R. Rocha, J. H. Schrittwieser, W. Kroutil, *J. Mol. Catal. B-Enzym.* **2016**, *133*, S6-S14.

[12] X. Chen, H. Zhou, K. Zhang ,J. Li, H. Huang, *Org. Lett.* **2014**, *16*, 3912-3915.

[13] P. He, X. Liu, H. Zheng, W. Li, L. Lin, X. Feng, *Org. Lett.* **2012**, *14*, 5134-5137.

[14] F. Chen, Y. Zhang, L. Yu, S. Zhu, *Angew. Chem.* **2017**, *129*, 2054-2057; *Angew. Chem. Int. Ed.* **2017**, *56*, 2022-2025.

[15] K. Edegger, C. C. Gruber ,T. M. Poessl, S. R. Wallner, I. Lavandera, K. Faber, F. Niehaus, J. Eck, R. Oehrlein, A. Hafner, *Chem. Commun.* **2006**, 2402-2404.
